# Identification of an apiosyltransferase in the plant pathogen *Xanthomonas pisi*

**DOI:** 10.1371/journal.pone.0206187

**Published:** 2018-10-18

**Authors:** James Amor Smith, Maor Bar-Peled

**Affiliations:** 1 Complex Carbohydrate Research Center (CCRC), University of Georgia, Athens, GA, United States of America; 2 Department of Biochemistry and Molecular Biology, University of Georgia, Athens, GA, United States of America; 3 Department of Plant Biology, University of Georgia, Athens, GA, United States of America; Imam Abdulrahman Bin Faisal University, SAUDI ARABIA

## Abstract

The rare branched-chain sugar apiose, once thought to only be present in the plant kingdom, was found in two bacterial species: *Geminicoccus roseus* and *Xanthomonas pisi*. Glycans with apiose residues were detected in aqueous methanol-soluble fractions as well as in the insoluble pellet fraction of *X*. *pisi*. Genes encoding bacterial uridine diphosphate apiose (UDP-apiose) synthases (bUASs) were characterized in these bacterial species, but the enzyme(s) involved in the incorporation of the apiose into glycans remained unknown. In the *X*. *pisi* genome two genes flanking the XpUAS were annotated as hypothetical glycosyltransferase (GT) proteins. The first GT (here on named XpApiT) belongs to GT family 90 and has a Leloir type B fold and a putative lipopolysaccharide-modifying (LPS) domain. The second GT (here on XpXylT) belongs to GT family 2 and has a type A fold. The XpXylT and XpApiT genes were cloned and heterologously expressed in *E*. *coli*. Analysis of nucleotide sugar extracts from *E*. *coli* expressing XpXylT or XpApiT with UAS showed that recombinant XpApiT utilized UDP-apiose and XpXylT utilized UDP-xylose as substrate. Indirect activity assay (UDP-Glo) revealed that XpApiT is an apiosyltransferase (ApiT) able to specifically use UDP-apiose. Further support for the apiosyltransferase activity was demonstrated by in microbe co-expression of UAS and XpApiT in *E*. *coli* showing the utilization of UDP-apiose to generate an apioside detectable in the pellet fraction. This work provides evidence that *X*. *pisi* developed the ability to synthesize an apioside of indeterminate function; however, the evolution of the bacterial ApiT remains to be determined. From genetic and evolutionary perspectives, the apiose operon may provide a unique opportunity to examine how genomic changes reflect ecological adaptation during the divergence of a bacterial group.

## Introduction

Bacteria produce a large array of glycan structures that are associated with the cell surface. Gram-negative bacteria produce peptidoglycan, lipopolysaccharide (LPS), capsular polysaccharides (CPS), and some *N-* or *O-* linked glycoproteins [1, 2], which have been implicated in cellular recognition of the environment and may be important for vital pathways including adherence, motility, and pathogenesis [3]. Synthesis of these glycans requires sugar donors, acceptor substrates, and glycosyltransferases (GTs). GTs are known to have specific substrate specificity and are classified based on conserved three-dimensional fold [4–6] into 105 different families in the Carbohydrate-Active EnZymes (CAZy) database (http://www.cazy.org) [7, 8]. Some GTs necessary to synthesize peptidoglycan have been identified and some crystal structures solved [9–11], and the steps and required machinery for synthesis of LPS have been extensively characterized in *E*. *coli* [12–15]. However, a large number of bacterial glycan structures and GTs that synthesize them remain a mystery. High variability and limited structural data restricts predictability of function, making identification and characterization of GTs responsible for synthesis of bacterial glycan structures challenging.

*Xanthomonas* is an agriculturally and industrially relevant bacterium, as it is pathogenic towards a large range of crops. *Xanthomonas* species secrete an exopolysaccharide (EPS) called xanthan gum, that is used in the food industry as a thickening agent and is implicated in pathogenicity [16, 17]. In addition, the LPS of *Xanthomonas campestris* pv. *campestris* has been shown to influence its virulence [18, 19].

We previously discovered the presence of apiose (3-C-[hydroxymethyl]-D-erythrofuranose, Api) in the soil-dwelling plant pathogen *Xanthomonas pisi* [20] and subsequently isolated and characterized the nucleotide sugar donor UDP-apiose and the enzyme that forms it, UDP-apiose/UDP-xylose synthase (XpUAS), in this bacteria. Evidence of apiose in *X*. *pisi* cell pellet fractions suggested *X*. *pisi* was able to use UDP-apiose to make a cell-surface apioside. In an effort to explain this, we searched for potential genes encoding apiosyltransferase (ApiT) activity.

Here we report the first identification of a gene encoding an apiosyltransferase (ApiT) that is specific to UDP-apiose and a second GT in the same operon that uses UDP-xylose as substrate (XylT). No apiosyltransferase has previously been purified to homogeneity nor have the genes encoding this glycosyltransferase been identified. This is the first report to identify apiosyltransferase activity in bacteria.

## Results

### Identification and phylogenetic analysis of putative glycosyltransferases in apiose operon of *X*. *pisi*

**Fig 1:**
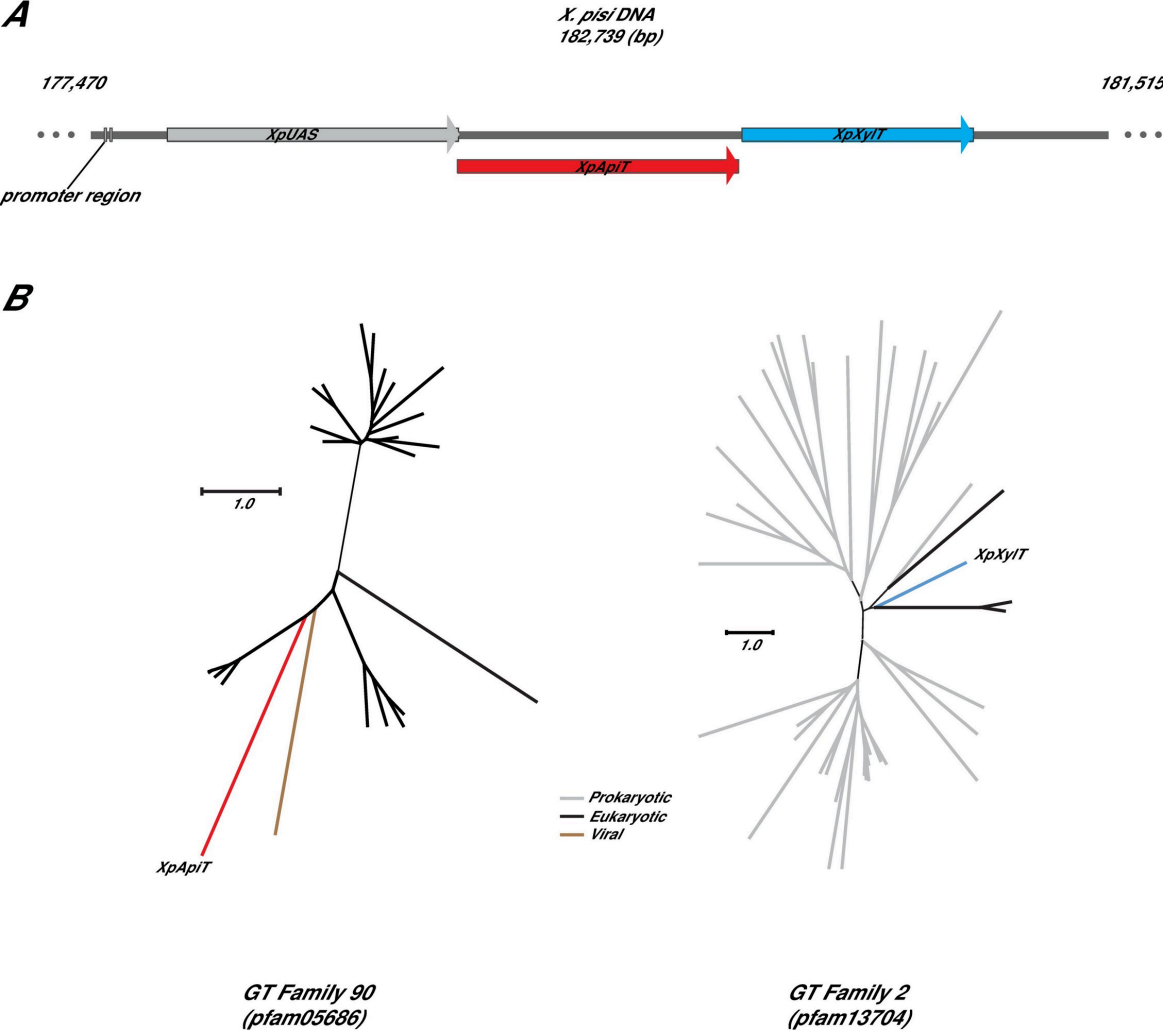
*X*. *pisi* apiose operon and phylogeny of GTs. **(**A) Gene organization of the apiose operon in *X*. *pisi* genome (NZ_JPLE01000032.1) from position 177,470 to 181,515 bp. -35 and -10 promoters were predicted using Bacterial Promoter Prediction website (BacPP, https://molbiol-tools.ca/Promoters.htm). Operon schema was generated using SnapGene Viewer version 3.1.4 (GSL Biotech, Chicago, IL). (B) phylogenetic analysis of XpApiT (PPU69163) and XpXylT (PPU69164). Amino acid sequences from representatives of pfam05686 and pfam13704 selected by the CDD were used. Alignments were made using Clustal Omega [23–25] and the trees generated using Dendroscope [26]. Distance scale represents difference between sequences in substitutions per site. For full amino acid sequence alignment with organism and gene names see S1 and S2 Figs.

Examination of *X*. *pisi* (ATCC 35936) genomic DNA (accession MDEI01000004.1) revealed two hypothetical proteins (accessions PPU69163.1 and PPU69164.1; gene loci XpiCFBP4643_06445 and XpiCFBP4643_06450) directly downstream of UDP-apiose/UDP-xylose synthase (XpUAS) (Fig 1A). The XpiCFBP4643_06445 gene has 4 nucleotides overlapping with the gene encoding XpUAS, and a gap of 11 nucleotides containing a putative ribosomal binding sequence (RBS) between XpiCFBP4643_06445 and XpiCFBP4643_06450, suggesting the genes are organized in an operon. BLAST [21] analyses (BLASTp and tBLASTn) of XpiCFBP4643_06445 gene product (PPU69163) revealed few proteins that align. The closest homolog is a hypothetical protein from *Pleurocapsa sp*. CCALA 161 (accession WP_106240036.1) that shares low sequence identity with an e-value of 2e^-21^. Additionally, sequence analysis using profile hidden Markov Models (HMMER; www.ebi.ac.uk/Tools/hmmer) [22] did not reveal close homologs with more than e^-27^. Interestingly, we noticed that XpiCFBP4643_06445 had very low homology e^-19^ over 55% coverage to a protein belonging to pfam05686 classified as glycosyl transferases belonging to GT90. Based on the CAZy GT database, proteins in GT90 had a putative type B fold. The third gene in the operon, XpiCFBP4643_06450, was annotated to encode a protein (PPU69164) with a domain shared by GT family 2 (pfam13704) and had a putative type A fold.

The BLAST and HMMER programs [21, 22] were further used to identify bacterial proteins in the NCBI non-redundant database that share sequence similarity to PPU69163 and PPU69164. The hits with highest sequence identity to PPU69164 were from *Synechococcus sp*., *Desulfonatronum thiosulfatophilum*, and *Methylobacterium sp*. Those with highest sequence identity to PPU69163 were all below 35%, but include proteins from *Mycena chlorophos*, *Pleurocapsa sp*., and *Sphingomonadaceae*. Based on the results we obtained in this report from here on PPU69163 is named XpApiT and PPU69164, XpXylT.

Unrooted phylogenetic trees were generated for representative members of GT family 2, including XpXylT and members of GT family 90, including XpApiT (Fig 1B). XpXylT clusters into a clade with the predicted procollagen galactosyltransferase 1 precursor from Norway rat *Rattus norvegicus* and predicted proteins from other eukaryotic members including a sea anemone and a marine diatom. XpApiT is in its own unique clade indicating its distinction from the closest annotated tree members; a predicted viral protein from *Gryllus bimaculatus iridovirus* and human and zebrafish KDEL1 (Fig 1B).

The analyses compared alignments of amino acid sequences of representatives from respective GT families (S1 and S2 Figs). Since most of these representatives are uncharacterized, and because there is low sequence identity between the *X*. *pisi* GT representatives and their respective GT family members, these GTs were further investigated to define their activies. Because a genetically tractable strain of *X*. *pisi* has not yet been established and previous work has optimized an inducible expression system for XpUAS [20], XpApiT and XpXylT were heterologously expressed in *E*. *coli* and functionally characterized against a screen of potential UDP-sugar donors.

### Initial determination of XpXylT and XpApiT glycosyltransferase activities

**Fig 2:**
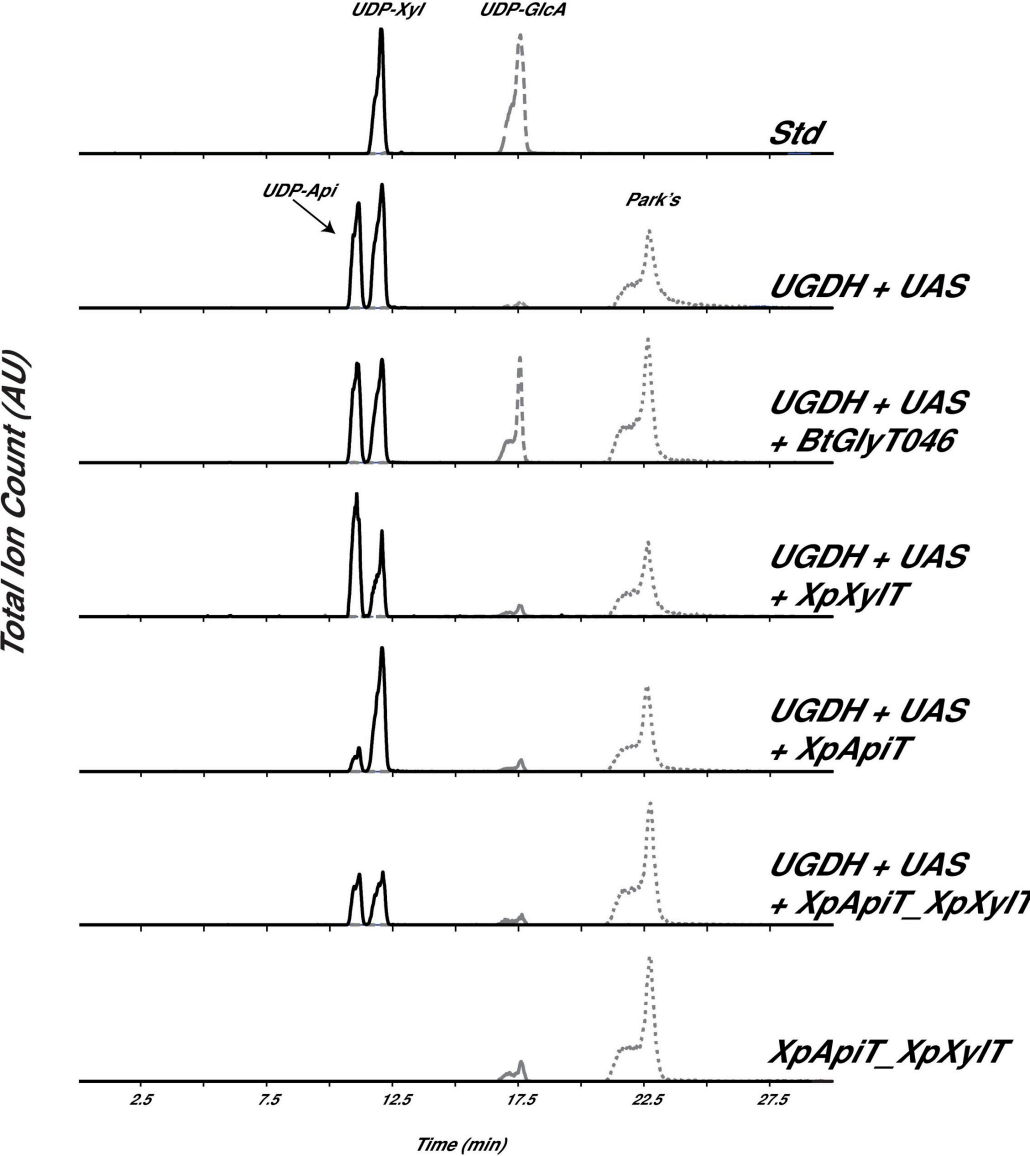
In microbe activity of XpXylT and XpApiT. Analysis of in microbe nucleotide sugars by HILIC-LC-ESI-MS/MS. (A) top panel elution of standard (Std): UDP-GlcA and UDP-Xyl; Nucleotide sugars were extracted from *E*. *coli* cells induced to express genes encoding BtbUGDH and SpUAS only; BtbUGDH and SpUAS along with a putative N-acetyl-glucosamine transferase from *Bacillus thuringiensis* (BtGlyT046), XpXylT, XpApiT, or XpApiT with XpXylT; or only XpApiT with XpXylT as control (bottom panel). Extracted ion chromatograms (XICs) of [M-H]^-^ ions diagnostic for UDP-pentose (*m/z* 535.0, solid line), UDP-hexuronic acid (*m/z* 579.0, dashed line) and Park’s nucleotide (*m/z* 595.6, dotted line) are displayed. Park’s nucleotide is a UDP-MurNAc-pentapeptide that is used as an internal standard for nucleotide-sugar detection as it is abundantly made in *E*. *coli*.

To investigate if these GTs use nucleotide sugars as substrates, in particularly UDP-apiose (UDP-Api) and UDP-xylose (UDP-Xyl), the coding sequences of PPU69163 and PPU69164 were cloned into modified pET28b *E*. *coli* expression vector [27]. Since UDP-apiose has a relatively short lifespan and naturally degrades within 4 h to apiofuranosyl-1,2-cyclic-phosphate [28, 29] we set up a ‘reporting system’ to determine the consumption of UDP-apiose *in vivo*. For these experiments (we named herein in microbe) pET plasmids containing XpXylT or XpApiT alone, and a pET plasmid containing both XpXylT and XpApiT together were co-transformed with a pCDF plasmid containing both a UDP-glucose dehydrogenase (UGDH) [30] and a UDP-apiose synthase (UAS) to provide the potential UDP-apiose and UDP-xylose substrates for the GTs [20]. Cell extracts from the isopropyl β-D-thiogalactoside (IPTG)-induced *E*. *coli* cells were processed and examined for presence and relative amounts of UDP-Api and UDP-Xyl. Peaks corresponding to UDP-Api and UDP-Xyl were compared (Fig 2).

In cells co-expressing UAS with XpXylT, there is a noticeable decrease in the relative amount of UDP-Xyl extracted and no change in the amount of UDP-Api (Fig 2), while there is no such decrease in control samples. This suggests that XpXylT consumes UDP-xylose as a substrate, perhaps transferring it to a substrate yet to be defined. Surprisingly, cells co-expressing UAS with XpApiT show a marked decrease in UDP-Api and no change in UDP-Xyl when compared to controls (Fig 2). The decrease in UDP-Api in cells expressing XpApiT indicates XpApiT is likely specifically utilizing UDP-Api in microbe. This suggests that XpApiT consumes UDP-apiose as a substrate, perhaps transferring it to a substrate yet to be defined. There is also possibility that in the absence of ‘true acceptor’ (as in the *in vivo* assays carried out in *E*. *coli* and not in the native environment of *X*. *pisi*) that the glycosyltransferase may act to hydrolyze UDP-sugar substrate [31–34]. Extracts from cells expressing both XpXylT and XpApiT along with UAS have less UDP-Xyl and UDP-Api relative to the internal Park’s nucleotide (UDP-MurNAc-pentapeptide) than controls (Fig 2). To further explore the specific activity of these two GTs, His-tag recombinant proteins were expressed and purified and *in vitro* assays were developed.

### Purification of XpXylT and XpApiT and in vitro assay

**Fig 3:**
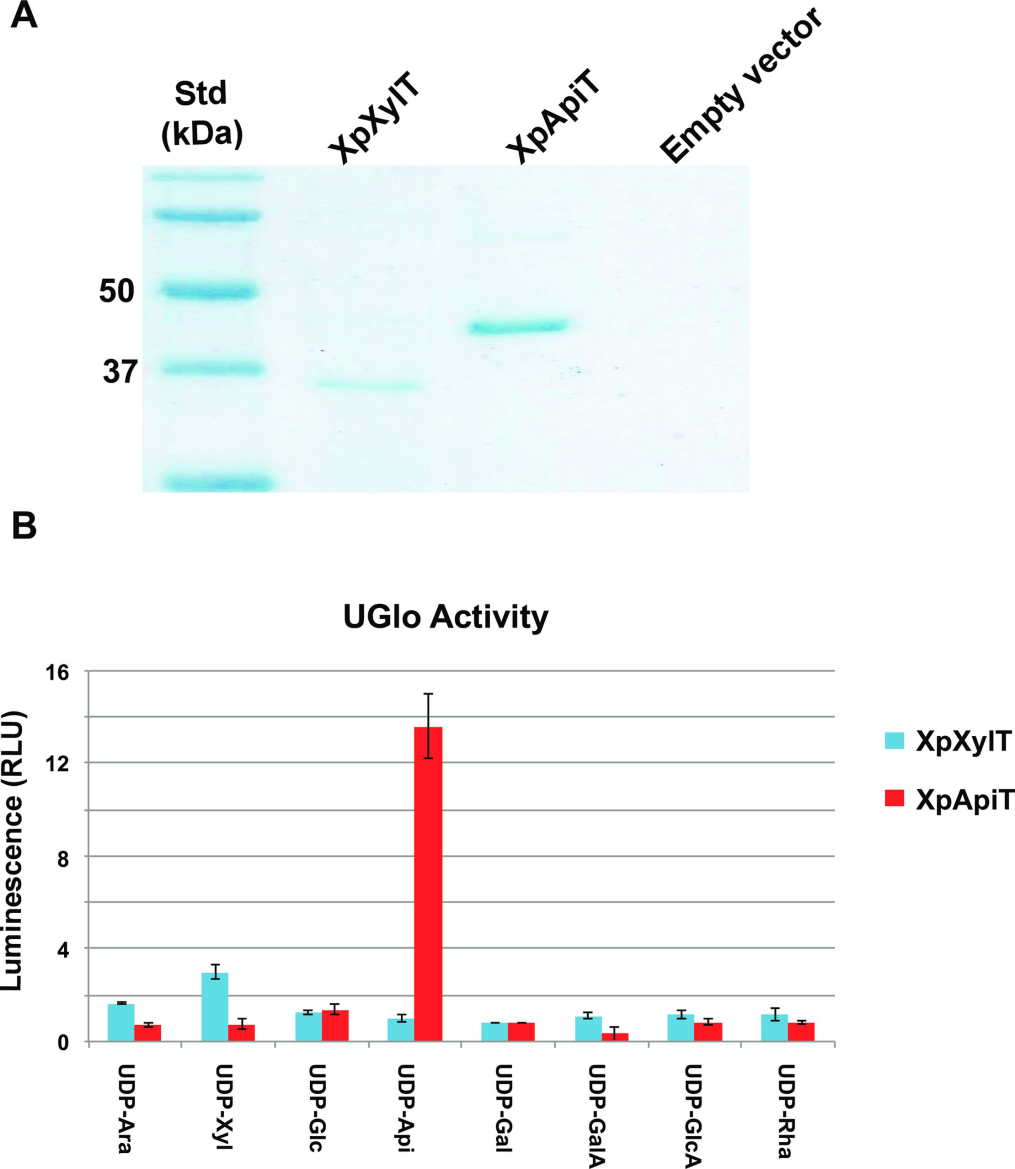
Activity of purified XpXylT and XpApiT proteins. (A) Nickel-purified proteins from *E*. *coli* cells induced to express XpXylT, XpApiT, and empty vector control with expected sizes of XpXylT and XpApiT: 32.9 and 41.4 kDa, respectively. (B) Bar graph of relative luminescence from UDP-Glo assays. Assays of 50 mM potassium phosphate pH 7.5, 0.1 mM UDP-sugar, 0.5 μg enzyme in 10 μl total volume reacted at room temperature for 1–1.5 hr. Equal volume of UDP-Glo detection reagent added to stop reaction for 1 hr. Relative luminescence is fold signal over background; measured by subtracting blank (enzyme without UDP-sugar) and dividing by background (UDP-sugar with boiled enzyme). Data is the average of three independent samples and displayed with calculated standard error (SE) bars.

To obtain additional evidence for the specific activities of XpXylT and XpApiT, the recombinant His_6_-tagged proteins were solubilized from *E*. *coli* cells and purified using nickel-affinity column. The recombinant XpXylT (with theoretical mass 32.9 kDa) and XpApiT (41.4 kDa) migrated on SDS-PAGE with predicted masses (Fig 3A).

Reactions of recombinant XpApiT with UDP-Api have over ten-fold more luminescence than other UDP-sugar substrates, indicating its specificity for UDP-Api (Fig 3B). Reactions of recombinant XpXylT with UDP-sugar substrates demonstrate a 3-fold increase in luminescence with UDP-Xyl. The UDP-Glo Kit (Promega) was used to test the indirect activities of each GT (see methods). In order to develop an ApiT assay, UDP-Api substrate had to be generated quickly *in vitro*, HPLC purified, and flash frozen (see methods). To determine the specific NDP-sugar donor substrate of each GT, a UDP-sugar substrate screen was initially developed, to which purified enzyme was added. Because the acceptor substrate(s) were unknown, reactions were allowed to hydrolyze UDP-sugar substrate using water as the acceptor [34] and reaction product analyzed by luminometer according to manufacturer’s instructions.

### UDP-apiose synthesis and purification

**Fig 4:**
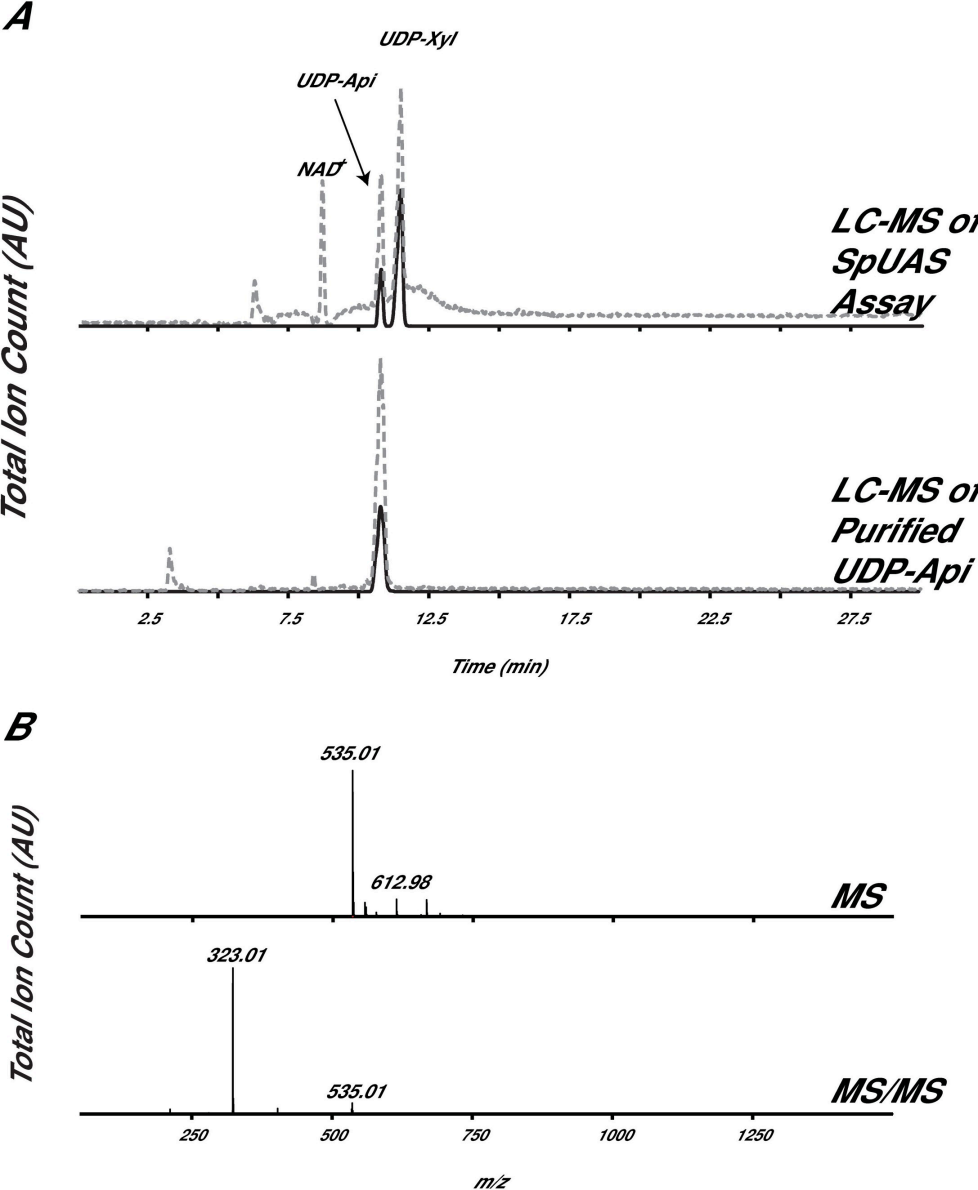
HPLC-purified *in vitro* synthesized UDP-apiose. (A) Total ion count (TIC, dashed line) and XIC [M-H]^-^ ions diagnostic for UDP-pentose (*m/z* 535.0, solid line). (B) Average of all ions at 11.0 min (MS, top panel) and average of all fragmented ions at 11.0 min (MS/MS, bottom panel).

Generation of pure UDP-Api substrate is not trivial due to the fact that it spontaneously degrades into an apiofuranosyl-1,2-cyclic-phosphate in solution [28, 29]. Generation of UDP-Api required *in vitro* synthesis using an active recombinant UDP-apiose/UDP-xylose synthase, UAS [29]. The UAS reaction products were quickly separated over HILIC HPLC, and the UDP-Api peak was collected, flash-frozen in liquid N_2_ and saved at -80 °C. This methodology allowed us to control and reduce the native degradation of UDP-Api. To validate purity and relative stability of UDP-Api, a small amount of freshly prepared UDP-Api was diagnosed by LC-MS (Fig 4A). The data shown (Fig 4B) provide evidence that UDP-Api is intact and lacking UDP-Xyl product contamination. Pure UDP-Api was necessary to screen XpXylT and XpApiT for activity using the UDP-Glo assay. Without UDP-Api, no activity would be assigned to XpApiT.

### In microbe production of apiosides

To further investigate if the XpXylT and XpApiT were actively synthesizing glycans in microbe, engineered *E*. *coli* cultures were fractionated and analyzed for the presence and abundance of xylose and apiose. Bacteria grown in liquid media were pelleted and subjected to aqueous-methanol:chloroform extractions to lyse cells and separate hydrophilic small molecules and hydrophobic lipid moieties from the cell surface (termed pellet) fraction. Pellets were successively washed with water and dried. Samples were chemically hydrolyzed and monosaccharides converted to their alditol-sugar derivatives for gas chromatography tandem mass spectrometry (GC-MS) analysis.

**Fig 5:**
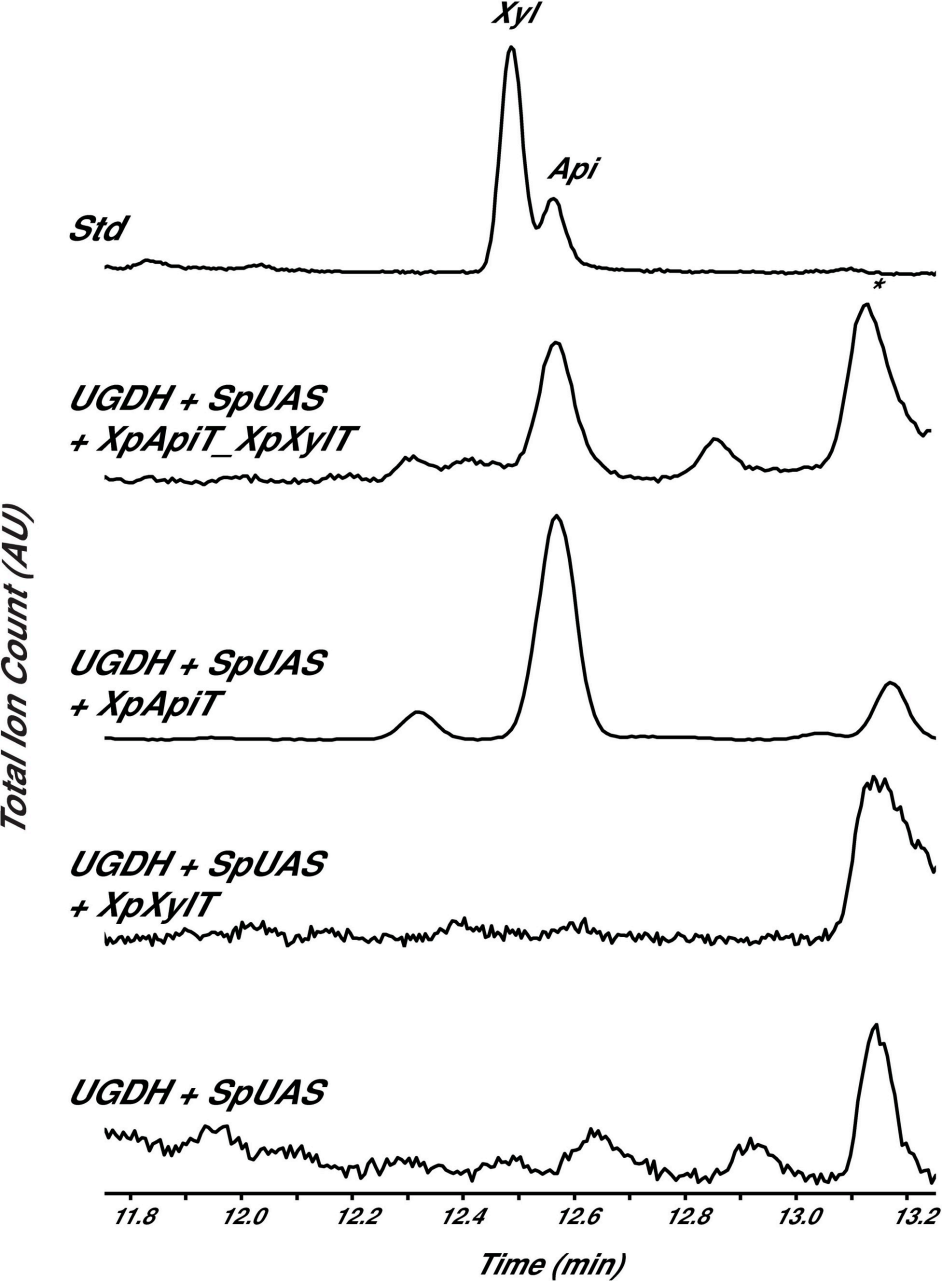
Detection of apiose in microbe. GC-MS analysis of alditol-acetate derivatives from cell pellet fractions of *E*. *coli* engineered to overexpress BtbUGDH + SpUAS with *X*. *pisi* GTs in microbe. Standard (Std) contains authentic xylose and apiose. The region of the total ion count for xylose- (Xyl) and apiose- (Api) alditol-acetate derivatives is expanded. * indicates unidentified residue, but not a sugar.

The pellet fractions of only the UGDH+UAS+XpApiT and UGDH+UAS+XpApiT_XpXylT strains had a peak that migrated like apiose (Fig 5). The electron impact MS fragmentation pattern of this peak’s structure was similar to authentic apiose; for example, the major diagnostic peak at *m/z* 188 [20]. Apiose was not detected in the UGDH+UAS+XpXylT, UGDH+UAS, or XpApiT_XpXylT control strains, and no xylose was detected in any of the pellet fractions. In addition to apiose, these extracts also consist of glucose, galactose, and arabinose sugar residues (Table 1).

**Table 1:** Glycosyl-residue compositions of microbe pellets.

	UGDH+UAS	UGDH+UAS +BtbGlyT046	UGDH+UAS + XpXylT	UGDH+UAS +XpApiT	UGDH+UAS +XpApiT_XpXylT	XpXylT	XpApiT	XpApiT_XpXylT
	Monosaccharide (mol %)							
Rib	83.2 ± 1.0	85.5 ± 3.2	90.2 ± 1.2	50.3 ± 3.5	56.6 ± 5.9	89.0 ± 1.4	86.5 ± 2.0	82.1 ± 2.2
Ara	1.0 ± 0.2	1.1 ± 0.2	0.7± 0.03	0.6 ± 0.1	0.9 ± 0.4	0.7± 0.05	0.6± 0.1	0.9± 0.01
Xyl	N.D.	N.D.	N.D.	N.D.	N.D.	N.D.	N.D.	N.D.
Api	N.D.	N.D.	N.D.	2.78 ± 0.4	1.42 ± 0.4	N.D.	N.D.	N.D.
Man	0.4 ± 0.2	0.3 ± 0.2	0.3 ± 0.1	0.4 ± 0.04	0.4 ± 0.2	0.4 ± 0.1	0.6± 0.01	0.8± 0.02
Gal	3.9 ± 0.2	4.2 ± 0.2	4.3± 0.8	41.2 ± 4.5	35.1 ± 2.2	5.7± 0.8	7.1± .6	4.1± 0.4
Glc	2.2 ± 0.2	1.0 ± 0.1	1.7± 0.3	1.2 ± 0.03	1.0 ± 0.04	1.7± 0.2	3.1± 0.04	2.4± 0.3
GlcN	2.0 ± 0.1	1.2 ± 0.3	1.1 ± 0.1	1.0 ± 0.7	0.9 ± 0.3	0.7± 0.2	.9 ± 0.02	2.1 ± 0.2
GlcNAc	8.2 ± 0.3	7.0 ± 0.1	2.4± 0.3	2.2 ± 0.07	3.1 ± 0.13	2.0 ± 0.2	1.1 ± 0.3	7.9 ± 1.1

The glycosyl residue composition (mol%) was determined by GC-MS analysis of alditol-acetate derivatives generated from the pellet fraction of transformed *E*. *coli*. Data is the average ± standard error of three independent samples. N.D. indicates not detected.

## Materials and methods

### Bacterial strains

*Xanthomonas pisi* ATCC 35936 (*X*. *pisi*) was obtained from the American Type Culture Collection in Manassas, VA, USA. This specific *X pisi* strain was isolated from *Pisum sativum* in Japan (by Goto and Okabe, 1958[35]) [36] and its genome recently sequenced (GenBank DNA accession MDEI01000004.1). Unless otherwise stated, *X*. *pisi* cultures were grown on nutrient agar (BD Difco; Franklin Lakes, NJ, USA) at 25 ^o^C. Liquid cultures were grown in 250 mL of nutrient broth, shaking at 250 rpm. *E*. *coli* cells used were Bl21(De3)-derived cells (Novagen, Madison, WI, USA).

### Identification and cloning of XpXylT and XpApiT

The genomic DNA surrounding bUAS of *X*. *pisi* (10,000 bp upstream and 10,000 downstream) was examined for in-frame amino acid-coding sequences and analyzed by BLASTX program [21]. Resultant *X*. *pisi* predicted proteins WP_046963858.1 (locus tag XpiCFBP4643_06445, protein i.d. PPU69163; here on named XpApiT) and WP_0052764980.1 (locus tag XpiCFBP4643_06450, protein i.d. PPU69164; here on named XpXylT) were analyzed by BLASTP against the NCBI non-redundant database to obtain conserved domains from the Conserved Domain Database (CDD). XpApiT had conserved CAP10 (smart00672) and GT family 90 (pfam05686) domains, and XpXylT was shown to contain conserved GT family 2 (pfam13704), β-4-glucosyltransferase (cd02511), and WcaA (COG0463) domains.

RNA was extracted as described [37] from a 2-day-old culture of *X*. *pisi* grown in liquid media and the XpXylT, XpApiT, and XpApiT_XpXylT cDNA’s were amplified, using forward and reverse primers (IDT; Coralville, IA, USA; S1 Table). The PCR product was cloned into the *E*. *coli* expression vector pET28b modified to contain an N-terminal His_6_ tag followed by a TEV cleavage site [27].

Following cloning of the individual *X*. *pisi* GT genes, the plasmids were sequence verified (Eurofins, LUX) and termed, pET28b-TEV-XpXylT.2, pET28b-TEV-XpApiT.1 and pET28b-TEV-XpApiT_XpXylT.7. The amino acid sequences for XpXylT and XpApiT were deposited in GenBank (accessions MH673349 and MH673348, respectively).

### Analysis of nucleotide sugars produced in microbe

Nucleotide sugars from *E*. *coli* harboring the expression plasmids were harvested as described [29, 38]. *E*. *coli* cells co-transformed with pCDFDuet-SpUAS_BtbDH and either empty pET28b vector control (UGDH+UAS), pET28b-TEV-XpXylT.2 (UGDH+UAS+XpXylT), pET28b-TEV-XpApiT.1 (UGDH+UAS+XpApiT), pET28b-TEV-XpApiT_XpXylT.7 (UGDH+UAS+XpApiT_XpXylT) or negative control pET28b-TEV-BtbGlyT046 (UGDH+UAS+BtbGlyT046), and cells co-transformed with empty pCDFDuet vector control and either pET28b-TEV-XpXylT.2, pET28b-TEV-XpApiT.1, and pET28b-TEV-XpApiT_XpXylT.7 alone were grown in LB medium [1.0% (w/v) Bacto tryptone, 0.5% (w/v) Bacto yeast extract, and 1.0% (w/v) NaCl] supplemented with 35 μg/ml chloramphenicol, 50 μg/ml kanamycin, and 25 μg/ml spectinomycin at 37 °C and 250 rpm, induced with Isopropyl β-D-thiogalactoside (IPTG, 0.5 mM) at an OD_600_ of 0.6 and grown at 30 °C for 4 h. In microbe nucleotide sugars were extracted and analyzed by hydrophilic interaction liquid chromatography electrospray ionization tandem mass spectrometry (HILIC-LC-ESI-MS/MS) as described [29]. Briefly, in microbe extracts were mixed with 2/3 volume aq. 95% acetonitrile (ACN) containing 25 mM ammonium acetate and an aliquot (10–20 μl) chromatographed over an Accucore amide-HILIC column (150 x 4.6 mm; Thermo Fisher Scientific; Waltham, MA, USA), eluted at 0.4 ml min^-1^ with a linear gradient of aq. 75% (v/v) acetonitrile containing 40 mM ammonium acetate, pH 4.4, to 50% (v/v) acetonitrile containing 40 mM ammonium acetate, pH 4.4, over 35 min [39] using a Shimadzu (Kyoto, JPN) LC-30AD HPLC. Mass spectra (mass range 100–2,000 *m/z*) were collected every 1.3 sec for 30 minutes. Second stage MS/MS data was collected by collision-induced dissociation (CID) with collision energy of 35% and a nebulizing nitrogen gas flow of 1.5 ml min^-1^.

### His_6_-tagged protein expression and purification

*E*. *coli* cells transformed with pET28b-TEV-XpXylT.2, pET28b-TEV-XpApiT.1, or the empty vector control were grown, induced, and protein was extracted and purified over Ni-column as described [27]. The final soluble protein fraction (Fraction S20) was collected and kept on ice prior to immediate purification. The different His_6_-tagged proteins, including control empty plasmid were separated SDS-PAGE [12% (w/w) polyacrylamide] and stained. Protein concentrations were determined with the Bradford reagent [40] using bovine serum albumin (BSA) as standard.

### Generation of UDP-apiose

Reactions (50 μl) of 1 mM UDP-GlcA with 10 μg of purified SpUAS and 0.5 mM NAD^+^ were carried out, separated over an Accucore amide-HILIC column (150 x 4.6 mm; Thermo), as described above [29]. Nucleotides sugars were detected by their A_261nm_ (for UDP-sugars) and A_259nm_ (for NAD^+^) using an Agilent (Santa Clara, CA, USA) 1100 Series HPLC equipped with a G1313A auto-sampler, a G1315B diode array detector, and ChemStation software. UDP-apiose eluted at ~11.2 min, was collected, and aliquots were flash-frozen in liquid N_2_ and saved at -80 °C. To validate purity and relative stability of UDP-Api, a small amount of freshly prepared UDP-Api was diagnosed by on LC-MS (see above).

### Recombinant XpXylT and XpApiT enzyme assays

Unless otherwise indicated, the 10 μl UDP-Glo (Promega; Madison, WI, USA; http://www.promega.com) reactions were performed in 50 mM potassium phosphate pH 7.5, containing 0.1 mM UDP-sugar 1 μg of purified protein. The assay mixtures were incubated at room temperature for up to 1.5 h. The reactions were terminated by addition of an equal volume of UDP-Glo Detection Reagent in white polystyrene 384-well assay plates (Corning; Corning, NY, USA) and incubated for 1 h at room temp. Luminescence was measured using a POLARstar OPTIMA multifunctional microplate reader (BMG Labtech; Ortenberg, DEU). Relative luminescence was calculated by subtracting blank, containing enzyme without UDP-sugar substrate, and dividing by background, UDP-sugar with boiled enzyme.

### Glycosyl residue composition analysis

*E*. *coli* cultures (250 ml; 5 to 18-hr post culturing) were centrifuged (10,000 g, 5 min, 4 °C) and cell pellets suspended in 10 volumes of cold MeOH:chloroform:H_2_O (4:4:2, v/v/v). The suspensions were transferred to 15 ml falcon tubes and vortexed for 10 min (30 sec every 2 min, 4 °C). The suspensions were centrifuged (10,000*g*, 5 min, 4 °C) and separated into a top methanolic-water phase (termed methanolic), medial interphase (termed pellet) and bottom organic chloroform phase. A portion (20 μl) of the top methanolic fraction was analyzed on HILIC-LC-ESI-MS/MS (see above). The bottom chloroform fraction was transferred to a separate tube. The remaining interphase was re-suspended in 2 ml DDW and samples centrifuged (10,000 g 5 min, 4 °C). Supernatant was again washed by re-suspension in 2 ml DDW and centrifugation and supernatant vacuum aspirated. The pellet was again re-suspended in 1 ml DDW and transferred to a 13 mm borosilicate tube.

In microbe pellets, methanolic and organic solvent extracts or standards (~1 mg) were supplemented with myo-Inositol (10 μl of 5 mM solution) as an internal standard, evaporated to dryness at room temperature using a stream of air (REACTIVAP III, Thermo) and then hydrolyzed for 1 h at 120 °C with 1 ml of 1 M TFA. TFA was removed by evaporation and the released monosaccharides were then converted into their corresponding alditol-acetate derivatives according to York *et al* [41], and the final residue dissolved in acetone (100 μl). A fraction (1 μl) of each of the alditol-acetate derivative samples was analyzed by gas-liquid chromatography as described [29].

## Discussion

Here we describe the first evidence of an apiosyltransferase able to specifically use UDP-apiose as substrate. We show that the *X*. *pisi* genome consists of an operon with genes encoding UDP-apiose synthase (XpUAS), an apiosyltransferase (XpApiT), followed by another enzyme that uses UDP-xylose (XpXylT). The apiosyltransferase belongs to CAZy GT family 90 that consists of fungal xylosyltransferases [42–44] and animal protein *O*-glucosyl- and xylosyltransferases that have been biochemically characterized.

The C-terminal portion (~200 aa) of the apiosyltransferase, XpApiT, consists of a CAP10 domain (smart00672) [45] and is a GT family 90 (pfam05686) [43] as classified by the Conserved Domain Database (CDD) [46]. The CAP10 from the fungal, *Cryptoccocus neoformans* is involved in capsular-polysaccharide formation and virulence [45]. Since discovery of this domain, capsular-associated proteins of *C*. *neoformans* have been classified into GT family 90 and characterized as β-1,2-xylosyltransferases that modify the capsular polysaccharide as well as some glycosphingolipids [42–44]. Proteins homologous to CAP10 have been identified in plants and animals and are described in the CDD as “putative lipopolysaccharide-modifying enzymes” [46]. In *Drosophila melanogaster*, Rumi (AAN13920.1) was identified and characterized as a soluble, ER-localized, CAP10-domain-containing protein involved in protein *O*-glycosylation and *O*-xylosylation critical to Notch signaling [47, 48]. Additional homologs have been characterized in human and mouse [49–51]. Because XpApiT is classified into GT family 90, we hypothesized that it may be involved in glycosylation of extracellular polysaccharide(s).

Interestingly, there are plant proteins classified into GT family 90 that are uncharacterized, including at least 8 *Arabidopsis* DUF821 proteins. Because plants synthesize secondary metabolite apiosides such as apiin from parsley and wall apiosides such as rhamnogalacturonan II (RG-II) and apiogalacturonan (ApiGalA) from *Lemna*, plants must contain enzymes with ApiT activity. In fact, prior work has demonstrated that UDP-Api is the activated nucleotide sugar used by parsley cell extracts and *Lemna* for the incorporation of apiose into apiin and apiogalacturonan, respectively [52, 53]. No apiosyltransferase had previously been purified to homogeneity nor had the genes encoding this glycosyltransferase been identified. It is possible that future characterization of the plant GT family 90 (DUF821) enzymes will reveal their function in apiosylation.

XpXylT is classified as GT family 2 in CAZy, which contains over 120,000 bacterial proteins and may be the largest GT family in the database. GT family 2 also contains cellulose synthase (CESA) from plants [54]. The N-terminal portion (~100 aa) of XpXylT is classified as containing β-4-glucosyltransferase (cd02511) [7, 8, 55–59] and WcaA (COG0463) [60] conserved domains by the Conserved Domain Database (CDD) [46]. WcaA is a GT involved in synthesis of the exopolysaccharide (EPS) colonic acid (CA) produced by E. coli [60]. Domain cd02511 contains GTs important for synthesis of the lipooligosaccharide (LOS) cores of the bacteria *Neisseria meningitidis* [56] and *Campylobacter jejuni* [55], and domain pfam13704 are described as putative prokaryotic glucosyltransferases [46]. Based on these classifications, we hypothesize that XpXylT may be involved in EPS or LOS synthesis.

Evolution of this unique apiose operon is not straightforward. The apiose operon appears to be exclusive to *X*. *pisi* based on current genomic data. No homologs to XpUAS, XpApiT, or XpXylT have been found in other *Xanthomonas* species. *X*. *pisi* may have independently evolved a way to synthesize and use UDP-apiose from precursor genes. Alternatively, the genes may be a result of horizontal gene transfer. Indeed, there is evidence that *Xanthomonas* pathovars are able to rapidly diversify by genomic rearrangement and diversification from common ancestral genes [61]. We propose that acquiring the Api operon was due to environmental interactions and ecological adaptation beneficial for *X*. *pisi*, but the source of the genes in the Api operon remain at current unclear. We hypothesize that the apiose cassette (XpUAS, XpApiT, and XpXylT) encoding genes ended up in this Api operon by horizontal gene transfer. Interestingly, the presence of two IS3 transposases flanking the apiose operon supports a hypothetical transfer event. One transposase is 1568 bp 5’ of the operon and the other is 1792 bp 3’ to the operon.

It will be interesting to figure out in the future (when more genomes are sequenced) the DNA source that was used to insert the operon into the *X*. *pisi* genome: was it from closely associated microbes, plant host, or through a viral vector? Other *Xanthomonas* species are reported to contain evidence of cross-kingdom gene transfer [62]. Due to the wide range of hosts *Xanthomonas* spp. infect, it is reasonable to speculate the purpose of surface apiosides and/or xylosides specific to *X*. *pisi* is to aid in pathogenicity or elude host defenses. Perhaps exhibition of apiose and/or xylose acts to repel other soil-dwelling organisms as *X*. *pisi* struggles to vie for survival in its local microbiome. Continued study of this apiose operon may provide insight into how bacteria use genomic change to adapt to their specific environment.

The identification and characterization of XpApiT as a genuine apiosyltransferase reveals a new activity exclusive to *X*. *pisi*. Perhaps *X*. *pisi* uses a surface apioside as a cloak from host defense or to combat rival microbes. Characterization of XpXyltT generated by this operon demonstrates that it uses a UDP-Xyl substrate generated by the same enzyme (XpUAS) that generates UDP-Api substrate for XpApiT. It is possible that the XpXylT similarly functions to modify surface glycans that impart ecological advantage to *X*. *pisi*. Development of a tractable strain of *X*. *pisi* would enable future knock-out studies to provide biological relevance of apiose and xylose in this microbe.

## Supporting information

S1 Fig GT family 90 multiple sequence alignment. Full amino acid alignment of XpApiT (MH673348) and representative members of GT family 90 from the following organisms: *Arabidopsis thaliana* (Q9SMT6; O80836; Q9SH28; Q56Y51; NP_172202; Q9M2D5; NP_191688; Q0WS79; O80835), *Oryza sativa* Japonica (XP_015636439; Q0DEH1; Q6Z1Y8; XP_015633451), *Vitis vinifera* (CAN66409; CAN70601; CAN65871), *Homo sapiens* (Q6UW63), *Mus musculus* (NP_076134), *Danio rerio* (Q7ZVE6), *Nematostella vectensis* (XP_001639335), *Drosophila melanogaster* (Q9VCU4; NP_651095), *Xenopus laevis* (Q6DDJ2), *Bos taurus* (Q5E9Q1), Invertebrate iridescent virus 6 (NP_149642), *Cryptococcus neoformans* var. *neoformans* JEC21 (XP_567800). Sequences were aligned with PRALINE [63] using the BLOSUM62 scoring matrix.
(PDF)

S2 Fig GT family 2 multiple sequence alignment. Full amino acid alignment of XpXylT (MH673349) and representative members of GT family 2 from the following organisms: *Jannaschia* sp CCS1 (ABD53137; ABD56186; ABD56187; ABD53753; ABD56179), *Dinoroseobacter shibae* (WP 012180235; WP 012179538), *Ruegeria pomeroyi* (AAV93367; AAV93368; AAV94731; AAV94670; AAV95896; AAV95846; AAV94320), *Ruegeria* sp TM1040 (ABF62750; ABF62751; ABF64507), *Paracoccus denitrificans* (WP 011748578), *Rhizobium etli* CFN 42 (ABC92155), *Geodermatophilus obscurus* (WP 012946629), *Nostoc* sp PCC 7120 (BAB73679), *Pseudovibrio* sp FO-BEG1 (WP 014283852), *Ochrobactrum anthropi* (WP 012090710), *Beijerinckia indica* (WP 012385681), *Sinorhizobium fredii* (WP 012708451), *Synechococcus* sp WH 8102 (CAE07032), *Methylobacterium radiotolerans* (WP 012317373), *Porphyromonas asaccharolytica* (WP 004330966), *Gluconobacter oxydans* (AAW60025), *Streptococcus suis* (CYU93668), *Acidiphilium cryptum* (ABQ29611), *Selenomonas sputigena* (WP 006193730), *Thalassiosira pseudonana* (XP 002288249), *Nematostella vectensis* (XP 001635452), *Rattus norvegicus* (NP 001099537), *Gloeobacter violaceus* (BAC90139), *Granulibacter bethesdensis* (ABI61222), *Rhodothermus marinus* (WP 012843638), *Paenibacillus* sp JDR-2 (WP 015843614), *Methylorubrum extorquens* (WP 003600880). Sequences were aligned with PRALINE [63] using the BLOSUM62 scoring matrix.
(PDF)

S1 Table Primers used in plasmid generation. Obtained from IDT.
(DOCX)

## References

[1] Messner P. Prokaryotic glycoproteins: unexplored but important. J Bacteriol. 2004;186(9):2517–9. 10.1128/JB.186.9.2517-2519.2004 15090489 PMC387777

[2] Logan SM. Flagellar glycosylation—a new component of the motility repertoire? Microbiology. 2006;152(Pt 5):1249–62. 10.1099/mic.0.28735-0 16622043

[3] Kay E, Lesk VI, Tamaddoni-Nezhad A, Hitchen PG, Dell A, Sternberg MJ, et al Systems analysis of bacterial glycomes. Biochem Soc Trans. 2010;38(5):1290–3. 10.1042/BST0381290 20863301

[4] Liu J, Mushegian A. Three monophyletic superfamilies account for the majority of the known glycosyltransferases. Protein Sci. 2003;12(7):1418–31. 10.1110/ps.0302103 12824488 PMC2323934

[5] Gloster TM. Advances in understanding glycosyltransferases from a structural perspective. Curr Opin Struct Biol. 2014;28:131–41. 10.1016/j.sbi.2014.08.012 25240227 PMC4330554

[6] Jarrell KF, Ding Y, Meyer BH, Albers SV, Kaminski L, Eichler J. N-linked glycosylation in Archaea: a structural, functional, and genetic analysis. Microbiol Mol Biol Rev. 2014;78(2):304–41. 10.1128/MMBR.00052-13 24847024 PMC4054257

[7] Campbell JA, Davies GJ, Bulone V, Henrissat B. A classification of nucleotide-diphospho-sugar glycosyltransferases based on amino acid sequence similarities. Biochem J. 1997;326 (Pt 3):929–39. 9334165 10.1042/bj3260929u PMC1218753

[8] Coutinho PM, Deleury E, Davies GJ, Henrissat B. An evolving hierarchical family classification for glycosyltransferases. J Mol Biol. 2003;328(2):307–17. 12691742 10.1016/s0022-2836(03)00307-3

[9] Ha S, Walker D, Shi Y, Walker S. The 1.9 A crystal structure of Escherichia coli MurG, a membrane-associated glycosyltransferase involved in peptidoglycan biosynthesis. Protein Sci. 2000;9(6):1045–52. 10.1110/ps.9.6.1045 10892798 PMC2144650

[10] Marrec-Fairley M, Piette A, Gallet X, Brasseur R, Hara H, Fraipont C, et al Differential functionalities of amphiphilic peptide segments of the cell-septation penicillin-binding protein 3 of Escherichia coli. Mol Microbiol. 2000;37(5):1019–31. 10972821 10.1046/j.1365-2958.2000.02054.x

[11] van Heijenoort J. Formation of the glycan chains in the synthesis of bacterial peptidoglycan. Glycobiology. 2001;11(3):25R–36R. 11320055 10.1093/glycob/11.3.25r

[12] Raetz CR. Biochemistry of endotoxins. Annu Rev Biochem. 1990;59:129–70. 10.1146/annurev.bi.59.070190.001021 1695830

[13] Raetz CRH. *Escherichia coli* and *Salmonella*. Niedhardt eF, editor. Washington, DC: Am. Soc. Microbiol.; 1996.

[14] Raetz CR. Bacterial endotoxins: extraordinary lipids that activate eucaryotic signal transduction. J Bacteriol. 1993;175(18):5745–53. 8376321 10.1128/jb.175.18.5745-5753.1993 PMC206651

[15] Wyckoff TJ, Raetz CR, Jackman JE. Antibacterial and anti-inflammatory agents that target endotoxin. Trends Microbiol. 1998;6(4):154–9. 9587193 10.1016/s0966-842x(98)01230-x

[16] Jansson PE, Kenne L, Lindberg B. Structure of extracellular polysaccharide from Xanthomonas campestris. Carbohydr Res. 1975;45:275–82. 1212669 10.1016/s0008-6215(00)85885-1

[17] Melton LD, Mindt L, Rees DA, Sanderson GR. Covalent structure of the extracellular polysaccharide from Xanthomonas campestris: evidence from partial hydrolysis studies. Carbohydr Res. 1976;46(2):245–57. 1260790 10.1016/s0008-6215(00)84296-2

[18] Dow JM, Osbourn AE, Wilson TJ, Daniels MJ. A locus determining pathogenicity of Xanthomonas campestris is involved in lipopolysaccharide biosynthesis. Mol Plant Microbe Interact. 1995;8(5):768–77. 7579621 10.1094/mpmi-8-0768

[19] Braun SG, Meyer A, Holst O, Puhler A, Niehaus K. Characterization of the Xanthomonas campestris pv. campestris lipopolysaccharide substructures essential for elicitation of an oxidative burst in tobacco cells. Mol Plant Microbe Interact. 2005;18(7):674–81. 10.1094/MPMI-18-0674 16042013

[20] Smith JA, Bar-Peled M. Synthesis of UDP-apiose in Bacteria: The marine phototroph *Geminicoccus roseus* and the plant pathogen *Xanthomonas pisi*. PLoS One. 2017;12(9):e0184953 10.1371/journal.pone.0184953 28931093 PMC5607165

[21] Altschul SF, Madden TL, Schaffer AA, Zhang J, Zhang Z, Miller W, et al Gapped BLAST and PSI-BLAST: a new generation of protein database search programs. Nucleic Acids Res. 1997;25(17):3389–402. 9254694 10.1093/nar/25.17.3389 PMC146917

[22] Finn RD, Clements J, Arndt W, Miller BL, Wheeler TJ, Schreiber F, et al HMMER web server: 2015 update. Nucleic Acids Res. 2015;43(W1):W30–8. 10.1093/nar/gkv397 25943547 PMC4489315

[23] Sievers F, Wilm A, Dineen D, Gibson TJ, Karplus K, Li W, et al Fast, scalable generation of high-quality protein multiple sequence alignments using Clustal Omega. Mol Syst Biol. 2011;7:539 10.1038/msb.2011.75 21988835 PMC3261699

[24] McWilliam H, Li W, Uludag M, Squizzato S, Park YM, Buso N, et al Analysis tool web services from the EMBL-EBI. Nucleic Acids Res. 2013;41(Web Server issue):W597–600. 10.1093/nar/gkt376 23671338 PMC3692137

[25] Li W, Cowley A, Uludag M, Gur T, McWilliam H, Squizzato S, et al The EMBL-EBI bioinformatics web and programmatic tools framework. Nucleic Acids Res. 2015;43(W1):W580–4. 10.1093/nar/gkv279 25845596 PMC4489272

[26] Huson DH, Scornavacca C. Dendroscope 3: an interactive tool for rooted phylogenetic trees and networks. Syst Biol. 2012;61(6):1061–7. 10.1093/sysbio/sys062 22780991

[27] Yang T, Bar-Peled L, Gebhart L, Lee SG, Bar-Peled M. Identification of galacturonic acid-1-phosphate kinase, a new member of the GHMP kinase superfamily in plants, and comparison with galactose-1-phosphate kinase. J Biol Chem. 2009;284(32):21526–35. 10.1074/jbc.M109.014761 19509290 PMC2755877

[28] Guyett P, Glushka J, Gu X, Bar-Peled M. Real-time NMR monitoring of intermediates and labile products of the bifunctional enzyme UDP-apiose/UDP-xylose synthase. Carbohydr Res. 2009;344(9):1072–8. 10.1016/j.carres.2009.03.026 19375693 PMC4000172

[29] Smith J, Yang Y., Levy S., Adelusi O. O., Hahn M. G., O'Neill M. A., & Bar-Peled M. Functional Characterization of UDP-apiose Synthases from Bryophytes and Green Algae Provides Insight into the Appearance of Apiose-containing Glycans during Plant Evolution. J Biol Chem. 2016;291(41):21434–47. 10.1074/jbc.M116.749069 27551039 PMC5076816

[30] Broach B, Gu X, Bar-Peled M. Biosynthesis of UDP-glucuronic acid and UDP-galacturonic acid in *Bacillus cereus* subsp. cytotoxis NVH 391–98. FEBS J. 2012;279(1):100–12. 10.1111/j.1742-4658.2011.08402.x 22023070 PMC3240692

[31] Leemhuis H, Kragh KM, Dijkstra BW, Dijkhuizen L. Engineering cyclodextrin glycosyltransferase into a starch hydrolase with a high exo-specificity. J Biotechnol. 2003;103(3):203–12. 12890607 10.1016/s0168-1656(03)00126-3

[32] Sindhuwinata N, Munoz E, Munoz FJ, Palcic MM, Peters H, Peters T. Binding of an acceptor substrate analog enhances the enzymatic activity of human blood group B galactosyltransferase. Glycobiology. 2010;20(6):718–23. 10.1093/glycob/cwq019 20154292

[33] Brockhausen I. Crossroads between Bacterial and Mammalian Glycosyltransferases. Front Immunol. 2014;5:492 10.3389/fimmu.2014.00492 25368613 PMC4202792

[34] Sheikh MO, Halmo SM, Patel S, Middleton D, Takeuchi H, Schafer CM, et al Rapid screening of sugar-nucleotide donor specificities of putative glycosyltransferases. Glycobiology. 2017;27(3):206–12. 10.1093/glycob/cww114 28177478 PMC5789813

[35] Goto M, Okabe N. Cellulolytic activity of phytopathogenic bacteria. Nature. 1958;182(4648):1516. 10.1038/1821516a0 13613322

[36] Vauterin L, Hoste B, Kersters K, Swings J. Reclassification of *Xanthomonas*. Int J Syst Bacteriol. 1995;45:472–89.

[37] Li Z, Hwang S, Bar-Peled M. Discovery of a Unique Extracellular Polysaccharide in Members of the Pathogenic Bacillus That Can Co-form with Spores. J Biol Chem. 2016;291(36):19051–67. 10.1074/jbc.M116.724708 27402849 PMC5009276

[38] Yang T, Bar-Peled Y, Smith JA, Glushka J, Bar-Peled M. In-microbe formation of nucleotide sugars in engineered Escherichia coli. Anal Biochem. 2012;421(2):691–8. 10.1016/j.ab.2011.12.028 22244806

[39] Hwang S, Li Z, Bar-Peled Y, Aronov A, Ericson J, Bar-Peled M. The biosynthesis of UDP-D-FucNAc-4N-(2)-oxoglutarate (UDP-Yelosamine) in *Bacillus cereus* ATCC 14579: Pat and Pyl, an aminotransferase and an ATP-dependent Grasp protein that ligates 2-oxoglutarate to UDP-4-amino-sugars. J Biol Chem. 2014;289(51):35620–32. 10.1074/jbc.M114.614917 25368324 PMC4271244

[40] Bradford MM. A rapid and sensitive method for the quantitation of microgram quantities of protein utilizing the principle of protein-dye binding. Anal Biochem. 1976;72:248–54. 942051 10.1016/0003-2697(76)90527-3

[41] Rezanka T, Nedbalova L, Kolouchova I, Sigler K. LC-MS/APCI identification of glucoside esters and diesters of astaxanthin from the snow alga Chlamydomonas nivalis including their optical stereoisomers. Phytochemistry. 2013;88:34–42. 10.1016/j.phytochem.2013.01.003 23398889

[42] Castle SA, Owuor EA, Thompson SH, Garnsey MR, Klutts JS, Doering TL, et al Beta1,2-xylosyltransferase Cxt1p is solely responsible for xylose incorporation into Cryptococcus neoformans glycosphingolipids. Eukaryot Cell. 2008;7(9):1611–5. 10.1128/EC.00458-07 18676952 PMC2547070

[43] Klutts JS, Levery SB, Doering TL. A beta-1,2-xylosyltransferase from Cryptococcus neoformans defines a new family of glycosyltransferases. J Biol Chem. 2007;282(24):17890–9. 10.1074/jbc.M701941200 17430900

[44] Klutts JS, Doering TL. Cryptococcal xylosyltransferase 1 (Cxt1p) from Cryptococcus neoformans plays a direct role in the synthesis of capsule polysaccharides. J Biol Chem. 2008;283(21):14327–34. 10.1074/jbc.M708927200 18347023 PMC2386922

[45] Chang YC, Kwon-Chung KJ. Isolation, characterization, and localization of a capsule-associated gene, CAP10, of Cryptococcus neoformans. J Bacteriol. 1999;181(18):5636–43. 10482503 10.1128/jb.181.18.5636-5643.1999 PMC94082

[46] Marchler-Bauer A, Bo Y, Han L, He J, Lanczycki CJ, Lu S, et al CDD/SPARCLE: functional classification of proteins via subfamily domain architectures. Nucleic Acids Res. 2017;45(D1):D200–D3. 10.1093/nar/gkw1129 27899674 PMC5210587

[47] Acar M, Jafar-Nejad H, Takeuchi H, Rajan A, Ibrani D, Rana NA, et al Rumi is a CAP10 domain glycosyltransferase that modifies Notch and is required for Notch signaling. Cell. 2008;132(2):247–58. 10.1016/j.cell.2007.12.016 18243100 PMC2275919

[48] Takeuchi H, Fernandez-Valdivia RC, Caswell DS, Nita-Lazar A, Rana NA, Garner TP, et al Rumi functions as both a protein O-glucosyltransferase and a protein O-xylosyltransferase. Proc Natl Acad Sci U S A. 2011;108(40):16600–5. 10.1073/pnas.1109696108 21949356 PMC3189016

[49] Teng Y, Liu Q, Ma J, Liu F, Han Z, Wang Y, et al Cloning, expression and characterization of a novel human CAP10-like gene hCLP46 from CD34(+) stem/progenitor cells. Gene. 2006;371(1):7–15. 10.1016/j.gene.2005.08.027 16524674

[50] Fernandez-Valdivia R, Takeuchi H, Samarghandi A, Lopez M, Leonardi J, Haltiwanger RS, et al Regulation of mammalian Notch signaling and embryonic development by the protein O-glucosyltransferase Rumi. Development. 2011;138(10):1925–34. 10.1242/dev.060020 21490058 PMC3082299

[51] Ramkumar N, Harvey BM, Lee JD, Alcorn HL, Silva-Gagliardi NF, McGlade CJ, et al Protein O-Glucosyltransferase 1 (POGLUT1) Promotes Mouse Gastrulation through Modification of the Apical Polarity Protein CRUMBS2. PLoS Genet. 2015;11(10):e1005551 10.1371/journal.pgen.1005551 26496195 PMC4619674

[52] Hart DA, Kindel PK. Isolation and partial characterization of apiogalacturonans from the cell wall of Lemna minor. Biochem J. 1970;116(4):569–79. 4314131 10.1042/bj1160569 PMC1185401

[53] Ortmann R, Sutter A, Grisebach H. Purification and properties of udpapiose: 7-O- (-D-glucosyl)-flavone apiosyltransferase from cell suspension cultures of parsley. Biochim Biophys Acta. 1972;289(2):293–302. 4650134 10.1016/0005-2744(72)90080-0

[54] Somerville C. Cellulose synthesis in higher plants. Annu Rev Cell Dev Biol. 2006;22:53–78. 10.1146/annurev.cellbio.22.022206.160206 16824006

[55] Kanipes MI, Tan X, Akelaitis A, Li J, Rockabrand D, Guerry P, et al Genetic analysis of lipooligosaccharide core biosynthesis in Campylobacter jejuni 81–176. J Bacteriol. 2008;190(5):1568–74. 10.1128/JB.01696-07 18156268 PMC2258656

[56] Kahler CM, Carlson RW, Rahman MM, Martin LE, Stephens DS. Two glycosyltransferase genes, lgtF and rfaK, constitute the lipooligosaccharide ice (inner core extension) biosynthesis operon of Neisseria meningitidis. J Bacteriol. 1996;178(23):6677–84. 8955282 10.1128/jb.178.23.6677-6684.1996 PMC178561

[57] Kapitonov D, Yu RK. Conserved domains of glycosyltransferases. Glycobiology. 1999;9(10):961–78. 10521532 10.1093/glycob/9.10.961

[58] Gagneux P, Varki A. Evolutionary considerations in relating oligosaccharide diversity to biological function. Glycobiology. 1999;9(8):747–55. 10406840 10.1093/glycob/9.8.747

[59] Breton C, Imberty A. Structure/function studies of glycosyltransferases. Curr Opin Struct Biol. 1999;9(5):563–71. 10508766 10.1016/s0959-440x(99)00006-8

[60] Stevenson G, Andrianopoulos K, Hobbs M, Reeves PR. Organization of the Escherichia coli K-12 gene cluster responsible for production of the extracellular polysaccharide colanic acid. J Bacteriol. 1996;178(16):4885–93. 8759852 10.1128/jb.178.16.4885-4893.1996 PMC178271

[61] Bansal K, Midha S, Kumar S, Patil PB. Ecological and Evolutionary Insights into Xanthomonas citri Pathovar Diversity. Appl Environ Microbiol. 2017;83(9). 10.1128/AEM.02993-16 PMC5394309 28258140

[62] Gardiner DM, Upadhyaya NM, Stiller J, Ellis JG, Dodds PN, Kazan K, et al Genomic analysis of Xanthomonas translucens pathogenic on wheat and barley reveals cross-kingdom gene transfer events and diverse protein delivery systems. PLoS One. 2014;9(1):e84995 10.1371/journal.pone.0084995 24416331 PMC3887016

[63] Simossis VA, Heringa J. PRALINE: a multiple sequence alignment toolbox that integrates homology-extended and secondary structure information. Nucleic Acids Res. 2005;33(Web Server issue):W289–94. 10.1093/nar/gki390 15980472 PMC1160151

