# Supplementary material for: Identification of an apiosyltransferase in the plant pathogen Xanthomonas pisi
Source: PLoS One. 2018 Oct 18;13(10):e0206187. doi: 10.1371/journal.pone.0206187 (PMC6193724; doi:10.1371/journal.pone.0206187)
Supplement: S1 Fig — Full amino acid alignment of XpApiT (MH673348) and representative members of GT family 90 from the following organisms: Arabidopsis thaliana (Q9SMT6; O80836; Q9SH28; Q56Y51; NP_172202; Q9M2D5; NP_191688; Q0WS79; O80835), Oryza sativa Japonica (XP_015636439; Q0DEH1; Q6Z1Y8; XP_015633451), Vitis vinifera (CAN66409; CAN70601; CAN65871), Homo sapiens (Q6UW63), Mus musculus (NP_076134), Danio rerio (Q7ZVE6), Nematostella vectensis (XP_001639335), Drosophila melanogaster (Q9VCU4; NP_651095), Xenopus laevis (Q6DDJ2), Bos taurus (Q5E9Q1), Invertebrate iridescent virus 6 (NP_149642), Cryptococcus neoformans var. neoformans JEC21 (XP_567800). Sequences were aligned with PRALINE [63] using the BLOSUM62 scoring matrix. (PDF) [file pone.0206187.s001.pdf]

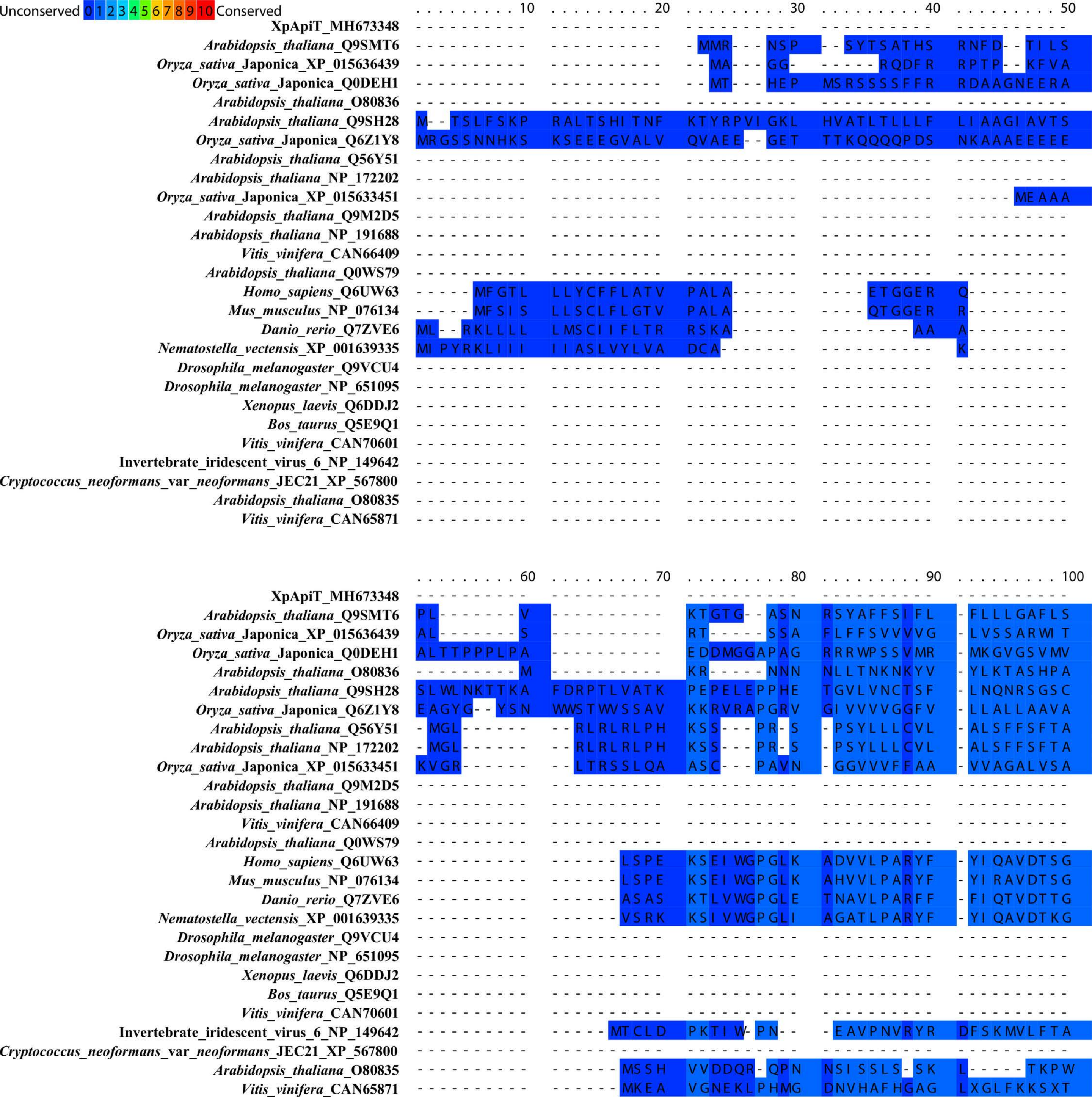

Unconserved 0 1 2 3 4 5 6 7 8 9 10 Conserved
10
20
30
40
50
60
70
80
90
100
XpApiT_MH673348
Arabidopsis_thaliana_Q9SMT6
Oryza_sativa_Japonica_XP_015636439
Oryza_sativa_Japonica_Q0DEH1
Arabidopsis_thaliana_O80836
Arabidopsis_thaliana_Q9SH28
Oryza_sativa_Japonica_Q6Z1Y8
Arabidopsis_thaliana_Q56Y51
Arabidopsis_thaliana_NP_172202
Oryza_sativa_Japonica_XP_015633451
Arabidopsis_thaliana_Q9M2D5
Arabidopsis_thaliana_NP_191688
Vitis_vinifera_CAN66409
Arabidopsis_thaliana_Q0WS79
Homo_sapiens_Q6UW63
Mus_musculus_NP_076134
Danio_rerio_Q7ZVE6
Nematostella_vectensis_XP_001639335
Drosophila_melanogaster_Q9VCU4
Drosophila_melanogaster_NP_651095
Xenopus_laevis_Q6DDJ2
Bos_taurus_Q5E9Q1
Vitis_vinifera_CAN70601
Invertebrate_iridescent_virus_6_NP_149642
Cryptococcus_neoformans_var_neoformans_JEC21_XP_567800
Arabidopsis_thaliana_O80835
Vitis_vinifera_CAN65871

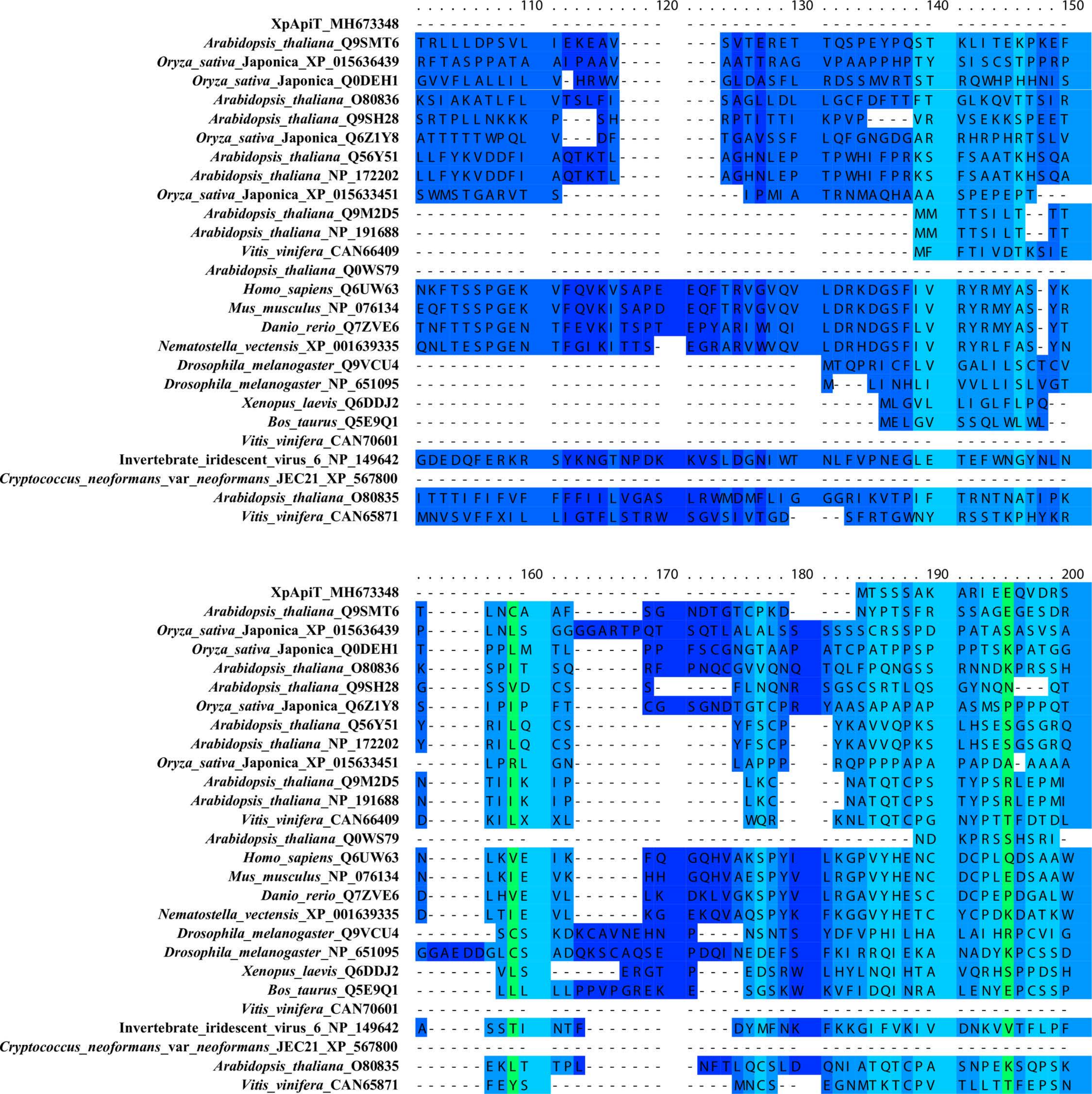
110
120
130
140
150
XpApiT_MH673348
Arabidopsis_thaliana_Q9SMT6
Oryza_sativa_Japonica_XP_015636439
Oryza_sativa_Japonica_Q0DEH1
Arabidopsis_thaliana_O80836
Arabidopsis_thaliana_Q9SH28
Oryza_sativa_Japonica_Q6Z1Y8
Arabidopsis_thaliana_Q56Y51
Arabidopsis_thaliana_NP_172202
Oryza_sativa_Japonica_XP_015633451
Arabidopsis_thaliana_Q9M2D5
Arabidopsis_thaliana_NP_191688
Vitis_vinifera_CAN66409
Arabidopsis_thaliana_Q0WS79
Homo_sapiens_Q6UW63
Mus_musculus_NP_076134
Danio_rerio_Q7ZVE6
Nematostella_vectensis_XP_001639335
Drosophila_melanogaster_Q9VCU4
Drosophila_melanogaster_NP_651095
Xenopus_laevis_Q6DDJ2
Bos_taurus_Q5E9Q1
Vitis_vinifera_CAN70601
Invertebrate_iridescent_virus_6_NP_149642
Cryptococcus_neoformans_var_neoformans_JEC21_XP_567800
Arabidopsis_thaliana_O80835
Vitis_vinifera_CAN65871
160
170
180
190
200

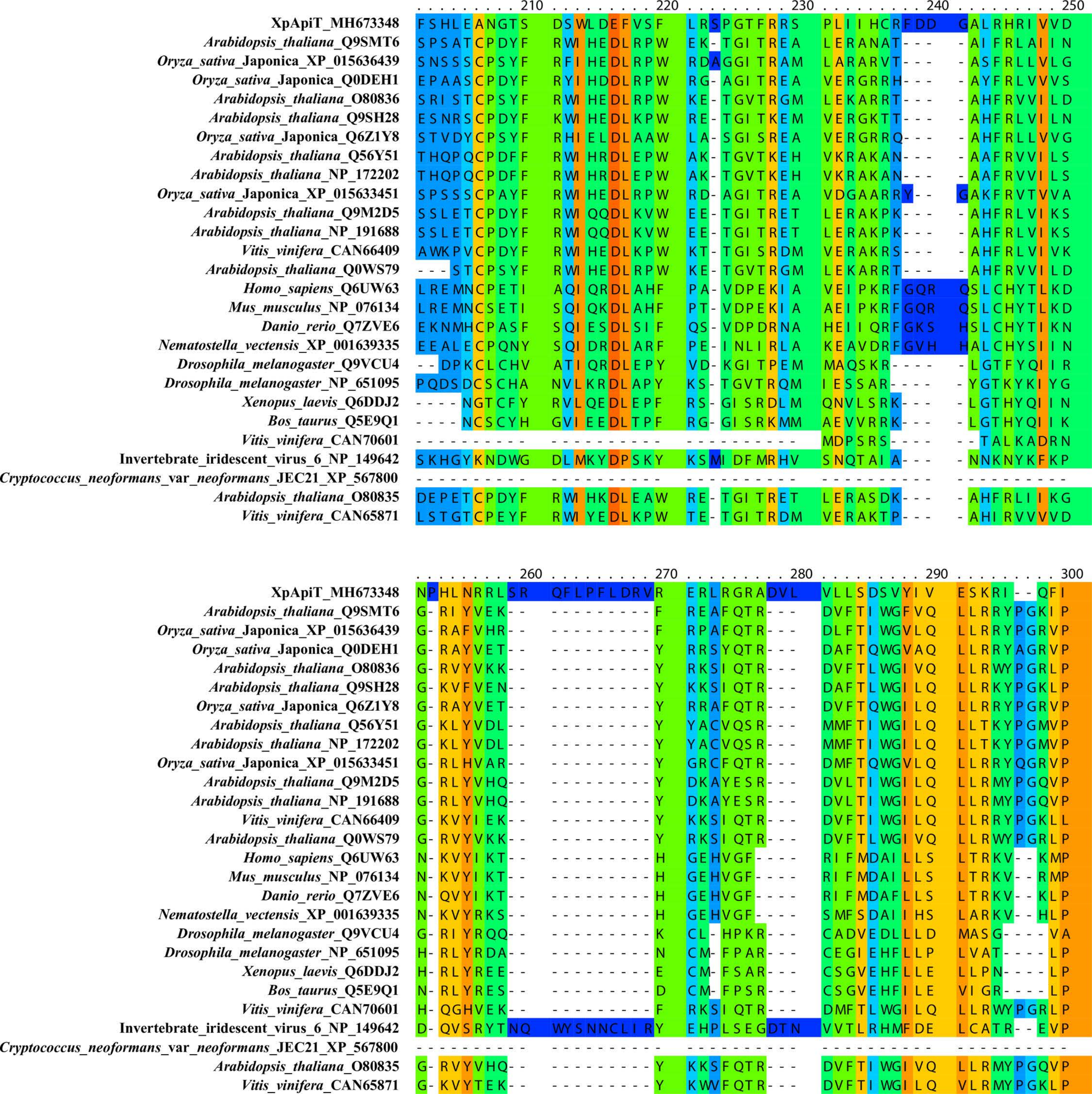

210
220
230
240
250
260
270
280
290
300
XpApiT_MH673348
Arabidopsis_thaliana_Q9SMT6
Oryza_sativa_Japonica_XP_015636439
Oryza_sativa_Japonica_Q0DEH1
Arabidopsis_thaliana_O80836
Arabidopsis_thaliana_Q9SH28
Oryza_sativa_Japonica_Q6Z1Y8
Arabidopsis_thaliana_Q56Y51
Arabidopsis_thaliana_NP_172202
Oryza_sativa_Japonica_XP_015633451
Arabidopsis_thaliana_Q9M2D5
Arabidopsis_thaliana_NP_191688
Vitis_vinifera_CAN66409
Arabidopsis_thaliana_Q0WS79
Homo_sapiens_Q6UW63
Mus_musculus_NP_076134
Danio_rerio_Q7ZVE6
Nematostella_vectensis_XP_001639335
Drosophila_melanogaster_Q9VCU4
Drosophila_melanogaster_NP_651095
Xenopus_laevis_Q6DDJ2
Bos_taurus_Q5E9Q1
Vitis_vinifera_CAN70601
Invertebrate_iridescent_virus_6_NP_149642
Cryptococcus_neoformans_var_neoformans_JEC21_XP_567800
Arabidopsis_thaliana_O80835
Vitis_vinifera_CAN65871

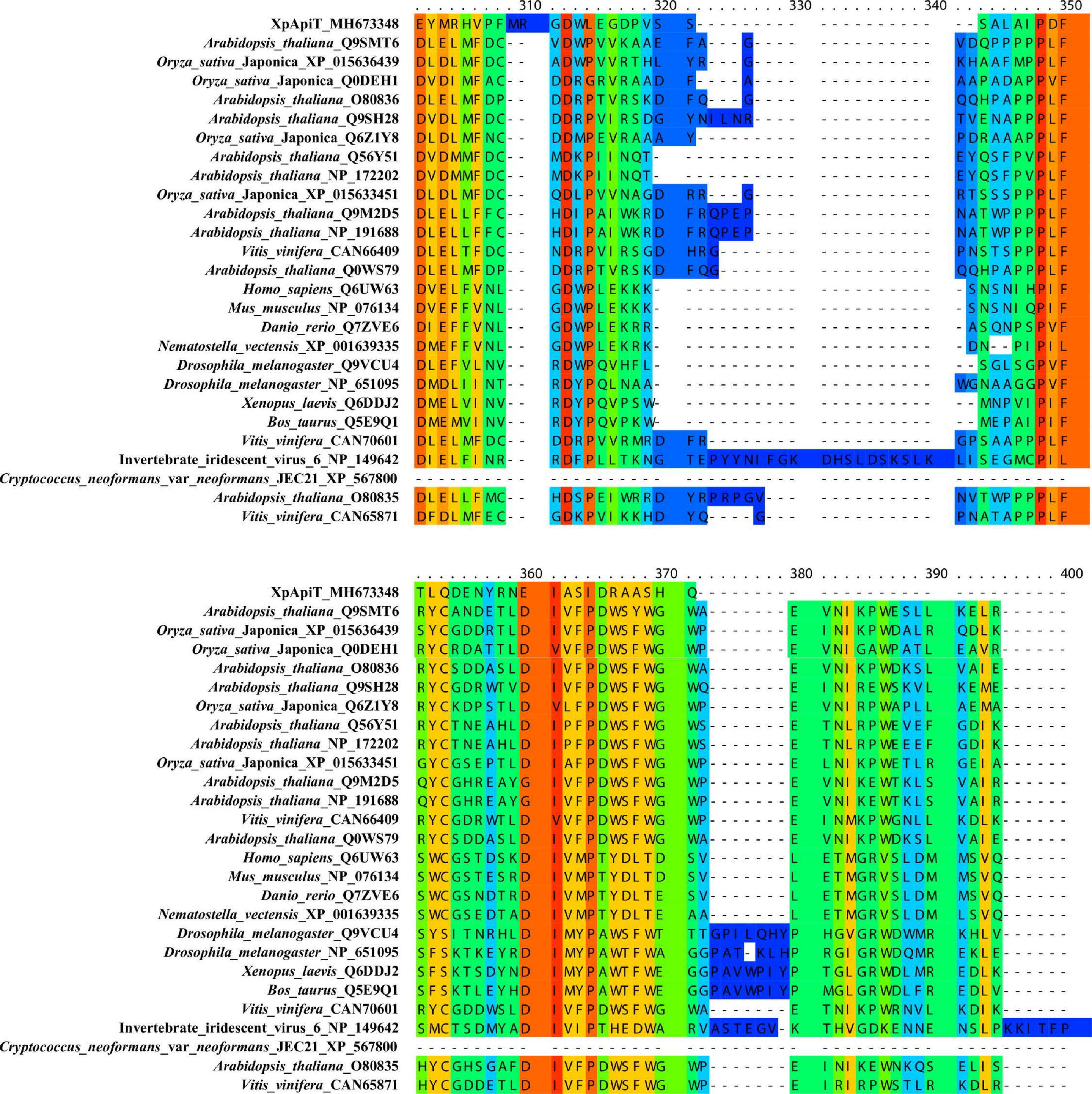
310
320
330
340
350
XpApiT_MH673348
Arabidopsis_thaliana_Q9SMT6
Oryza_sativa_Japonica_XP_015636439
Oryza_sativa_Japonica_Q0DEH1
Arabidopsis_thaliana_O80836
Arabidopsis_thaliana_Q9SH28
Oryza_sativa_Japonica_Q6Z1Y8
Arabidopsis_thaliana_Q56Y51
Arabidopsis_thaliana_NP_172202
Oryza_sativa_Japonica_XP_015633451
Arabidopsis_thaliana_Q9M2D5
Arabidopsis_thaliana_NP_191688
Vitis_vinifera_CAN66409
Arabidopsis_thaliana_Q0WS79
Homo_sapiens_Q6UW63
Mus_musculus_NP_076134
Danio_rerio_Q7ZVE6
Nematostella_vectensis_XP_001639335
Drosophila_melanogaster_Q9VCU4
Drosophila_melanogaster_NP_651095
Xenopus_laevis_Q6DDJ2
Bos_taurus_Q5E9Q1
Vitis_vinifera_CAN70601
Invertebrate_iridescent_virus_6_NP_149642
Cryptococcus_neoformans_var_neoformans_JEC21_XP_567800
Arabidopsis_thaliana_O80835
Vitis_vinifera_CAN65871
360
370
380
390
400

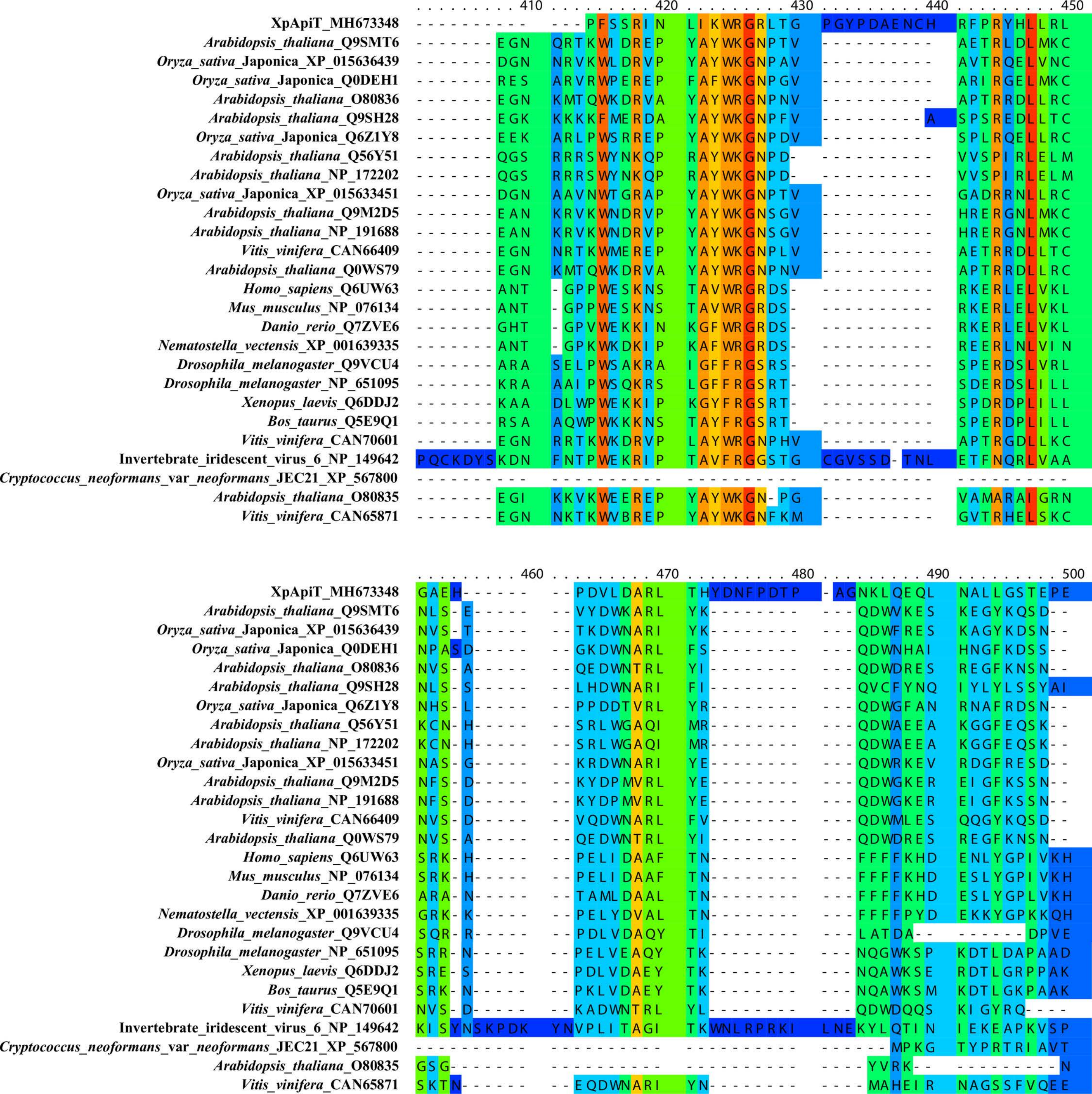
410
420
430
440
450
460
470
480
490
500
XpApiT_MH673348
Arabidopsis_thaliana_Q9SMT6
Oryza_sativa_Japonica_XP_015636439
Oryza_sativa_Japonica_Q0DEH1
Arabidopsis_thaliana_O80836
Arabidopsis_thaliana_Q9SH28
Oryza_sativa_Japonica_Q6Z1Y8
Arabidopsis_thaliana_Q56Y51
Arabidopsis_thaliana_NP_172202
Oryza_sativa_Japonica_XP_015633451
Arabidopsis_thaliana_Q9M2D5
Arabidopsis_thaliana_NP_191688
Vitis_vinifera_CAN66409
Arabidopsis_thaliana_Q0WS79
Homo_sapiens_Q6UW63
Mus_musculus_NP_076134
Danio_rerio_Q7ZVE6
Nematostella_vectensis_XP_001639335
Drosophila_melanogaster_Q9VCU4
Drosophila_melanogaster_NP_651095
Xenopus_laevis_Q6DDJ2
Bos_taurus_Q5E9Q1
Vitis_vinifera_CAN70601
Invertebrate_iridescent_virus_6_NP_149642
Cryptococcus_neoformans_var_neoformans_JEC21_XP_567800
Arabidopsis_thaliana_O80835
Vitis_vinifera_CAN65871

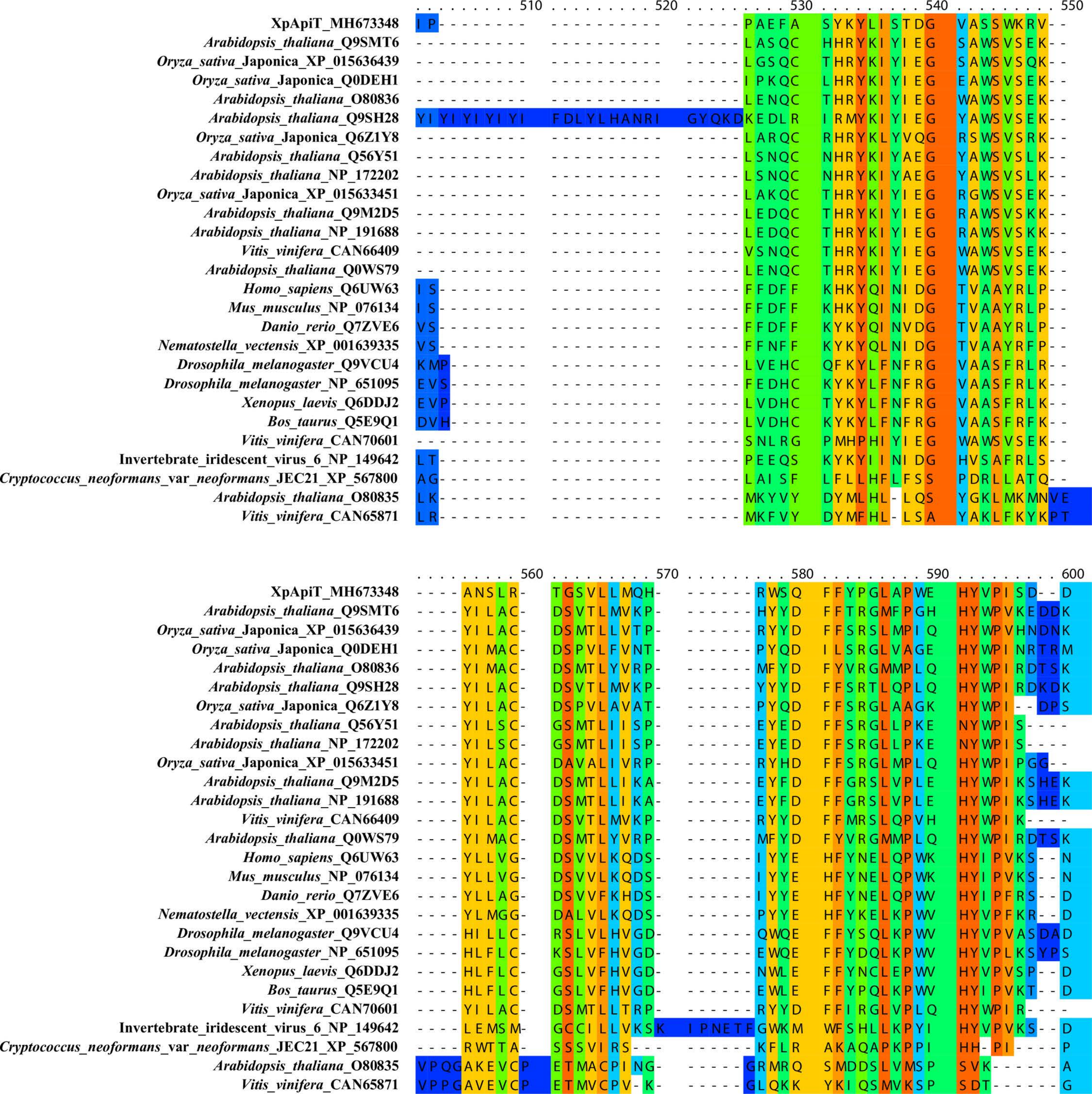

510
520
530
540
550
560
570
580
590
600
XpApiT_MH673348
Arabidopsis_thaliana_Q9SMT6
Oryza_sativa_Japonica_XP_015636439
Oryza_sativa_Japonica_Q0DEH1
Arabidopsis_thaliana_O80836
Arabidopsis_thaliana_Q9SH28
Oryza_sativa_Japonica_Q6Z1Y8
Arabidopsis_thaliana_Q56Y51
Arabidopsis_thaliana_NP_172202
Oryza_sativa_Japonica_XP_015633451
Arabidopsis_thaliana_Q9M2D5
Arabidopsis_thaliana_NP_191688
Vitis_vinifera_CAN66409
Arabidopsis_thaliana_Q0WS79
Homo_sapiens_Q6UW63
Mus_musculus_NP_076134
Danio_rerio_Q7ZVE6
Nematostella_vectensis_XP_001639335
Drosophila_melanogaster_Q9VCU4
Drosophila_melanogaster_NP_651095
Xenopus_laevis_Q6DDJ2
Bos_taurus_Q5E9Q1
Vitis_vinifera_CAN70601
Invertebrate_iridescent_virus_6_NP_149642
Cryptococcus_neoformans_var_neoformans_JEC21_XP_567800
Arabidopsis_thaliana_O80835
Vitis_vinifera_CAN65871

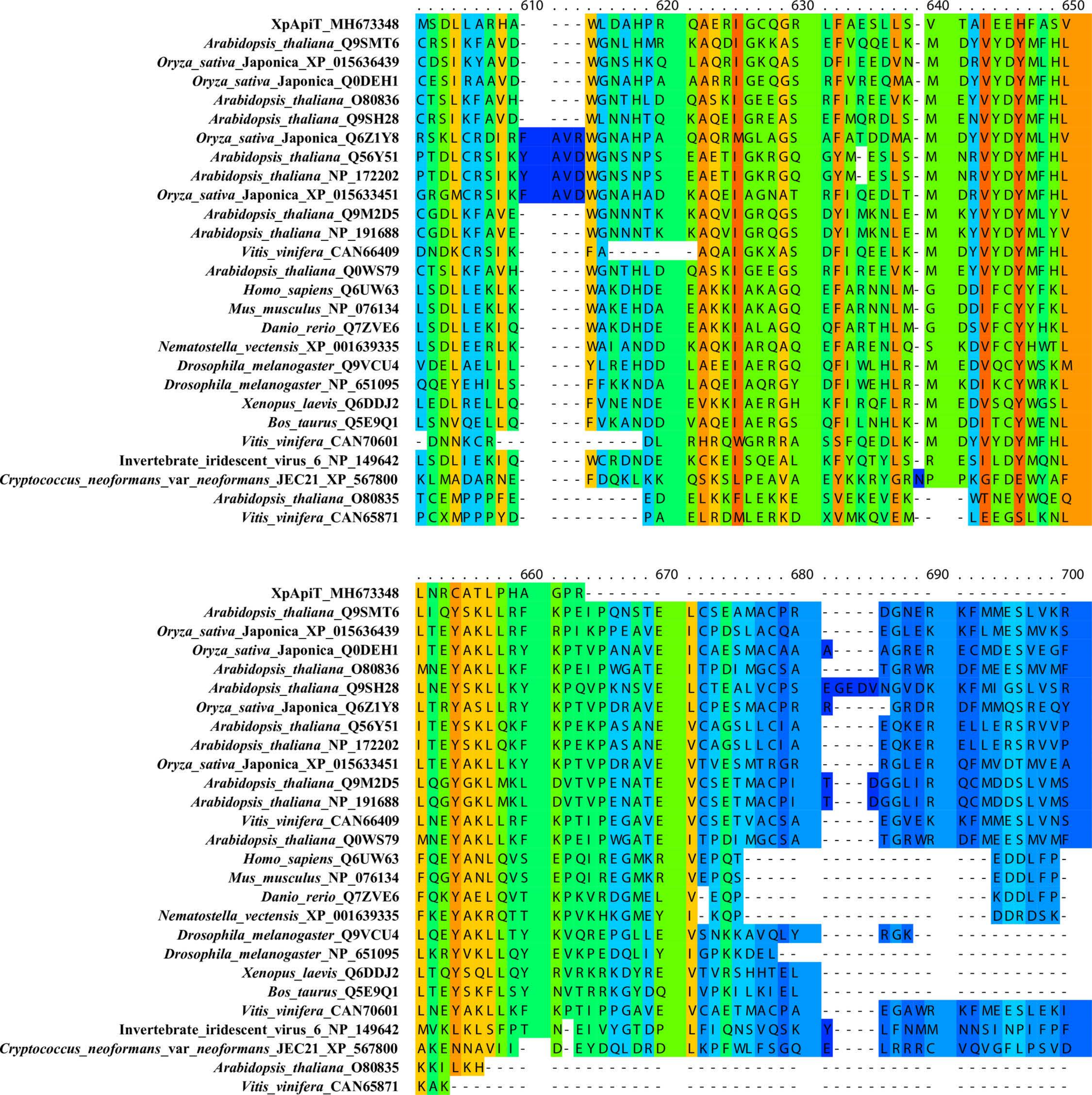
610
620
630
640
650
XpApiT_MH673348
Arabidopsis_thaliana_Q9SMT6
Oryza_sativa_Japonica_XP_015636439
Oryza_sativa_Japonica_Q0DEH1
Arabidopsis_thaliana_O80836
Arabidopsis_thaliana_Q9SH28
Oryza_sativa_Japonica_Q6Z1Y8
Arabidopsis_thaliana_Q56Y51
Arabidopsis_thaliana_NP_172202
Oryza_sativa_Japonica_XP_015633451
Arabidopsis_thaliana_Q9M2D5
Arabidopsis_thaliana_NP_191688
Vitis_vinifera_CAN66409
Arabidopsis_thaliana_Q0WS79
Homo_sapiens_Q6UW63
Mus_musculus_NP_076134
Danio_rerio_Q7ZVE6
Nematostella_vectensis_XP_001639335
Drosophila_melanogaster_Q9VCU4
Drosophila_melanogaster_NP_651095
Xenopus_laevis_Q6DDJ2
Bos_taurus_Q5E9Q1
Vitis_vinifera_CAN70601
Invertebrate_iridescent_virus_6_NP_149642
Cryptococcus_neoformans_var_neoformans_JEC21_XP_567800
Arabidopsis_thaliana_O80835
Vitis_vinifera_CAN65871
660
670
680
690
700

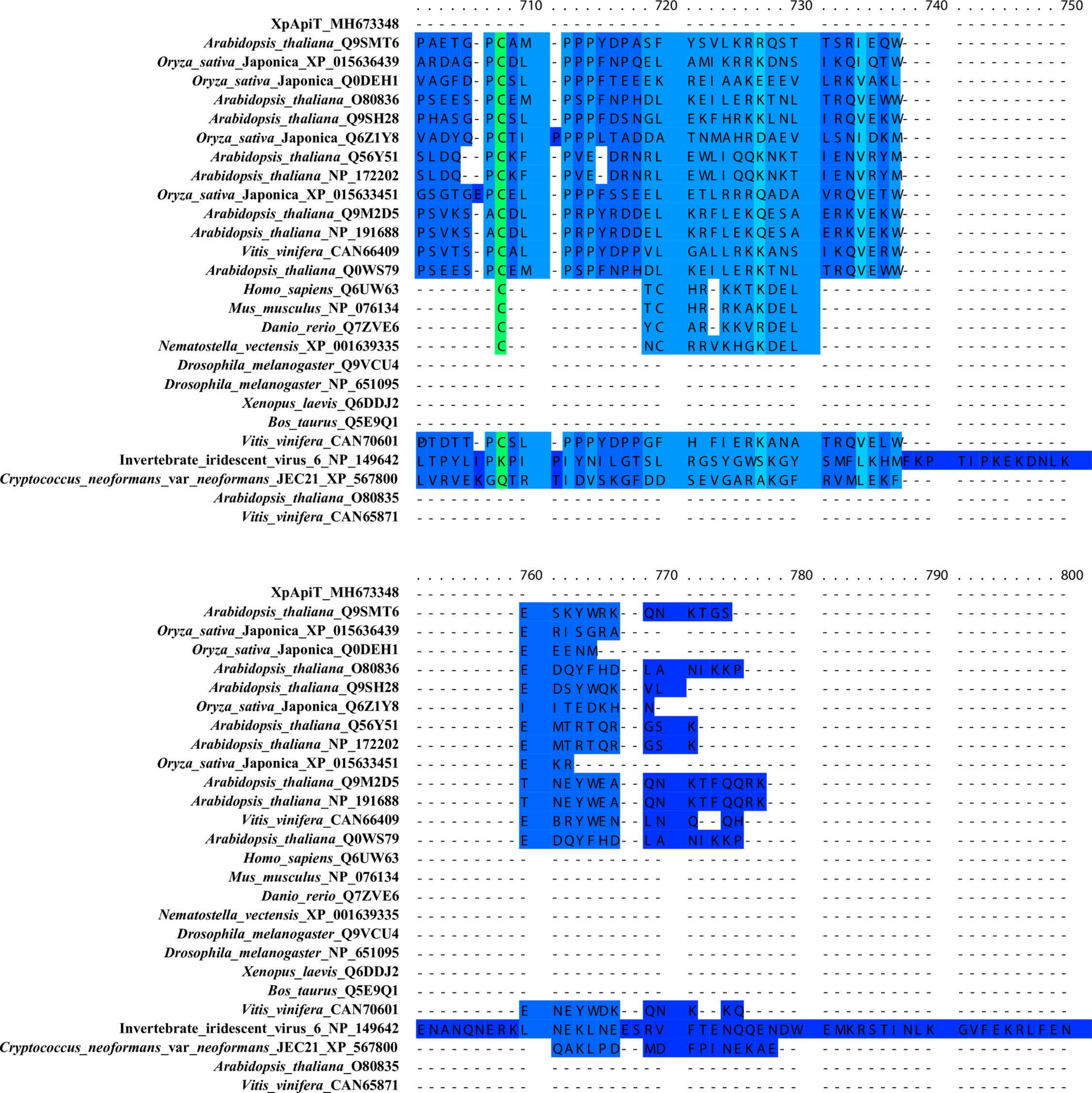

710
720
730
740
750
XpApiT_MH673348
Arabidopsis_thaliana_Q9SMT6
Oryza_sativa_Japonica_XP_015636439
Oryza_sativa_Japonica_Q0DEH1
Arabidopsis_thaliana_O80836
Arabidopsis_thaliana_Q9SH28
Oryza_sativa_Japonica_Q6Z1Y8
Arabidopsis_thaliana_Q56Y51
Arabidopsis_thaliana_NP_172202
Oryza_sativa_Japonica_XP_015633451
Arabidopsis_thaliana_Q9M2D5
Arabidopsis_thaliana_NP_191688
Vitis_vinifera_CAN66409
Arabidopsis_thaliana_Q0WS79
Homo_sapiens_Q6UW63
Mus_musculus_NP_076134
Danio_rerio_Q7ZVE6
Nematostella_vectensis_XP_001639335
Drosophila_melanogaster_Q9VCU4
Drosophila_melanogaster_NP_651095
Xenopus_laevis_Q6DDJ2
Bos_taurus_Q5E9Q1
Vitis_vinifera_CAN70601
Invertebrate_iridescent_virus_6_NP_149642
Cryptococcus_neoformans_var_neoformans_JEC21_XP_567800
Arabidopsis_thaliana_O80835
Vitis_vinifera_CAN65871
760
770
780
790
800
XpApiT_MH673348
Arabidopsis_thaliana_Q9SMT6
Oryza_sativa_Japonica_XP_015636439
Oryza_sativa_Japonica_Q0DEH1
Arabidopsis_thaliana_O80836
Arabidopsis_thaliana_Q9SH28
Oryza_sativa_Japonica_Q6Z1Y8
Arabidopsis_thaliana_Q56Y51
Arabidopsis_thaliana_NP_172202
Oryza_sativa_Japonica_XP_015633451
Arabidopsis_thaliana_Q9M2D5
Arabidopsis_thaliana_NP_191688
Vitis_vinifera_CAN66409
Arabidopsis_thaliana_Q0WS79
Homo_sapiens_Q6UW63
Mus_musculus_NP_076134
Danio_rerio_Q7ZVE6
Nematostella_vectensis_XP_001639335
Drosophila_melanogaster_Q9VCU4
Drosophila_melanogaster_NP_651095
Xenopus_laevis_Q6DDJ2
Bos_taurus_Q5E9Q1
Vitis_vinifera_CAN70601
Invertebrate_iridescent_virus_6_NP_149642
Cryptococcus_neoformans_var_neoformans_JEC21_XP_567800
Arabidopsis_thaliana_O80835
Vitis_vinifera_CAN65871

	810	820	830	840	850
XpApiT_MH673348	----------	----------	----------	----------	----------
*Arabidopsis_thaliana*_Q9SMT6	----------	----------	----------	----------	----------
*Oryza_sativa*_Japonica_XP_015636439	----------	----------	----------	----------	----------
*Oryza_sativa*_Japonica_Q0DEH1	----------	----------	----------	----------	----------
*Arabidopsis_thaliana*_O80836	----------	----------	----------	----------	----------
*Arabidopsis_thaliana*_Q9SH28	----------	----------	----------	----------	----------
*Oryza_sativa*_Japonica_Q6Z1Y8	----------	----------	----------	----------	----------
*Arabidopsis_thaliana*_Q56Y51	----------	----------	----------	----------	----------
*Arabidopsis_thaliana*_NP_172202	----------	----------	----------	----------	----------
*Oryza_sativa*_Japonica_XP_015633451	----------	----------	----------	----------	----------
*Arabidopsis_thaliana*_Q9M2D5	----------	----------	----------	----------	----------
*Arabidopsis_thaliana*_NP_191688	----------	----------	----------	----------	----------
*Vitis_vinifera*_CAN66409	----------	----------	----------	----------	----------
*Arabidopsis_thaliana*_Q0WS79	----------	----------	----------	----------	----------
*Homo_sapiens*_Q6UW63	----------	----------	----------	----------	----------
*Mus_musculus*_NP_076134	----------	----------	----------	----------	----------
*Danio_rerio*_Q7ZVE6	----------	----------	----------	----------	----------
*Nematostella_vectensis*_XP_001639335	----------	----------	----------	----------	----------
*Drosophila_melanogaster*_Q9VCU4	----------	----------	----------	----------	----------
*Drosophila_melanogaster*_NP_651095	----------	----------	----------	----------	----------
*Xenopus_laevis*_Q6DDJ2	----------	----------	----------	----------	----------
*Bos_taurus*_Q5E9Q1	----------	----------	----------	----------	----------
*Vitis_vinifera*_CAN70601	----------	----------	----------	----------	----------
Invertebrate_iridescent_virus_6_NP_149642	KKSTVDLYSI	SGSNVVKKST	SDAQGMIEHI	NEAFISDTCI	NNLLKIIPNF
*Cryptococcus_neoformans*_var_*neoformans*_JEC21_XP_567800	--GRILV---	----------	------PWEE	NLYSNLTADS	SLGIEHVLGG
*Arabidopsis_thaliana*_O80835	----------	----------	----------	----------	----------
*Vitis_vinifera*_CAN65871	----------	----------	----------	----------	----------

	860	870	880	890	900
XpApiT_MH673348	----------	----------	----------	----------	----------
*Arabidopsis_thaliana*_Q9SMT6	----------	----------	----------	----------	----------
*Oryza_sativa*_Japonica_XP_015636439	----------	----------	----------	----------	----------
*Oryza_sativa*_Japonica_Q0DEH1	----------	----------	----------	----------	----------
*Arabidopsis_thaliana*_O80836	----------	----------	----------	----------	----------
*Arabidopsis_thaliana*_Q9SH28	----------	----------	----------	----------	----------
*Oryza_sativa*_Japonica_Q6Z1Y8	----------	----------	----------	----------	----------
*Arabidopsis_thaliana*_Q56Y51	----------	----------	----------	----------	----------
*Arabidopsis_thaliana*_NP_172202	----------	----------	----------	----------	----------
*Oryza_sativa*_Japonica_XP_015633451	----------	----------	----------	----------	----------
*Arabidopsis_thaliana*_Q9M2D5	----------	----------	----------	----------	----------
*Arabidopsis_thaliana*_NP_191688	----------	----------	----------	----------	----------
*Vitis_vinifera*_CAN66409	----------	----------	----------	----------	----------
*Arabidopsis_thaliana*_Q0WS79	----------	----------	----------	----------	----------
*Homo_sapiens*_Q6UW63	----------	----------	----------	----------	----------
*Mus_musculus*_NP_076134	----------	----------	----------	----------	----------
*Danio_rerio*_Q7ZVE6	----------	----------	----------	----------	----------
*Nematostella_vectensis*_XP_001639335	----------	----------	----------	----------	----------
*Drosophila_melanogaster*_Q9VCU4	----------	----------	----------	----------	----------
*Drosophila_melanogaster*_NP_651095	----------	----------	----------	----------	----------
*Xenopus_laevis*_Q6DDJ2	----------	----------	----------	----------	----------
*Bos_taurus*_Q5E9Q1	----------	----------	----------	----------	----------
*Vitis_vinifera*_CAN70601	----------	----------	----------	----------	----------
Invertebrate_iridescent_virus_6_NP_149642	AWSFAFEKSL	NENGVEQCTL	LNEYIEGETF	STSIKNKMSY	LGFSSGKPFK
*Cryptococcus_neoformans*_var_*neoformans*_JEC21_XP_567800	EFIPDWRGDG	N-----VWEA	YRRTCEPSSQ	ARRLFG----	SLRANLKEGQ
*Arabidopsis_thaliana*_O80835	----------	----------	----------	----------	----------
*Vitis_vinifera*_CAN65871	----------	----------	----------	----------	----------

```
                                                   .........910 .........920 .........930 .........940 .........950
XpApiT_MH673348                                    ---------- ---------- ---------- ---------- ----------
Arabidopsis_thaliana_Q9SMT6                        ---------- ---------- ---------- ---------- ----------
Oryza_sativa_Japonica_XP_015636439                 ---------- ---------- ---------- ---------- ----------
Oryza_sativa_Japonica_Q0DEH1                       ---------- ---------- ---------- ---------- ----------
Arabidopsis_thaliana_O80836                        ---------- ---------- ---------- ---------- ----------
Arabidopsis_thaliana_Q9SH28                        ---------- ---------- ---------- ---------- ----------
Oryza_sativa_Japonica_Q6Z1Y8                       ---------- ---------- ---------- ---------- ----------
Arabidopsis_thaliana_Q56Y51                        ---------- ---------- ---------- ---------- ----------
Arabidopsis_thaliana_NP_172202                     ---------- ---------- ---------- ---------- ----------
Oryza_sativa_Japonica_XP_015633451                 ---------- ---------- ---------- ---------- ----------
Arabidopsis_thaliana_Q9M2D5                        ---------- ---------- ---------- ---------- ----------
Arabidopsis_thaliana_NP_191688                     ---------- ---------- ---------- ---------- ----------
Vitis_vinifera_CAN66409                            ---------- ---------- ---------- ---------- ----------
Arabidopsis_thaliana_Q0WS79                        ---------- ---------- ---------- ---------- ----------
Homo_sapiens_Q6UW63                                ---------- ---------- ---------- ---------- ----------
Mus_musculus_NP_076134                             ---------- ---------- ---------- ---------- ----------
Danio_rerio_Q7ZVE6                                 ---------- ---------- ---------- ---------- ----------
Nematostella_vectensis_XP_001639335                ---------- ---------- ---------- ---------- ----------
Drosophila_melanogaster_Q9VCU4                     ---------- ---------- ---------- ---------- ----------
Drosophila_melanogaster_NP_651095                  ---------- ---------- ---------- ---------- ----------
Xenopus_laevis_Q6DDJ2                              ---------- ---------- ---------- ---------- ----------
Bos_taurus_Q5E9Q1                                  ---------- ---------- ---------- ---------- ----------
Vitis_vinifera_CAN70601                            ---------- ---------- ---------- ---------- ----------
Invertebrate_iridescent_virus_6_NP_149642          NVLEVLFQII LSIQFAQEQC GFIHNDLTPW NIIIQTLKEP ITIQYPLFSG
Cryptococcus_neoformans_var_neoformans_JEC21_XP_567800 APVSRLADAG VTADAPSEDF FFPQSVDDKY DFCAHPWAHY NQGHFFS---
Arabidopsis_thaliana_O80835                        ---------- ---------- ---------- ---------- ----------
Vitis_vinifera_CAN65871                            ---------- ---------- ---------- ---------- ----------

                                                   .........960 .........970 .........980 .........990 .........1000
XpApiT_MH673348                                    ---------- ---------- ---------- ---------- ----------
Arabidopsis_thaliana_Q9SMT6                        ---------- ---------- ---------- ---------- ----------
Oryza_sativa_Japonica_XP_015636439                 ---------- ---------- ---------- ---------- ----------
Oryza_sativa_Japonica_Q0DEH1                       ---------- ---------- ---------- ---------- ----------
Arabidopsis_thaliana_O80836                        ---------- ---------- ---------- ---------- ----------
Arabidopsis_thaliana_Q9SH28                        ---------- ---------- ---------- ---------- ----------
Oryza_sativa_Japonica_Q6Z1Y8                       ---------- ---------- ---------- ---------- ----------
Arabidopsis_thaliana_Q56Y51                        ---------- ---------- ---------- ---------- ----------
Arabidopsis_thaliana_NP_172202                     ---------- ---------- ---------- ---------- ----------
Oryza_sativa_Japonica_XP_015633451                 ---------- ---------- ---------- ---------- ----------
Arabidopsis_thaliana_Q9M2D5                        ---------- ---------- ---------- ---------- ----------
Arabidopsis_thaliana_NP_191688                     ---------- ---------- ---------- ---------- ----------
Vitis_vinifera_CAN66409                            ---------- ---------- ---------- ---------- ----------
Arabidopsis_thaliana_Q0WS79                        ---------- ---------- ---------- ---------- ----------
Homo_sapiens_Q6UW63                                ---------- ---------- ---------- ---------- ----------
Mus_musculus_NP_076134                             ---------- ---------- ---------- ---------- ----------
Danio_rerio_Q7ZVE6                                 ---------- ---------- ---------- ---------- ----------
Nematostella_vectensis_XP_001639335                ---------- ---------- ---------- ---------- ----------
Drosophila_melanogaster_Q9VCU4                     ---------- ---------- ---------- ---------- ----------
Drosophila_melanogaster_NP_651095                  ---------- ---------- ---------- ---------- ----------
Xenopus_laevis_Q6DDJ2                              ---------- ---------- ---------- ---------- ----------
Bos_taurus_Q5E9Q1                                  ---------- ---------- ---------- ---------- ----------
Vitis_vinifera_CAN70601                            ---------- ---------- ---------- ---------- ----------
Invertebrate_iridescent_virus_6_NP_149642          IYKIITKHVP VIIDYGKSHV ATSPYGDFCF NELSVGNVIH YGVVNMFNMI
Cryptococcus_neoformans_var_neoformans_JEC21_XP_567800 DWRTIHALYP MFSPAKGLGY SD-------- ----ILIPSH YYFSSTKRYT
Arabidopsis_thaliana_O80835                        ---------- ---------- ---------- ---------- ----------
Vitis_vinifera_CAN65871                            ---------- ---------- ---------- ---------- ----------
```

```
                                                      .........1010.......1020.........1030.........1040.........1050
XpApiT_MH673348                                       ---------- ---------- ---------- ---------- ----------
Arabidopsis_thaliana_Q9SMT6                           ---------- ---------- ---------- ---------- ----------
Oryza_sativa_Japonica_XP_015636439                    ---------- ---------- ---------- ---------- ----------
Oryza_sativa_Japonica_Q0DEH1                          ---------- ---------- ---------- ---------- ----------
Arabidopsis_thaliana_O80836                           ---------- ---------- ---------- ---------- ----------
Arabidopsis_thaliana_Q9SH28                           ---------- ---------- ---------- ---------- ----------
Oryza_sativa_Japonica_Q6Z1Y8                          ---------- ---------- ---------- ---------- ----------
Arabidopsis_thaliana_Q56Y51                           ---------- ---------- ---------- ---------- ----------
Arabidopsis_thaliana_NP_172202                        ---------- ---------- ---------- ---------- ----------
Oryza_sativa_Japonica_XP_015633451                    ---------- ---------- ---------- ---------- ----------
Arabidopsis_thaliana_Q9M2D5                           ---------- ---------- ---------- ---------- ----------
Arabidopsis_thaliana_NP_191688                        ---------- ---------- ---------- ---------- ----------
Vitis_vinifera_CAN66409                               ---------- ---------- ---------- ---------- ----------
Arabidopsis_thaliana_Q0WS79                           ---------- ---------- ---------- ---------- ----------
Homo_sapiens_Q6UW63                                   ---------- ---------- ---------- ---------- ----------
Mus_musculus_NP_076134                                ---------- ---------- ---------- ---------- ----------
Danio_rerio_Q7ZVE6                                    ---------- ---------- ---------- ---------- ----------
Nematostella_vectensis_XP_001639335                   ---------- ---------- ---------- ---------- ----------
Drosophila_melanogaster_Q9VCU4                        ---------- ---------- ---------- ---------- ----------
Drosophila_melanogaster_NP_651095                     ---------- ---------- ---------- ---------- ----------
Xenopus_laevis_Q6DDJ2                                 ---------- ---------- ---------- ---------- ----------
Bos_taurus_Q5E9Q1                                     ---------- ---------- ---------- ---------- ----------
Vitis_vinifera_CAN70601                               ---------- ---------- ---------- ---------- ----------
Invertebrate_iridescent_virus_6_NP_149642             ECQDVFSIIL --YSCLDM-L TVNGTKFSQR ENDETEYDEQ SIIKMLNFFS
Cryptococcus_neoformans_var_neoformans_JEC21_XP_567800 YGWDPVNMVI KDVDDMETKW EDKSDDIFWR GA------TT GGGSSPP--G
Arabidopsis_thaliana_O80835                           ---------- ---------- ---------- ---------- ----------
Vitis_vinifera_CAN65871                               ---------- ---------- ---------- ---------- ----------

                                                      .........1060.........1070.........1080.........1090.........1100
XpApiT_MH673348                                       ---------- ---------- ---------- ---------- ----------
Arabidopsis_thaliana_Q9SMT6                           ---------- ---------- ---------- ---------- ----------
Oryza_sativa_Japonica_XP_015636439                    ---------- ---------- ---------- ---------- ----------
Oryza_sativa_Japonica_Q0DEH1                          ---------- ---------- ---------- ---------- ----------
Arabidopsis_thaliana_O80836                           ---------- ---------- ---------- ---------- ----------
Arabidopsis_thaliana_Q9SH28                           ---------- ---------- ---------- ---------- ----------
Oryza_sativa_Japonica_Q6Z1Y8                          ---------- ---------- ---------- ---------- ----------
Arabidopsis_thaliana_Q56Y51                           ---------- ---------- ---------- ---------- ----------
Arabidopsis_thaliana_NP_172202                        ---------- ---------- ---------- ---------- ----------
Oryza_sativa_Japonica_XP_015633451                    ---------- ---------- ---------- ---------- ----------
Arabidopsis_thaliana_Q9M2D5                           ---------- ---------- ---------- ---------- ----------
Arabidopsis_thaliana_NP_191688                        ---------- ---------- ---------- ---------- ----------
Vitis_vinifera_CAN66409                               ---------- ---------- ---------- ---------- ----------
Arabidopsis_thaliana_Q0WS79                           ---------- ---------- ---------- ---------- ----------
Homo_sapiens_Q6UW63                                   ---------- ---------- ---------- ---------- ----------
Mus_musculus_NP_076134                                ---------- ---------- ---------- ---------- ----------
Danio_rerio_Q7ZVE6                                    ---------- ---------- ---------- ---------- ----------
Nematostella_vectensis_XP_001639335                   ---------- ---------- ---------- ---------- ----------
Drosophila_melanogaster_Q9VCU4                        ---------- ---------- ---------- ---------- ----------
Drosophila_melanogaster_NP_651095                     ---------- ---------- ---------- ---------- ----------
Xenopus_laevis_Q6DDJ2                                 ---------- ---------- ---------- ---------- ----------
Bos_taurus_Q5E9Q1                                     ---------- ---------- ---------- ---------- ----------
Vitis_vinifera_CAN70601                               ---------- ---------- ---------- ---------- ----------
Invertebrate_iridescent_virus_6_NP_149642             PTELKTRKEA LSFIYQYKKY ENLIKAHETI NLEKSPIEFI KYFTSIFKKE
Cryptococcus_neoformans_var_neoformans_JEC21_XP_567800 FLAQYQRHRL IKMTSDSSNV NKTVVFADPP GTDHFISAQV PIGQLNKDMM
Arabidopsis_thaliana_O80835                           ---------- ---------- ---------- ---------- ----------
Vitis_vinifera_CAN65871                               ---------- ---------- ---------- ---------- ----------
```

```
                                                          .........1110.......1120.......1130.......1140.......1150
XpApiT_MH673348                                           ---------- ---------- ---------- ---------- ----------
Arabidopsis_thaliana_Q9SMT6                               ---------- ---------- ---------- ---------- ----------
Oryza_sativa_Japonica_XP_015636439                        ---------- ---------- ---------- ---------- ----------
Oryza_sativa_Japonica_Q0DEH1                              ---------- ---------- ---------- ---------- ----------
Arabidopsis_thaliana_O80836                               ---------- ---------- ---------- ---------- ----------
Arabidopsis_thaliana_Q9SH28                               ---------- ---------- ---------- ---------- ----------
Oryza_sativa_Japonica_Q6Z1Y8                              ---------- ---------- ---------- ---------- ----------
Arabidopsis_thaliana_Q56Y51                               ---------- ---------- ---------- ---------- ----------
Arabidopsis_thaliana_NP_172202                            ---------- ---------- ---------- ---------- ----------
Oryza_sativa_Japonica_XP_015633451                        ---------- ---------- ---------- ---------- ----------
Arabidopsis_thaliana_Q9M2D5                               ---------- ---------- ---------- ---------- ----------
Arabidopsis_thaliana_NP_191688                            ---------- ---------- ---------- ---------- ----------
Vitis_vinifera_CAN66409                                   ---------- ---------- ---------- ---------- ----------
Arabidopsis_thaliana_Q0WS79                               ---------- ---------- ---------- ---------- ----------
Homo_sapiens_Q6UW63                                       ---------- ---------- ---------- ---------- ----------
Mus_musculus_NP_076134                                    ---------- ---------- ---------- ---------- ----------
Danio_rerio_Q7ZVE6                                        ---------- ---------- ---------- ---------- ----------
Nematostella_vectensis_XP_001639335                       ---------- ---------- ---------- ---------- ----------
Drosophila_melanogaster_Q9VCU4                            ---------- ---------- ---------- ---------- ----------
Drosophila_melanogaster_NP_651095                         ---------- ---------- ---------- ---------- ----------
Xenopus_laevis_Q6DDJ2                                     ---------- ---------- ---------- ---------- ----------
Bos_taurus_Q5E9Q1                                         ---------- ---------- ---------- ---------- ----------
Vitis_vinifera_CAN70601                                   ---------- ---------- ---------- ---------- ----------
Invertebrate_iridescent_virus_6_NP_149642                 KVVYGLSRLD SKRTGLIMDF NNPKQIFDEA FAQNIQEVYI SFLNVPQNL-
Cryptococcus_neoformans_var_neoformans_JEC21_XP_567800    DVAF------ ---TKAVGCT QYPGGCDGMR ----KDHRFA DAVPLGENWR
Arabidopsis_thaliana_O80835                               ---------- ---------- ---------- ---------- ----------
Vitis_vinifera_CAN65871                                   ---------- ---------- ---------- ---------- ----------

                                                          .........1160.......1170.......1180.......1190.......1200
XpApiT_MH673348                                           ---------- ---------- ---------- ---------- ----------
Arabidopsis_thaliana_Q9SMT6                               ---------- ---------- ---------- ---------- ----------
Oryza_sativa_Japonica_XP_015636439                        ---------- ---------- ---------- ---------- ----------
Oryza_sativa_Japonica_Q0DEH1                              ---------- ---------- ---------- ---------- ----------
Arabidopsis_thaliana_O80836                               ---------- ---------- ---------- ---------- ----------
Arabidopsis_thaliana_Q9SH28                               ---------- ---------- ---------- ---------- ----------
Oryza_sativa_Japonica_Q6Z1Y8                              ---------- ---------- ---------- ---------- ----------
Arabidopsis_thaliana_Q56Y51                               ---------- ---------- ---------- ---------- ----------
Arabidopsis_thaliana_NP_172202                            ---------- ---------- ---------- ---------- ----------
Oryza_sativa_Japonica_XP_015633451                        ---------- ---------- ---------- ---------- ----------
Arabidopsis_thaliana_Q9M2D5                               ---------- ---------- ---------- ---------- ----------
Arabidopsis_thaliana_NP_191688                            ---------- ---------- ---------- ---------- ----------
Vitis_vinifera_CAN66409                                   ---------- ---------- ---------- ---------- ----------
Arabidopsis_thaliana_Q0WS79                               ---------- ---------- ---------- ---------- ----------
Homo_sapiens_Q6UW63                                       ---------- ---------- ---------- ---------- ----------
Mus_musculus_NP_076134                                    ---------- ---------- ---------- ---------- ----------
Danio_rerio_Q7ZVE6                                        ---------- ---------- ---------- ---------- ----------
Nematostella_vectensis_XP_001639335                       ---------- ---------- ---------- ---------- ----------
Drosophila_melanogaster_Q9VCU4                            ---------- ---------- ---------- ---------- ----------
Drosophila_melanogaster_NP_651095                         ---------- ---------- ---------- ---------- ----------
Xenopus_laevis_Q6DDJ2                                     ---------- ---------- ---------- ---------- ----------
Bos_taurus_Q5E9Q1                                         ---------- ---------- ---------- ---------- ----------
Vitis_vinifera_CAN70601                                   ---------- ---------- ---------- ---------- ----------
Invertebrate_iridescent_virus_6_NP_149642                 YKCTLPQPKT KIELYMVAQL LIKCLKDTLK DYRIFAEKSS LRKESFHNDI
Cryptococcus_neoformans_var_neoformans_JEC21_XP_567800    HKYLIDIDGM GYSARLFALL KSESAVLKST VYTEFMSE-- --WLQPWLHY
Arabidopsis_thaliana_O80835                               ---------- ---------- ---------- ---------- ----------
Vitis_vinifera_CAN65871                                   ---------- ---------- ---------- ---------- ----------
```

```
                                                          .........1210.......1220.......1230.......1240.......1250
XpApiT_MH673348                                           ---------- ---------- ---------- ---------- ----------
Arabidopsis_thaliana_Q9SMT6                               ---------- ---------- ---------- ---------- ----------
Oryza_sativa_Japonica_XP_015636439                        ---------- ---------- ---------- ---------- ----------
Oryza_sativa_Japonica_Q0DEH1                              ---------- ---------- ---------- ---------- ----------
Arabidopsis_thaliana_O80836                               ---------- ---------- ---------- ---------- ----------
Arabidopsis_thaliana_Q9SH28                               ---------- ---------- ---------- ---------- ----------
Oryza_sativa_Japonica_Q6Z1Y8                              ---------- ---------- ---------- ---------- ----------
Arabidopsis_thaliana_Q56Y51                               ---------- ---------- ---------- ---------- ----------
Arabidopsis_thaliana_NP_172202                            ---------- ---------- ---------- ---------- ----------
Oryza_sativa_Japonica_XP_015633451                        ---------- ---------- ---------- ---------- ----------
Arabidopsis_thaliana_Q9M2D5                               ---------- ---------- ---------- ---------- ----------
Arabidopsis_thaliana_NP_191688                            ---------- ---------- ---------- ---------- ----------
Vitis_vinifera_CAN66409                                   ---------- ---------- ---------- ---------- ----------
Arabidopsis_thaliana_Q0WS79                               ---------- ---------- ---------- ---------- ----------
Homo_sapiens_Q6UW63                                       ---------- ---------- ---------- ---------- ----------
Mus_musculus_NP_076134                                    ---------- ---------- ---------- ---------- ----------
Danio_rerio_Q7ZVE6                                        ---------- ---------- ---------- ---------- ----------
Nematostella_vectensis_XP_001639335                       ---------- ---------- ---------- ---------- ----------
Drosophila_melanogaster_Q9VCU4                            ---------- ---------- ---------- ---------- ----------
Drosophila_melanogaster_NP_651095                         ---------- ---------- ---------- ---------- ----------
Xenopus_laevis_Q6DDJ2                                     ---------- ---------- ---------- ---------- ----------
Bos_taurus_Q5E9Q1                                         ---------- ---------- ---------- ---------- ----------
Vitis_vinifera_CAN70601                                   ---------- ---------- ---------- ---------- ----------
Invertebrate_iridescent_virus_6_NP_149642                 IKFENAINFI TTFYSKEISK HGNDSWVIPE LCENNQIEIS RNYFRTTSDT
Cryptococcus_neoformans_var_neoformans_JEC21_XP_567800    IPIS---QMY QEIYNVHAFF SGP-----SK AMLDASNSTR TLYQQPGVHT
Arabidopsis_thaliana_O80835                               ---------- ---------- ---------- ---------- ----------
Vitis_vinifera_CAN65871                                   ---------- ---------- ---------- ---------- ----------

                                                          .........1260.......1270.......1280.......1290.......1300
XpApiT_MH673348                                           ---------- ---------- ---------- ---------- ----------
Arabidopsis_thaliana_Q9SMT6                               ---------- ---------- ---------- ---------- ----------
Oryza_sativa_Japonica_XP_015636439                        ---------- ---------- ---------- ---------- ----------
Oryza_sativa_Japonica_Q0DEH1                              ---------- ---------- ---------- ---------- ----------
Arabidopsis_thaliana_O80836                               ---------- ---------- ---------- ---------- ----------
Arabidopsis_thaliana_Q9SH28                               ---------- ---------- ---------- ---------- ----------
Oryza_sativa_Japonica_Q6Z1Y8                              ---------- ---------- ---------- ---------- ----------
Arabidopsis_thaliana_Q56Y51                               ---------- ---------- ---------- ---------- ----------
Arabidopsis_thaliana_NP_172202                            ---------- ---------- ---------- ---------- ----------
Oryza_sativa_Japonica_XP_015633451                        ---------- ---------- ---------- ---------- ----------
Arabidopsis_thaliana_Q9M2D5                               ---------- ---------- ---------- ---------- ----------
Arabidopsis_thaliana_NP_191688                            ---------- ---------- ---------- ---------- ----------
Vitis_vinifera_CAN66409                                   ---------- ---------- ---------- ---------- ----------
Arabidopsis_thaliana_Q0WS79                               ---------- ---------- ---------- ---------- ----------
Homo_sapiens_Q6UW63                                       ---------- ---------- ---------- ---------- ----------
Mus_musculus_NP_076134                                    ---------- ---------- ---------- ---------- ----------
Danio_rerio_Q7ZVE6                                        ---------- ---------- ---------- ---------- ----------
Nematostella_vectensis_XP_001639335                       ---------- ---------- ---------- ---------- ----------
Drosophila_melanogaster_Q9VCU4                            ---------- ---------- ---------- ---------- ----------
Drosophila_melanogaster_NP_651095                         ---------- ---------- ---------- ---------- ----------
Xenopus_laevis_Q6DDJ2                                     ---------- ---------- ---------- ---------- ----------
Bos_taurus_Q5E9Q1                                         ---------- ---------- ---------- ---------- ----------
Vitis_vinifera_CAN70601                                   ---------- ---------- ---------- ---------- ----------
Invertebrate_iridescent_virus_6_NP_149642                 LNYSIKPCTD LTFFKKTILN ILNWTDDEGL FEIKAEDRVE ISTQLKFVLE
Cryptococcus_neoformans_var_neoformans_JEC21_XP_567800    KKFD-----G DAELRKIAKA GREWMF---- TIGRKID--- -MEIYVYRLC
Arabidopsis_thaliana_O80835                               ---------- ---------- ---------- ---------- ----------
Vitis_vinifera_CAN65871                                   ---------- ---------- ---------- ---------- ----------
```

	. . . . 1310 . . . . 1320 . . . .
XpApiT_MH673348	- - - - - - - - - - - - - - - - - - - - - - - - - - - -
*Arabidopsis_thaliana*_Q9SMT6	- - - - - - - - - - - - - - - - - - - - - - - - - - - -
*Oryza_sativa*_Japonica_XP_015636439	- - - - - - - - - - - - - - - - - - - - - - - - - - - -
*Oryza_sativa*_Japonica_Q0DEH1	- - - - - - - - - - - - - - - - - - - - - - - - - - - -
*Arabidopsis_thaliana*_O80836	- - - - - - - - - - - - - - - - - - - - - - - - - - - -
*Arabidopsis_thaliana*_Q9SH28	- - - - - - - - - - - - - - - - - - - - - - - - - - - -
*Oryza_sativa*_Japonica_Q6Z1Y8	- - - - - - - - - - - - - - - - - - - - - - - - - - - -
*Arabidopsis_thaliana*_Q56Y51	- - - - - - - - - - - - - - - - - - - - - - - - - - - -
*Arabidopsis_thaliana*_NP_172202	- - - - - - - - - - - - - - - - - - - - - - - - - - - -
*Oryza_sativa*_Japonica_XP_015633451	- - - - - - - - - - - - - - - - - - - - - - - - - - - -
*Arabidopsis_thaliana*_Q9M2D5	- - - - - - - - - - - - - - - - - - - - - - - - - - - -
*Arabidopsis_thaliana*_NP_191688	- - - - - - - - - - - - - - - - - - - - - - - - - - - -
*Vitis_vinifera*_CAN66409	- - - - - - - - - - - - - - - - - - - - - - - - - - - -
*Arabidopsis_thaliana*_Q0WS79	- - - - - - - - - - - - - - - - - - - - - - - - - - - -
*Homo_sapiens*_Q6UW63	- - - - - - - - - - - - - - - - - - - - - - - - - - - -
*Mus_musculus*_NP_076134	- - - - - - - - - - - - - - - - - - - - - - - - - - - -
*Danio_rerio*_Q7ZVE6	- - - - - - - - - - - - - - - - - - - - - - - - - - - -
*Nematostella_vectensis*_XP_001639335	- - - - - - - - - - - - - - - - - - - - - - - - - - - -
*Drosophila_melanogaster*_Q9VCU4	- - - - - - - - - - - - - - - - - - - - - - - - - - - -
*Drosophila_melanogaster*_NP_651095	- - - - - - - - - - - - - - - - - - - - - - - - - - - -
*Xenopus_laevis*_Q6DDJ2	- - - - - - - - - - - - - - - - - - - - - - - - - - - -
*Bos_taurus*_Q5E9Q1	- - - - - - - - - - - - - - - - - - - - - - - - - - - -
*Vitis_vinifera*_CAN70601	- - - - - - - - - - - - - - - - - - - - - - - - - - - -
Invertebrate_iridescent_virus_6_NP_149642	S T F K D K K I S A N N N T Y I V Y K K Y C N K K Y C I
*Cryptococcus_neoformans*_var_*neoformans*_JEC21_XP_567800	L E W - - A R L M A D D R E A M T Y N G - - - - - - - -
*Arabidopsis_thaliana*_O80835	- - - - - - - - - - - - - - - - - - - - - - - - - - - -
*Vitis_vinifera*_CAN65871	- - - - - - - - - - - - - - - - - - - - - - - - - - - -
