# Supplementary material for: Identification of an apiosyltransferase in the plant pathogen Xanthomonas pisi
Source: PLoS One. 2018 Oct 18;13(10):e0206187. doi: 10.1371/journal.pone.0206187 (PMC6193724; doi:10.1371/journal.pone.0206187)
Supplement: S2 Fig — Full amino acid alignment of XpXylT (MH673349) and representative members of GT family 2 from the following organisms: Jannaschia sp CCS1 (ABD53137; ABD56186; ABD56187; ABD53753; ABD56179), Dinoroseobacter shibae (WP 012180235; WP 012179538), Ruegeria pomeroyi (AAV93367; AAV93368; AAV94731; AAV94670; AAV95896; AAV95846; AAV94320), Ruegeria sp TM1040 (ABF62750; ABF62751; ABF64507), Paracoccus denitrificans (WP 011748578), Rhizobium etli CFN 42 (ABC92155), Geodermatophilus obscurus (WP 012946629), Nostoc sp PCC 7120 (BAB73679), Pseudovibrio sp FO-BEG1 (WP 014283852), Ochrobactrum anthropi (WP 012090710), Beijerinckia indica (WP 012385681), Sinorhizobium fredii (WP 012708451), Synechococcus sp WH 8102 (CAE07032), Methylobacterium radiotolerans (WP 012317373), Porphyromonas asaccharolytica (WP 004330966), Gluconobacter oxydans (AAW60025), Streptococcus suis (CYU93668), Acidiphilium cryptum (ABQ29611), Selenomonas sputigena (WP 006193730), Thalassiosira pseudonana (XP 002288249), Nematostella vectensis (XP 001635452), Rattus norvegicus (NP 001099537), Gloeobacter violaceus (BAC90139), Granulibacter bethesdensis (ABI61222), Rhodothermus marinus (WP 012843638), Paenibacillus sp JDR-2 (WP 015843614), Methylorubrum extorquens (WP 003600880). Sequences were aligned with PRALINE [63] using the BLOSUM62 scoring matrix. (PDF) [file pone.0206187.s002.pdf]

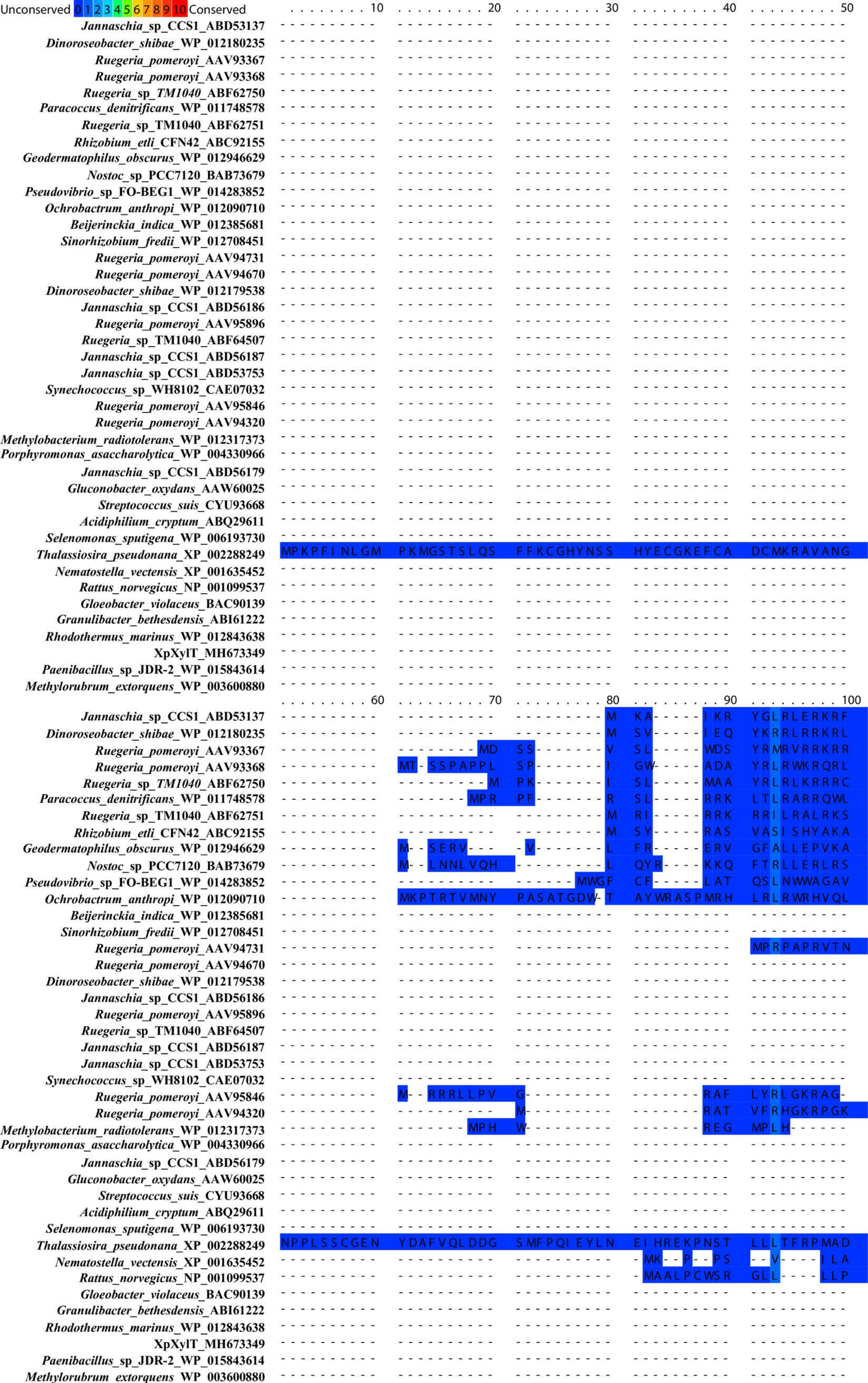

Unconserved 0 1 2 3 4 5 6 7 8 9 10 Conserved
10
20
30
40
50
Jannaschia_sp_CCS1_ABD53137
Dinoroseobacter_shibae_WP_012180235
Ruegeria_pomeroyi_AAV93367
Ruegeria_pomeroyi_AAV93368
Ruegeria_sp_TM1040_ABF62750
Paracoccus_denitrificans_WP_011748578
Ruegeria_sp_TM1040_ABF62751
Rhizobium_etli_CFN42_ABC92155
Geodermatophilus_obscurus_WP_012946629
Nostoc_sp_PCC7120_BAB73679
Pseudovibrio_sp_FO-BEG1_WP_014283852
Ochrobactrum_anthropi_WP_012090710
Beijerinckia_indica_WP_012385681
Sinorhizobium_fredii_WP_012708451
Ruegeria_pomeroyi_AAV94731
Ruegeria_pomeroyi_AAV94670
Dinoroseobacter_shibae_WP_012179538
Jannaschia_sp_CCS1_ABD56186
Ruegeria_pomeroyi_AAV95896
Ruegeria_sp_TM1040_ABF64507
Jannaschia_sp_CCS1_ABD56187
Jannaschia_sp_CCS1_ABD53753
Synechococcus_sp_WH8102_CAE07032
Ruegeria_pomeroyi_AAV95846
Ruegeria_pomeroyi_AAV94320
Methylobacterium_radiotolerans_WP_012317373
Porphyromonas_asaccharolytica_WP_004330966
Jannaschia_sp_CCS1_ABD56179
Gluconobacter_oxydans_AAW60025
Streptococcus_suis_CYU93668
Acidiphilium_cryptum_ABQ29611
Selenomonas_sputigena_WP_006193730
Thalassiosira_pseudonana_XP_002288249
Nematostella_vectensis_XP_001635452
Rattus_norvegicus_NP_001099537
Gloeobacter_violaceus_BAC90139
Granulibacter_bethesdensis_ABI61222
Rhodothermus_marinus_WP_012843638
XpXylT_MH673349
Paenibacillus_sp_JDR-2_WP_015843614
Methylorubrum_extorquens_WP_003600880
60
70
80
90
100

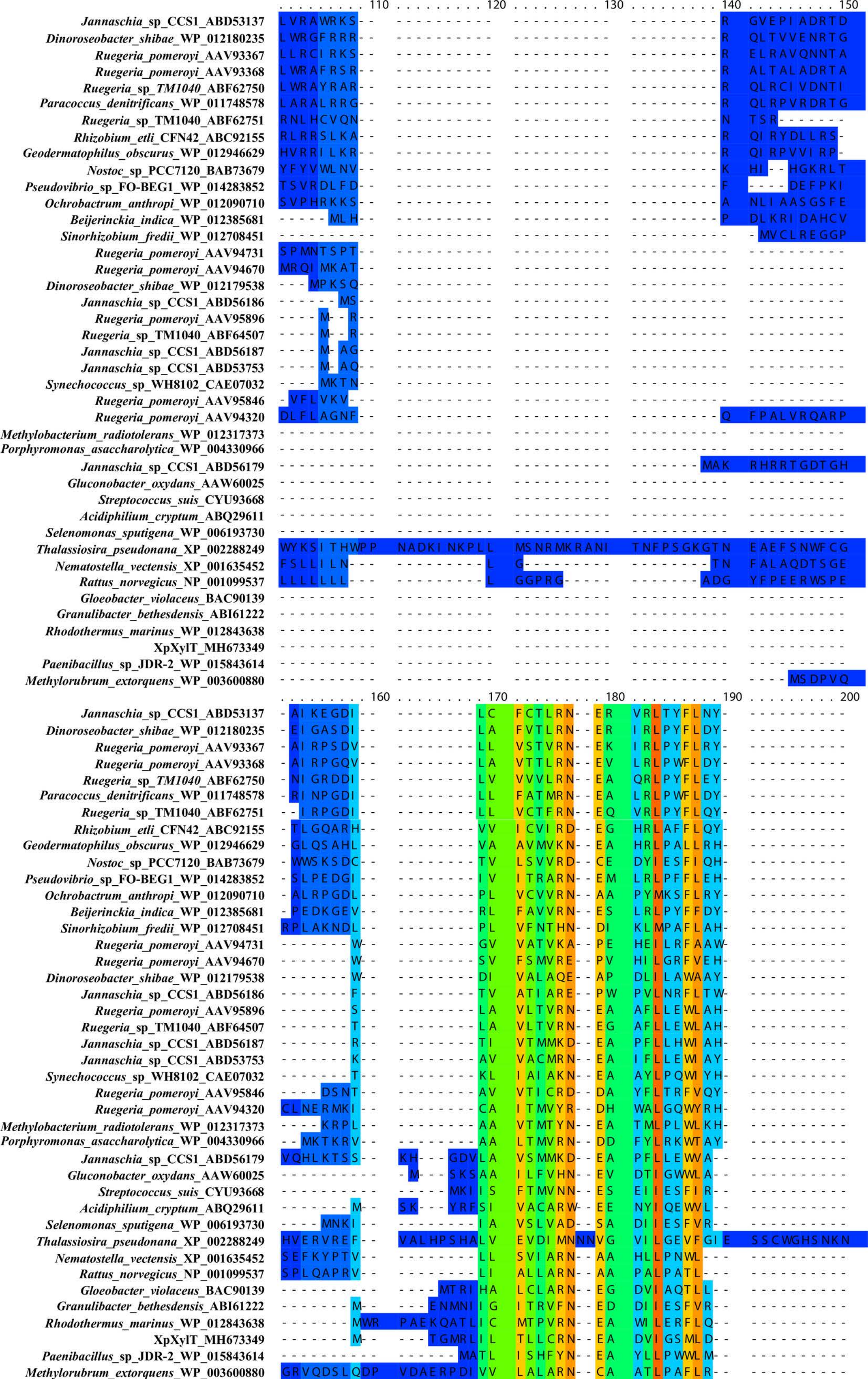
110 120 130 140 150
160 170 180 190 200
Jannaschia_sp_CCS1_ABD53137
Dinoroseobacter_shibae_WP_012180235
Ruegeria_pomeroyi_AAV93367
Ruegeria_pomeroyi_AAV93368
Ruegeria_sp_TM1040_ABF62750
Paracoccus_denitrificans_WP_011748578
Ruegeria_sp_TM1040_ABF62751
Rhizobium_etli_CFN42_ABC92155
Geodermatophilus_obscurus_WP_012946629
Nostoc_sp_PCC7120_BAB73679
Pseudovibrio_sp_FO-BEG1_WP_014283852
Ochrobactrum_anthropi_WP_012090710
Beijerinckia_indica_WP_012385681
Sinorhizobium_fredii_WP_012708451
Ruegeria_pomeroyi_AAV94731
Ruegeria_pomeroyi_AAV94670
Dinoroseobacter_shibae_WP_012179538
Jannaschia_sp_CCS1_ABD56186
Ruegeria_pomeroyi_AAV95896
Ruegeria_sp_TM1040_ABF64507
Jannaschia_sp_CCS1_ABD56187
Jannaschia_sp_CCS1_ABD53753
Synechococcus_sp_WH8102_CAE07032
Ruegeria_pomeroyi_AAV95846
Ruegeria_pomeroyi_AAV94320
Methylobacterium_radiotolerans_WP_012317373
Porphyromonas_asaccharolytica_WP_004330966
Jannaschia_sp_CCS1_ABD56179
Gluconobacter_oxydans_AAW60025
Streptococcus_suis_CYU93668
Acidiphilium_cryptum_ABQ29611
Selenomonas_sputigena_WP_006193730
Thalassiosira_pseudonana_XP_002288249
Nematostella_vectensis_XP_001635452
Rattus_norvegicus_NP_001099537
Gloeobacter_violaceus_BAC90139
Granulibacter_bethesdensis_ABI61222
Rhodothermus_marinus_WP_012843638
XpXylT_MH673349
Paenibacillus_sp_JDR-2_WP_015843614
Methylorubrum_extorquens_WP_003600880

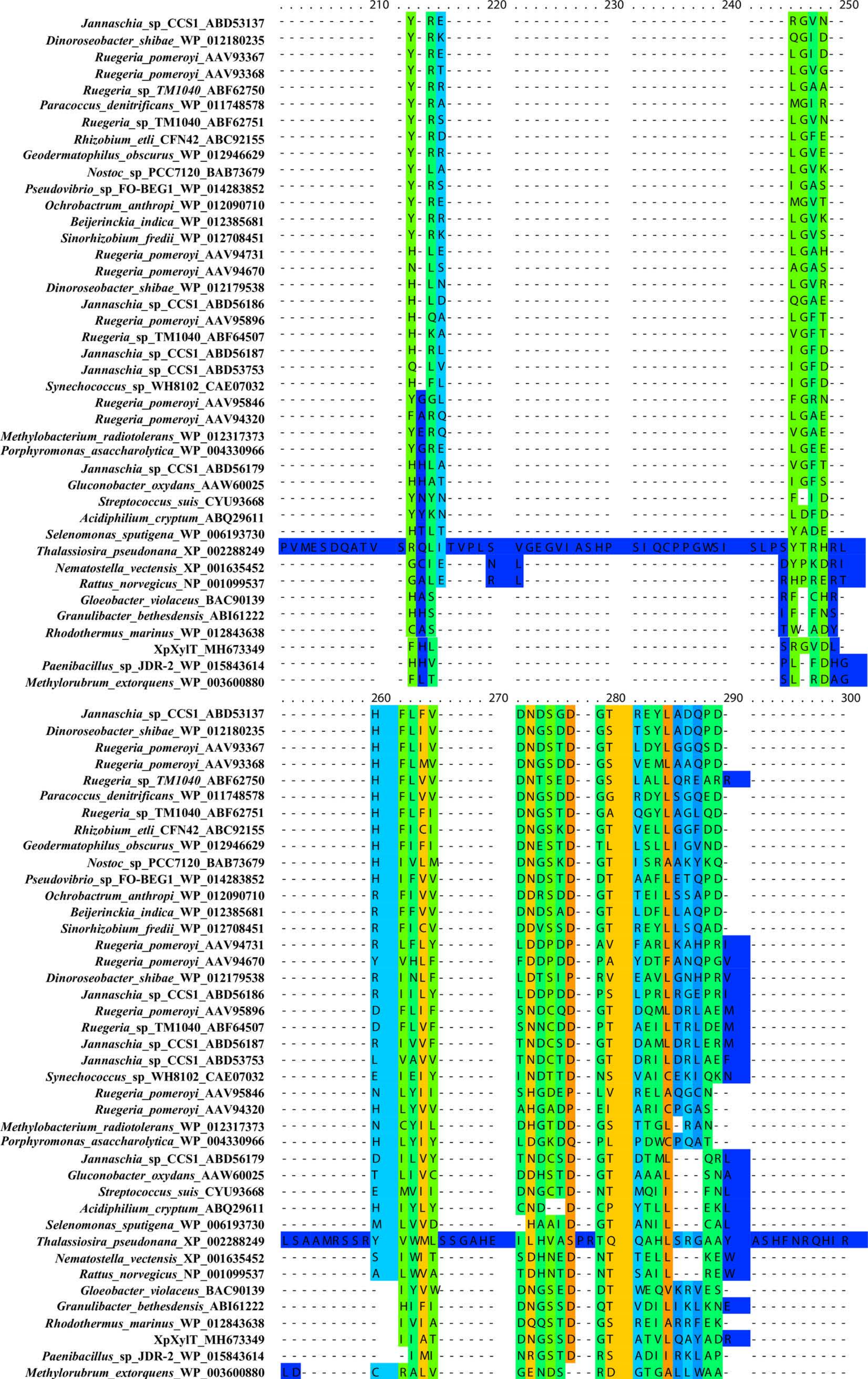
210
220
230
240
250
260
270
280
290
300
Jannaschia_sp_CCS1_ABD53137
Dinoroseobacter_shibae_WP_012180235
Ruegeria_pomeroyi_AAV93367
Ruegeria_pomeroyi_AAV93368
Ruegeria_sp_TM1040_ABF62750
Paracoccus_denitrificans_WP_011748578
Ruegeria_sp_TM1040_ABF62751
Rhizobium_etli_CFN42_ABC92155
Geodermatophilus_obscurus_WP_012946629
Nostoc_sp_PCC7120_BAB73679
Pseudovibrio_sp_FO-BEG1_WP_014283852
Ochrobactrum_anthropi_WP_012090710
Beijerinckia_indica_WP_012385681
Sinorhizobium_fredii_WP_012708451
Ruegeria_pomeroyi_AAV94731
Ruegeria_pomeroyi_AAV94670
Dinoroseobacter_shibae_WP_012179538
Jannaschia_sp_CCS1_ABD56186
Ruegeria_pomeroyi_AAV95896
Ruegeria_sp_TM1040_ABF64507
Jannaschia_sp_CCS1_ABD56187
Jannaschia_sp_CCS1_ABD53753
Synechococcus_sp_WH8102_CAE07032
Ruegeria_pomeroyi_AAV95846
Ruegeria_pomeroyi_AAV94320
Methylobacterium_radiotolerans_WP_012317373
Porphyromonas_asaccharolytica_WP_004330966
Jannaschia_sp_CCS1_ABD56179
Gluconobacter_oxydans_AAW60025
Streptococcus_suis_CYU93668
Acidiphilium_cryptum_ABQ29611
Selenomonas_sputigena_WP_006193730
Thalassiosira_pseudonana_XP_002288249
Nematostella_vectensis_XP_001635452
Rattus_norvegicus_NP_001099537
Gloeobacter_violaceus_BAC90139
Granulibacter_bethesdensis_ABI61222
Rhodothermus_marinus_WP_012843638
XpXylT_MH673349
Paenibacillus_sp_JDR-2_WP_015843614
Methylorubrum_extorquens_WP_003600880

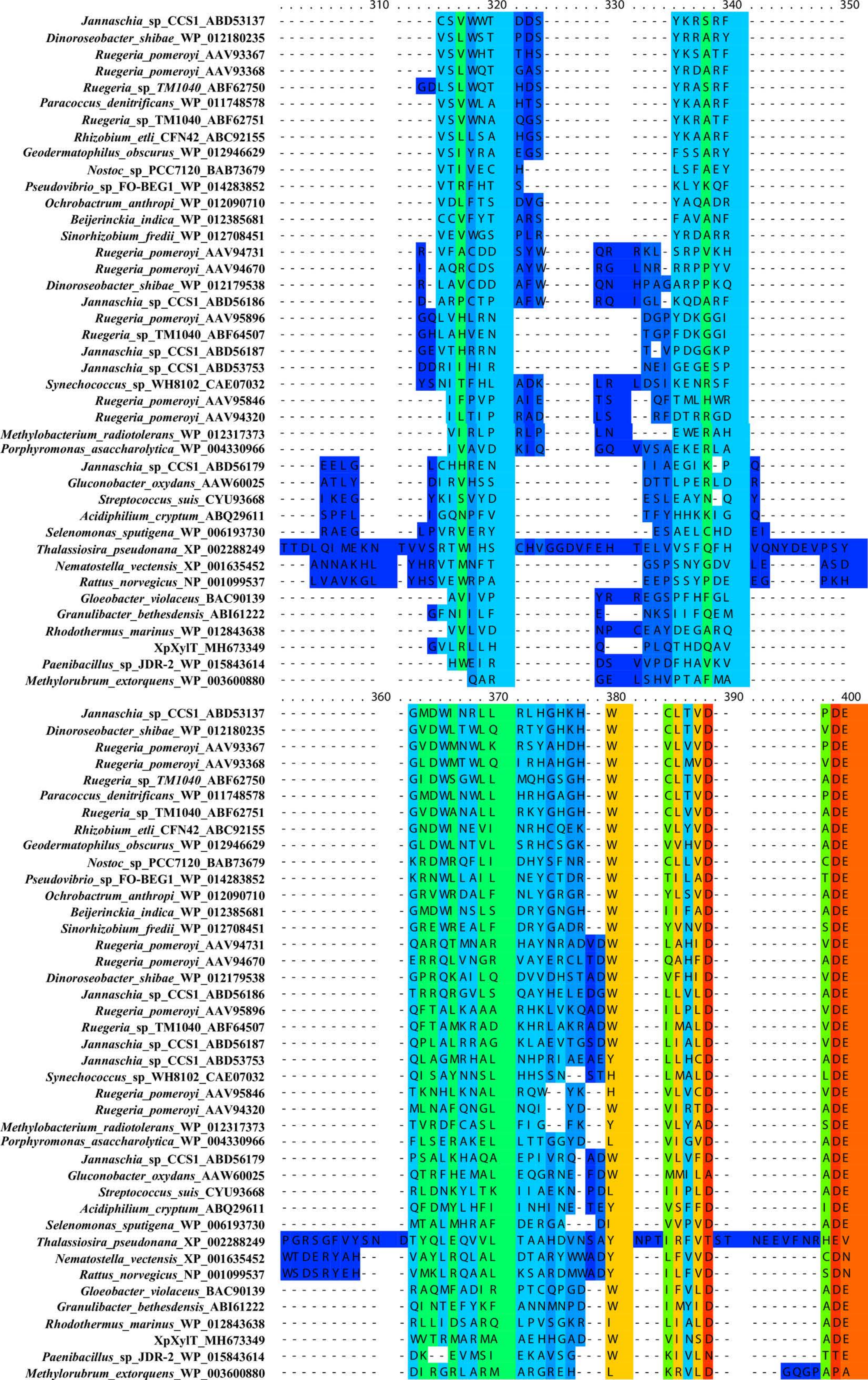
310
320
330
340
350
360
370
380
390
400
Jannaschia_sp_CCS1_ABD53137
Dinoroseobacter_shibae_WP_012180235
Ruegeria_pomeroyi_AAV93367
Ruegeria_pomeroyi_AAV93368
Ruegeria_sp_TM1040_ABF62750
Paracoccus_denitrificans_WP_011748578
Ruegeria_sp_TM1040_ABF62751
Rhizobium_etli_CFN42_ABC92155
Geodermatophilus_obscurus_WP_012946629
Nostoc_sp_PCC7120_BAB73679
Pseudovibrio_sp_FO-BEG1_WP_014283852
Ochrobactrum_anthropi_WP_012090710
Beijerinckia_indica_WP_012385681
Sinorhizobium_fredii_WP_012708451
Ruegeria_pomeroyi_AAV94731
Ruegeria_pomeroyi_AAV94670
Dinoroseobacter_shibae_WP_012179538
Jannaschia_sp_CCS1_ABD56186
Ruegeria_pomeroyi_AAV95896
Ruegeria_sp_TM1040_ABF64507
Jannaschia_sp_CCS1_ABD56187
Jannaschia_sp_CCS1_ABD53753
Synechococcus_sp_WH8102_CAE07032
Ruegeria_pomeroyi_AAV95846
Ruegeria_pomeroyi_AAV94320
Methylobacterium_radiotolerans_WP_012317373
Porphyromonas_asaccharolytica_WP_004330966
Jannaschia_sp_CCS1_ABD56179
Gluconobacter_oxydans_AAW60025
Streptococcus_suis_CYU93668
Acidiphilium_cryptum_ABQ29611
Selenomonas_sputigena_WP_006193730
Thalassiosira_pseudonana_XP_002288249
Nematostella_vectensis_XP_001635452
Rattus_norvegicus_NP_001099537
Gloeobacter_violaceus_BAC90139
Granulibacter_bethesdensis_ABI61222
Rhodothermus_marinus_WP_012843638
XpXylT_MH673349
Paenibacillus_sp_JDR-2_WP_015843614
Methylorubrum_extorquens_WP_003600880

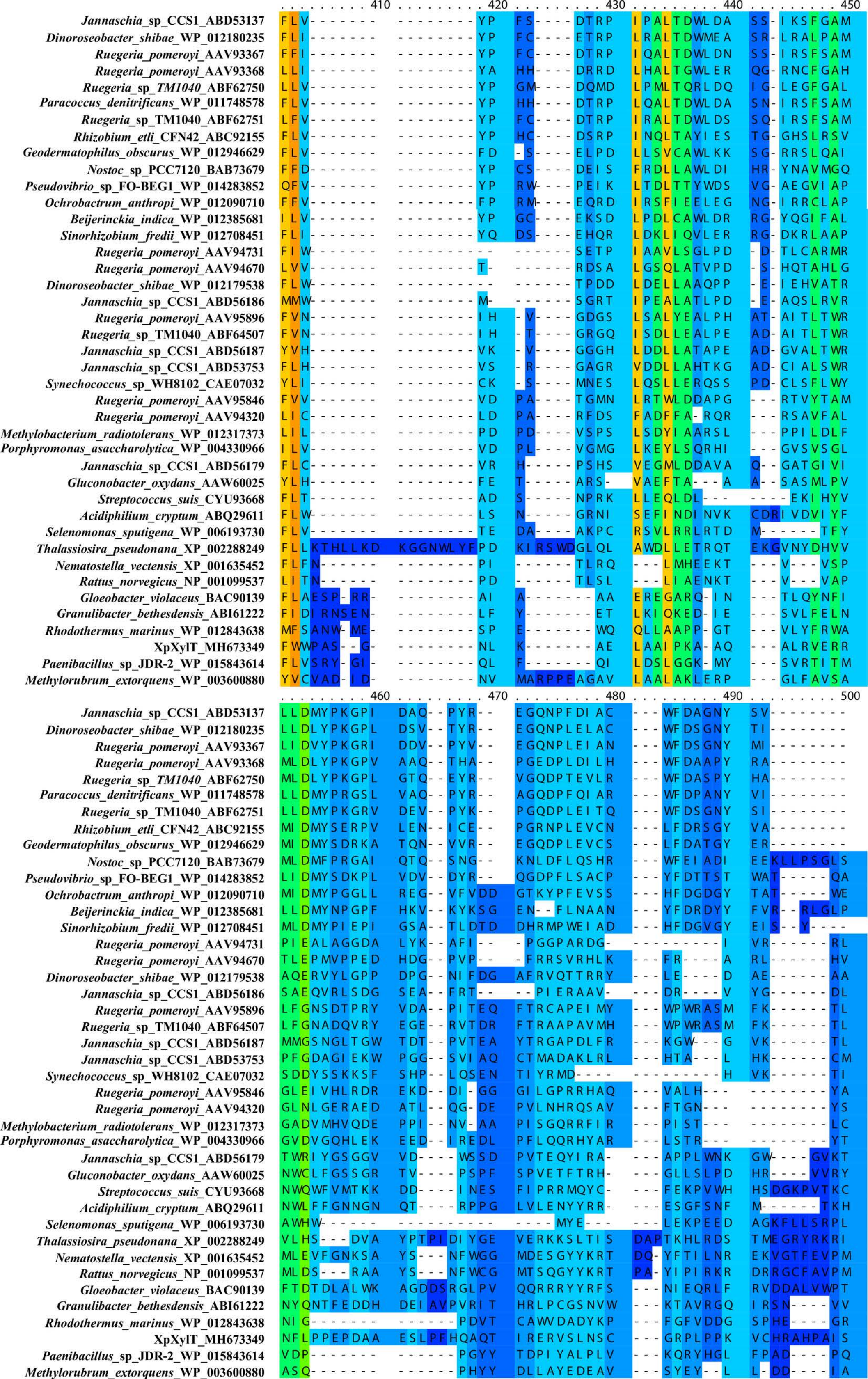

410
420
430
440
450
Jannaschia_sp_CCS1_ABD53137
Dinoroseobacter_shibae_WP_012180235
Ruegeria_pomeroyi_AAV93367
Ruegeria_pomeroyi_AAV93368
Ruegeria_sp_TM1040_ABF62750
Paracoccus_denitrificans_WP_011748578
Ruegeria_sp_TM1040_ABF62751
Rhizobium_etli_CFN42_ABC92155
Geodermatophilus_obscurus_WP_012946629
Nostoc_sp_PCC7120_BAB73679
Pseudovibrio_sp_FO-BEG1_WP_014283852
Ochrobactrum_anthropi_WP_012090710
Beijerinckia_indica_WP_012385681
Sinorhizobium_fredii_WP_012708451
Ruegeria_pomeroyi_AAV94731
Ruegeria_pomeroyi_AAV94670
Dinoroseobacter_shibae_WP_012179538
Jannaschia_sp_CCS1_ABD56186
Ruegeria_pomeroyi_AAV95896
Ruegeria_sp_TM1040_ABF64507
Jannaschia_sp_CCS1_ABD56187
Jannaschia_sp_CCS1_ABD53753
Synechococcus_sp_WH8102_CAE07032
Ruegeria_pomeroyi_AAV95846
Ruegeria_pomeroyi_AAV94320
Methylobacterium_radiotolerans_WP_012317373
Porphyromonas_asaccharolytica_WP_004330966
Jannaschia_sp_CCS1_ABD56179
Gluconobacter_oxydans_AAW60025
Streptococcus_suis_CYU93668
Acidiphilium_cryptum_ABQ29611
Selenomonas_sputigena_WP_006193730
Thalassiosira_pseudonana_XP_002288249
Nematostella_vectensis_XP_001635452
Rattus_norvegicus_NP_001099537
Gloeobacter_violaceus_BAC90139
Granulibacter_bethesdensis_ABI61222
Rhodothermus_marinus_WP_012843638
XpXylT_MH673349
Paenibacillus_sp_JDR-2_WP_015843614
Methylorubrum_extorquens_WP_003600880
460
470
480
490
500

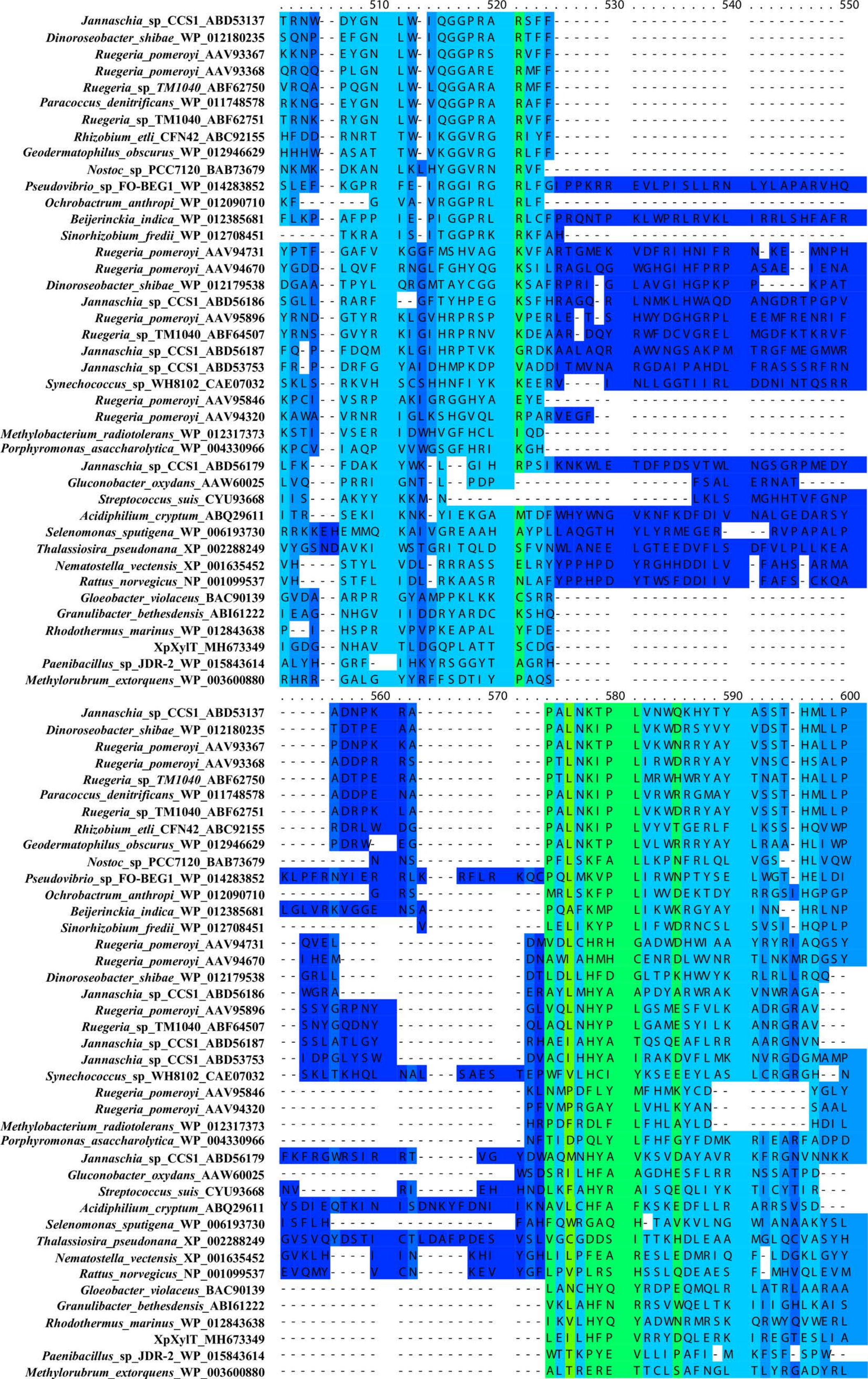

510
520
530
540
550
560
570
580
590
600
Jannaschia_sp_CCS1_ABD53137
Dinoroseobacter_shibae_WP_012180235
Ruegeria_pomeroyi_AAV93367
Ruegeria_pomeroyi_AAV93368
Ruegeria_sp_TM1040_ABF62750
Paracoccus_denitrificans_WP_011748578
Ruegeria_sp_TM1040_ABF62751
Rhizobium_etli_CFN42_ABC92155
Geodermatophilus_obscurus_WP_012946629
Nostoc_sp_PCC7120_BAB73679
Pseudovibrio_sp_FO-BEG1_WP_014283852
Ochrobactrum_anthropi_WP_012090710
Beijerinckia_indica_WP_012385681
Sinorhizobium_fredii_WP_012708451
Ruegeria_pomeroyi_AAV94731
Ruegeria_pomeroyi_AAV94670
Dinoroseobacter_shibae_WP_012179538
Jannaschia_sp_CCS1_ABD56186
Ruegeria_pomeroyi_AAV95896
Ruegeria_sp_TM1040_ABF64507
Jannaschia_sp_CCS1_ABD56187
Jannaschia_sp_CCS1_ABD53753
Synechococcus_sp_WH8102_CAE07032
Ruegeria_pomeroyi_AAV95846
Ruegeria_pomeroyi_AAV94320
Methylobacterium_radiotolerans_WP_012317373
Porphyromonas_asaccharolytica_WP_004330966
Jannaschia_sp_CCS1_ABD56179
Gluconobacter_oxydans_AAW60025
Streptococcus_suis_CYU93668
Acidiphilium_cryptum_ABQ29611
Selenomonas_sputigena_WP_006193730
Thalassiosira_pseudonana_XP_002288249
Nematostella_vectensis_XP_001635452
Rattus_norvegicus_NP_001099537
Gloeobacter_violaceus_BAC90139
Granulibacter_bethesdensis_ABI61222
Rhodothermus_marinus_WP_012843638
XpXylT_MH673349
Paenibacillus_sp_JDR-2_WP_015843614
Methylorubrum_extorquens_WP_003600880

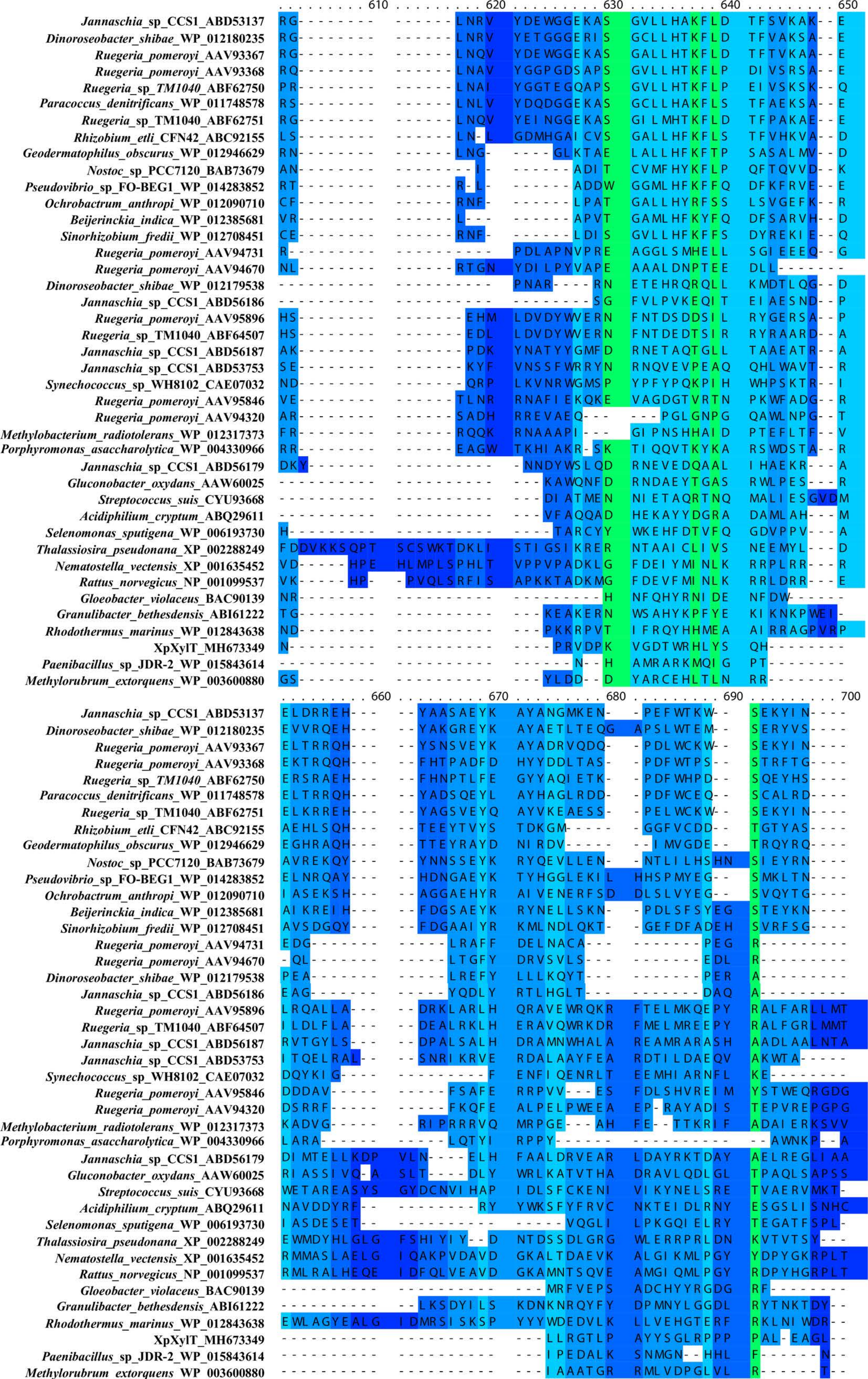
610
620
630
640
650
660
670
680
690
700
Jannaschia_sp_CCS1_ABD53137
Dinoroseobacter_shibae_WP_012180235
Ruegeria_pomeroyi_AAV93367
Ruegeria_pomeroyi_AAV93368
Ruegeria_sp_TM1040_ABF62750
Paracoccus_denitrificans_WP_011748578
Ruegeria_sp_TM1040_ABF62751
Rhizobium_etli_CFN42_ABC92155
Geodermatophilus_obscurus_WP_012946629
Nostoc_sp_PCC7120_BAB73679
Pseudovibrio_sp_FO-BEG1_WP_014283852
Ochrobactrum_anthropi_WP_012090710
Beijerinckia_indica_WP_012385681
Sinorhizobium_fredii_WP_012708451
Ruegeria_pomeroyi_AAV94731
Ruegeria_pomeroyi_AAV94670
Dinoroseobacter_shibae_WP_012179538
Jannaschia_sp_CCS1_ABD56186
Ruegeria_pomeroyi_AAV95896
Ruegeria_sp_TM1040_ABF64507
Jannaschia_sp_CCS1_ABD56187
Jannaschia_sp_CCS1_ABD53753
Synechococcus_sp_WH8102_CAE07032
Ruegeria_pomeroyi_AAV95846
Ruegeria_pomeroyi_AAV94320
Methylobacterium_radiotolerans_WP_012317373
Porphyromonas_asaccharolytica_WP_004330966
Jannaschia_sp_CCS1_ABD56179
Gluconobacter_oxydans_AAW60025
Streptococcus_suis_CYU93668
Acidiphilium_cryptum_ABQ29611
Selenomonas_sputigena_WP_006193730
Thalassiosira_pseudonana_XP_002288249
Nematostella_vectensis_XP_001635452
Rattus_norvegicus_NP_001099537
Gloeobacter_violaceus_BAC90139
Granulibacter_bethesdensis_ABI61222
Rhodothermus_marinus_WP_012843638
XpXylT_MH673349
Paenibacillus_sp_JDR-2_WP_015843614
Methylorubrum_extorquens_WP_003600880

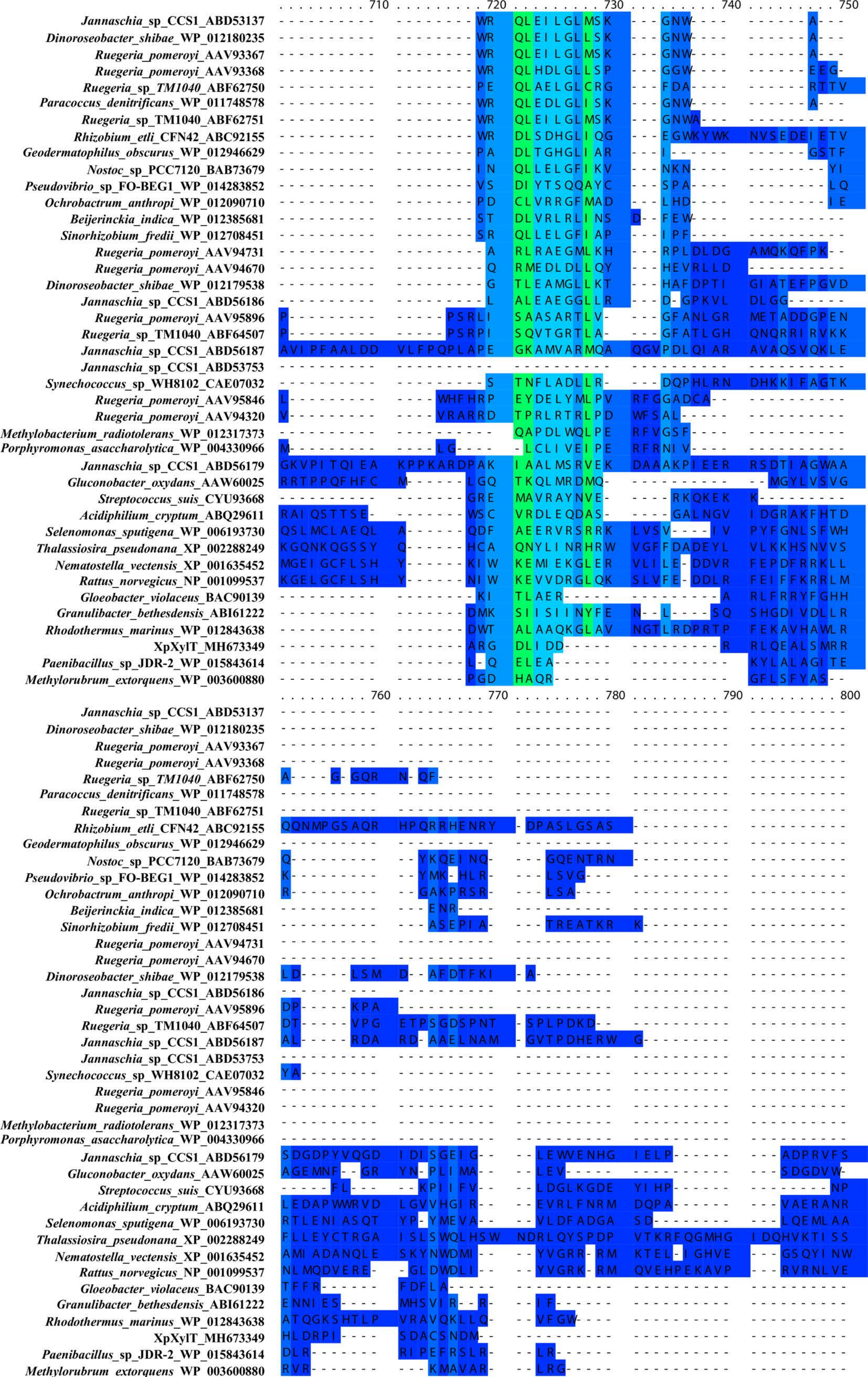

710 720 730 740 750
Jannaschia_sp_CCS1_ABD53137
Dinoroseobacter_shibae_WP_012180235
Ruegeria_pomeroyi_AAV93367
Ruegeria_pomeroyi_AAV93368
Ruegeria_sp_TM1040_ABF62750
Paracoccus_denitrificans_WP_011748578
Ruegeria_sp_TM1040_ABF62751
Rhizobium_etli_CFN42_ABC92155
Geodermatophilus_obscurus_WP_012946629
Nostoc_sp_PCC7120_BAB73679
Pseudovibrio_sp_FO-BEG1_WP_014283852
Ochrobactrum_anthropi_WP_012090710
Beijerinckia_indica_WP_012385681
Sinorhizobium_fredii_WP_012708451
Ruegeria_pomeroyi_AAV94731
Ruegeria_pomeroyi_AAV94670
Dinoroseobacter_shibae_WP_012179538
Jannaschia_sp_CCS1_ABD56186
Ruegeria_pomeroyi_AAV95896
Ruegeria_sp_TM1040_ABF64507
Jannaschia_sp_CCS1_ABD56187
Jannaschia_sp_CCS1_ABD53753
Synechococcus_sp_WH8102_CAE07032
Ruegeria_pomeroyi_AAV95846
Ruegeria_pomeroyi_AAV94320
Methylobacterium_radiotolerans_WP_012317373
Porphyromonas_asaccharolytica_WP_004330966
Jannaschia_sp_CCS1_ABD56179
Gluconobacter_oxydans_AAW60025
Streptococcus_suis_CYU93668
Acidiphilium_cryptum_ABQ29611
Selenomonas_sputigena_WP_006193730
Thalassiosira_pseudonana_XP_002288249
Nematostella_vectensis_XP_001635452
Rattus_norvegicus_NP_001099537
Gloeobacter_violaceus_BAC90139
Granulibacter_bethesdensis_ABI61222
Rhodothermus_marinus_WP_012843638
XpXylT_MH673349
Paenibacillus_sp_JDR-2_WP_015843614
Methylorubrum_extorquens_WP_003600880
760 770 780 790 800

```
                                               .........810.........820.........830.........840.........850
Jannaschia_sp_CCS1_ABD53137                    ---------- ---------- ---------- ---------- ----------
Dinoroseobacter_shibae_WP_012180235            ---------- ---------- ---------- ---------- ----------
Ruegeria_pomeroyi_AAV93367                     ---------- ---------- ---------- ---------- ----------
Ruegeria_pomeroyi_AAV93368                     ---------- ---------- ---------- ---------- ----------
Ruegeria_sp_TM1040_ABF62750                    ---------- ---------- ---------- ---------- ----------
Paracoccus_denitrificans_WP_011748578          ---------- ---------- ---------- ---------- ----------
Ruegeria_sp_TM1040_ABF62751                    ---------- ---------- ---------- ---------- ----------
Rhizobium_etli_CFN42_ABC92155                  ---------- ---------- ---------- ---------- ----------
Geodermatophilus_obscurus_WP_012946629         ---------- ---------- ---------- ---------- ----------
Nostoc_sp_PCC7120_BAB73679                     ---------- ---------- ---------- ---------- ----------
Pseudovibrio_sp_FO-BEG1_WP_014283852           ---------- ---------- ---------- ---------- ----------
Ochrobactrum_anthropi_WP_012090710             ---------- ---------- ---------- ---------- ----------
Beijerinckia_indica_WP_012385681               ---------- ---------- ---------- ---------- ----------
Sinorhizobium_fredii_WP_012708451              ---------- ---------- ---------- ---------- ----------
Ruegeria_pomeroyi_AAV94731                     ---------- ---------- ---------- ---------- ----------
Ruegeria_pomeroyi_AAV94670                     ---------- ---------- ---------- ---------- ----------
Dinoroseobacter_shibae_WP_012179538            ---------- ---------- ---------- ---------- ----------
Jannaschia_sp_CCS1_ABD56186                    ---------- ---------- ---------- ---------- ----------
Ruegeria_pomeroyi_AAV95896                     ---------- ---------- ---------- ---------- ----------
Ruegeria_sp_TM1040_ABF64507                    ---------- ---------- ---------- ---------- ----------
Jannaschia_sp_CCS1_ABD56187                    ---------- ---------- ---------- ---------- ----------
Jannaschia_sp_CCS1_ABD53753                    ---------- ---------- ---------- ---------- ----------
Synechococcus_sp_WH8102_CAE07032               ---------- ---------- ---------- ---------- ----------
Ruegeria_pomeroyi_AAV95846                     ---------- ---------- ---------- ---------- ----------
Ruegeria_pomeroyi_AAV94320                     ---------- ---------- ---------- ---------- ----------
Methylobacterium_radiotolerans_WP_012317373    ---------- ---------- ---------- ---------- ----------
Porphyromonas_asaccharolytica_WP_004330966     ---------- ---------- ---------- ---------- ----------
Jannaschia_sp_CCS1_ABD56179                    PE-------- ---------- ---------- ---------- ----SL--DA
Gluconobacter_oxydans_AAW60025                 NA-------- ---------- ---------- ---------- ----CLFTEN
Streptococcus_suis_CYU93668                    SN-------- ---------- ---------- ---------- ----HL---T
Acidiphilium_cryptum_ABQ29611                  IA-------- ---------- ---------- ---------- ----IDIGFD
Selenomonas_sputigena_WP_006193730             LHL------- ---------- ---------- ---------- ---PHCIVRA
Thalassiosira_pseudonana_XP_002288249          VDAINLTATH HPHYAFLKEG YTQLDTNGNE ISPRWQNLRF PSDVGVFFHH
Nematostella_vectensis_XP_001635452            VNY------- ---------- TWW------- ---------- -----TLGYM
Rattus_norvegicus_NP_001099537                 ADY------- ---------- SYW------- ---------- -----TLAYV
Gloeobacter_violaceus_BAC90139                 ---------- ---------- ---------- ---------- ----------
Granulibacter_bethesdensis_ABI61222            ---------- ---------- ---------- ---------- ----------
Rhodothermus_marinus_WP_012843638              ---------- ---------- ---------- ---------- ----------
XpXylT_MH673349                                ---------- ---------- ---------- ---------- ----------
Paenibacillus_sp_JDR-2_WP_015843614            ---------- ---------- ---------- ---------- ----------
Methylorubrum_extorquens_WP_003600880          ---------- ---------- ---------- ---------- ----------

                                               .........860.........870.........880.........890.........900
Jannaschia_sp_CCS1_ABD53137                    ---------- ---------- ---------- ---------- ----------
Dinoroseobacter_shibae_WP_012180235            ---------- ---------- ---------- ---------- ----------
Ruegeria_pomeroyi_AAV93367                     ---------- ---------- ---------- ---------- ----------
Ruegeria_pomeroyi_AAV93368                     ---------- ---------- ---------- ---------- ----------
Ruegeria_sp_TM1040_ABF62750                    ---------- ---------- ---------- ---------- ----------
Paracoccus_denitrificans_WP_011748578          ---------- ---------- ---------- ---------- ----------
Ruegeria_sp_TM1040_ABF62751                    ---------- ---------- ---------- ---------- ----------
Rhizobium_etli_CFN42_ABC92155                  ---------- ---------- ---------- ---------- ----------
Geodermatophilus_obscurus_WP_012946629         ---------- ---------- ---------- ---------- ----------
Nostoc_sp_PCC7120_BAB73679                     ---------- ---------- ---------- ---------- ----------
Pseudovibrio_sp_FO-BEG1_WP_014283852           ---------- ---------- ---------- ---------- ----------
Ochrobactrum_anthropi_WP_012090710             ---------- ---------- ---------- ---------- ----------
Beijerinckia_indica_WP_012385681               ---------- ---------- ---------- ---------- ----------
Sinorhizobium_fredii_WP_012708451              ---------- ---------- ---------- ---------- ----------
Ruegeria_pomeroyi_AAV94731                     ---------- ---------- ---------- ---------- ----------
Ruegeria_pomeroyi_AAV94670                     ---------- ---------- ---------- ---------- ----------
Dinoroseobacter_shibae_WP_012179538            ---------- ---------- ---------- ---------- ----------
Jannaschia_sp_CCS1_ABD56186                    ---------- ---------- ---------- ---------- ----------
Ruegeria_pomeroyi_AAV95896                     ---------- ---------- ---------- ---------- ----------
Ruegeria_sp_TM1040_ABF64507                    ---------- ---------- ---------- ---------- ----------
Jannaschia_sp_CCS1_ABD56187                    ---------- ---------- ---------- ---------- ----------
Jannaschia_sp_CCS1_ABD53753                    ---------- ---------- ---------- ---------- ----------
Synechococcus_sp_WH8102_CAE07032               ---------- ---------- ---------- ---------- ----------
Ruegeria_pomeroyi_AAV95846                     ---------- ---------- ---------- ---------- ----------
Ruegeria_pomeroyi_AAV94320                     ---------- ---------- ---------- ---------- ----------
Methylobacterium_radiotolerans_WP_012317373    ---------- ---------- ---------- ---------- ----------
Porphyromonas_asaccharolytica_WP_004330966     ---------- ---------- ---------- ---------- ----------
Jannaschia_sp_CCS1_ABD56179                    IILSRFERRH ARNIAGYLE- ---------- -------NCP RVLEIGAGIG
Gluconobacter_oxydans_AAW60025                 PLPGRYLSLP GSPT--LLP- ---------- -------MVP LHVMI-AENT
Streptococcus_suis_CYU93668                    ILTEMYNVRG LLTDNHQIK- ---------- -------FLK VNYRL-----
Acidiphilium_cryptum_ABQ29611                  PEHFIEVFRR ESDEPFGGV- ---------- -------DGN PLVFA-PSIP
Selenomonas_sputigena_WP_006193730             DSPGWGVRLA QTARGEYIQ- ---------- -------WVM PGDRLF--PE
Thalassiosira_pseudonana_XP_002288249          HCKSWKEYVA KRMRGRATMS GNALEASRKE LVQKAKNKDG FKNSTRIDPL
Nematostella_vectensis_XP_001635452            IKLEGARKLV SAKPLTKMM- ---------- ---------A IDEFL---PI
Rattus_norvegicus_NP_001099537                 ISLQGAQKLL AAKPLAKML- ---------- ---------P VDEFL---PV
Gloeobacter_violaceus_BAC90139                 ---------- ---------- ---------- ---------- ----------
Granulibacter_bethesdensis_ABI61222            ---------- ---------- ---------- ---------- ----------
Rhodothermus_marinus_WP_012843638              ---------- ---------- ---------- ---------- ----------
XpXylT_MH673349                                ---------- ---------- ---------- ---------- ----------
Paenibacillus_sp_JDR-2_WP_015843614            ---------- ---------- ---------- ---------- ----------
Methylorubrum_extorquens_WP_003600880          ---------- ---------- ---------- ---------- ----------
```

```
                                          . . . . . . . . . 910 . . . . . . . . . 920 . . . . . . . . . 930 . . . . . . . . . 940 . . . . . . . . . 950
Jannaschia_sp_CCS1_ABD53137               ---------- ---------- ---------- ---------- ----------
Dinoroseobacter_shibae_WP_012180235       ---------- ---------- ---------- ---------- ----------
Ruegeria_pomeroyi_AAV93367                ---------- ---------- ---------- ---------- ----------
Ruegeria_pomeroyi_AAV93368                ---------- ---------- ---------- ---------- ----------
Ruegeria_sp_TM1040_ABF62750               ---------- ---------- ---------- ---------- ----------
Paracoccus_denitrificans_WP_011748578     ---------- ---------- ---------- ---------- ----------
Ruegeria_sp_TM1040_ABF62751               ---------- ---------- ---------- ---------- ----------
Rhizobium_etli_CFN42_ABC92155             ---------- ---------- ---------- ---------- ----------
Geodermatophilus_obscurus_WP_012946629    ---------- ---------- ---------- ---------- ----------
Nostoc_sp_PCC7120_BAB73679                ---------- ---------- ---------- ---------- ----------
Pseudovibrio_sp_FO-BEG1_WP_014283852      ---------- ---------- ---------- ---------- ----------
Ochrobactrum_anthropi_WP_012090710        ---------- ---------- ---------- ---------- ----------
Beijerinckia_indica_WP_012385681          ---------- ---------- ---------- ---------- ----------
Sinorhizobium_fredii_WP_012708451         ---------- ---------- ---------- ---------- ----------
Ruegeria_pomeroyi_AAV94731                ---------- ---------- ---------- ---------- ----------
Ruegeria_pomeroyi_AAV94670                ---------- ---------- ---------- ---------- ----------
Dinoroseobacter_shibae_WP_012179538       ---------- ---------- ---------- ---------- ----------
Jannaschia_sp_CCS1_ABD56186               ---------- ---------- ---------- ---------- ----------
Ruegeria_pomeroyi_AAV95896                ---------- ---------- ---------- ---------- ----------
Ruegeria_sp_TM1040_ABF64507               ---------- ---------- ---------- ---------- ----------
Jannaschia_sp_CCS1_ABD56187               ---------- ---------- ---------- ---------- ----------
Jannaschia_sp_CCS1_ABD53753               ---------- ---------- ---------- ---------- ----------
Synechococcus_sp_WH8102_CAE07032          ---------- ---------- ---------- ---------- ----------
Ruegeria_pomeroyi_AAV95846                ---------- ---------- ---------- ---------- ----------
Ruegeria_pomeroyi_AAV94320                ---------- ---------- ---------- ---------- ----------
Methylobacterium_radiotolerans_WP_012317373 ---------- ---------- ---------- ---------- ----------
Porphyromonas_asaccharolytica_WP_004330966 ---------- ---------- ---------- ---------- ----------
Jannaschia_sp_CCS1_ABD56179               FIGMRMVKMS EGL------- ---------- -----VYMAQ DGRA------
Gluconobacter_oxydans_AAW60025            VLSPVSGE-- ---------- ---------- -----EIRIA ----------
Streptococcus_suis_CYU93668               IITPDFAKFL PHE------- ---------- -----FIVV- ---P------
Acidiphilium_cryptum_ABQ29611             IPGRFVRVRL LER------- ---------- -----NYLHL ----------
Selenomonas_sputigena_WP_006193730        KILQM--VTT LELDDEL--- ---------- ----SFVLAD ----------
Thalassiosira_pseudonana_XP_002288249     AWTKLKSVSI KYEVFESSSL HFRQSSNHTS SKKSTAICSV VCDQEAYVDE
Nematostella_vectensis_XP_001635452       MYDKHPNAEW SAHFSP---- ---------- --RNLVAMTA ----EPLLL-
Rattus_norvegicus_NP_001099537            MFDKHPMSEY KSHFSP---- ---------- --RNLRAFSV ----EPLLI-
Gloeobacter_violaceus_BAC90139            ---------- ---------- ---------- ---------- ----------
Granulibacter_bethesdensis_ABI61222       ---------- ---------- ---------- ---------- ----------
Rhodothermus_marinus_WP_012843638         ---------- ---------- ---------- ---------- ----------
XpXylT_MH673349                           ---------- ---------- ---------- ---------- ----------
Paenibacillus_sp_JDR-2_WP_015843614       ---------- ---------- ---------- ---------- ----------
Methylorubrum_extorquens_WP_003600880     ---------- ---------- ---------- ---------- ----------
                                          . . . . . . . . . 960 . . . . . . . . . 970 . . . . . . . . . 980 . . . . . . . . . 990 . . . . . . . . . 1000
Jannaschia_sp_CCS1_ABD53137               ---------- ---------- ---------- ---------- ----------
Dinoroseobacter_shibae_WP_012180235       ---------- ---------- ---------- ---------- ----------
Ruegeria_pomeroyi_AAV93367                ---------- ---------- ---------- ---------- ----------
Ruegeria_pomeroyi_AAV93368                ---------- ---------- ---------- ---------- ----------
Ruegeria_sp_TM1040_ABF62750               ---------- ---------- ---------- ---------- ----------
Paracoccus_denitrificans_WP_011748578     ---------- ---------- ---------- ---------- ----------
Ruegeria_sp_TM1040_ABF62751               ---------- ---------- ---------- ---------- ----------
Rhizobium_etli_CFN42_ABC92155             ---------- ---------- ---------- ---------- ----------
Geodermatophilus_obscurus_WP_012946629    ---------- ---------- ---------- ---------- ----------
Nostoc_sp_PCC7120_BAB73679                ---------- ---------- ---------- ---------- ----------
Pseudovibrio_sp_FO-BEG1_WP_014283852      ---------- ---------- ---------- ---------- ----------
Ochrobactrum_anthropi_WP_012090710        ---------- ---------- ---------- ---------- ----------
Beijerinckia_indica_WP_012385681          ---------- ---------- ---------- ---------- ----------
Sinorhizobium_fredii_WP_012708451         ---------- ---------- ---------- ---------- ----------
Ruegeria_pomeroyi_AAV94731                ---------- ---------- ---------- ---------- ----------
Ruegeria_pomeroyi_AAV94670                ---------- ---------- ---------- ---------- ----------
Dinoroseobacter_shibae_WP_012179538       ---------- ---------- ---------- ---------- ----------
Jannaschia_sp_CCS1_ABD56186               ---------- ---------- ---------- ---------- ----------
Ruegeria_pomeroyi_AAV95896                ---------- ---------- ---------- ---------- ----------
Ruegeria_sp_TM1040_ABF64507               ---------- ---------- ---------- ---------- ----------
Jannaschia_sp_CCS1_ABD56187               ---------- ---------- ---------- ---------- ----------
Jannaschia_sp_CCS1_ABD53753               ---------- ---------- ---------- ---------- ----------
Synechococcus_sp_WH8102_CAE07032          ---------- ---------- ---------- ---------- ----------
Ruegeria_pomeroyi_AAV95846                ---------- ---------- ---------- ---------- ----------
Ruegeria_pomeroyi_AAV94320                ---------- ---------- ---------- ---------- ----------
Methylobacterium_radiotolerans_WP_012317373 ---------- ---------- ---------- ---------- ----------
Porphyromonas_asaccharolytica_WP_004330966 ---------- ---------- ---------- ---------- ----------
Jannaschia_sp_CCS1_ABD56179               ---------- -PLVRYGRDL AGRADVASDR LKFTDGPLVF PGD-------
Gluconobacter_oxydans_AAW60025            ---------- ---IP-GHAL S--------- ---------- ----------
Streptococcus_suis_CYU93668               ---------- -DTLDI-EQV KSQYVGTGVD LS----KII- ----------
Acidiphilium_cryptum_ABQ29611             ---------- -DQVEVYGDV LAQFG----- ---------- ----------
Selenomonas_sputigena_WP_006193730        ---------- -AEETATAEG ADPERFVFSM HRSHCFVAAG A---------
Thalassiosira_pseudonana_XP_002288249     WIDYHLALGV SAVYVLDSSE DFWMRQWGEE RGQTAPIVVT HFPGNVTNPS
Nematostella_vectensis_XP_001635452       ---------- -YPTHYIGDK GYFSDTETAA FVPEEIPTLT SW--------
Rattus_norvegicus_NP_001099537            ---------- -YPTHYTGDD GYVSDTETSV VWNNEQ-VKT DW--------
Gloeobacter_violaceus_BAC90139            ---------- ---------- ---------- ---------- ----------
Granulibacter_bethesdensis_ABI61222       ---------- ---------- ---------- ---------- ----------
Rhodothermus_marinus_WP_012843638         ---------- ---------- ---------- ---------- ----------
XpXylT_MH673349                           ---------- ---------- ---------- ---------- ----------
Paenibacillus_sp_JDR-2_WP_015843614       ---------- ---------- ---------- ---------- ----------
Methylorubrum_extorquens_WP_003600880     ---------- ---------- ---------- ---------- ----------
```

```
                                          .........1010.........1020.........1030.........1040.........1050
Jannaschia_sp_CCS1_ABD53137               ---------- ---------- ---------- ---------- ----------
Dinoroseobacter_shibae_WP_012180235       ---------- ---------- ---------- ---------- ----------
Ruegeria_pomeroyi_AAV93367                ---------- ---------- ---------- ---------- ----------
Ruegeria_pomeroyi_AAV93368                ---------- ---------- ---------- ---------- ----------
Ruegeria_sp_TM1040_ABF62750               ---------- ---------- ---------- ---------- ----------
Paracoccus_denitrificans_WP_011748578     ---------- ---------- ---------- ---------- ----------
Ruegeria_sp_TM1040_ABF62751               ---------- ---------- ---------- ---------- ----------
Rhizobium_etli_CFN42_ABC92155             ---------- ---------- ---------- ---------- ----------
Geodermatophilus_obscurus_WP_012946629    ---------- ---------- ---------- ---------- ----------
Nostoc_sp_PCC7120_BAB73679                ---------- ---------- ---------- ---------- ----------
Pseudovibrio_sp_FO-BEG1_WP_014283852      ---------- ---------- ---------- ---------- ----------
Ochrobactrum_anthropi_WP_012090710        ---------- ---------- ---------- ---------- ----------
Beijerinckia_indica_WP_012385681          ---------- ---------- ---------- ---------- ----------
Sinorhizobium_fredii_WP_012708451         ---------- ---------- ---------- ---------- ----------
Ruegeria_pomeroyi_AAV94731                ---------- ---------- ---------- ---------- ----------
Ruegeria_pomeroyi_AAV94670                ---------- ---------- ---------- ---------- ----------
Dinoroseobacter_shibae_WP_012179538       ---------- ---------- ---------- ---------- ----------
Jannaschia_sp_CCS1_ABD56186               ---------- ---------- ---------- ---------- ----------
Ruegeria_pomeroyi_AAV95896                ---------- ---------- ---------- ---------- ----------
Ruegeria_sp_TM1040_ABF64507               ---------- ---------- ---------- ---------- ----------
Jannaschia_sp_CCS1_ABD56187               ---------- ---------- ---------- ---------- ----------
Jannaschia_sp_CCS1_ABD53753               ---------- ---------- ---------- ---------- ----------
Synechococcus_sp_WH8102_CAE07032          ---------- ---------- ---------- ---------- ----------
Ruegeria_pomeroyi_AAV95846                ---------- ---------- ---------- ---------- ----------
Ruegeria_pomeroyi_AAV94320                ---------- ---------- ---------- ---------- ----------
Methylobacterium_radiotolerans_WP_012317373 ---------- ---------- ---------- ---------- ----------
Porphyromonas_asaccharolytica_WP_004330966 ---------- ---------- ---------- ---------- ----------
Jannaschia_sp_CCS1_ABD56179               WAAQASGLAA YLADFKPDAL RIGR------ ---------A SDLPAE----
Gluconobacter_oxydans_AAW60025            KIDATTALYT RMTSF---MV LTAE------ ---------G HGL-AD----
Streptococcus_suis_CYU93668               SLKEYRKEIG FIGNL----- ---------- ---------- ----YA----
Acidiphilium_cryptum_ABQ29611             ---------- ---------- ---------- ---------- ----------
Selenomonas_sputigena_WP_006193730        WHRAYALAAG RTPMGGLSGV LLRR-RVLDA CAWLELAFFR KRFFPLAA--
Thalassiosira_pseudonana_XP_002288249     YKAKAYAKCL ALHRHDHQAM VFFDVNDFLV FPDGKGLSSV DHLVSSTSSC
Nematostella_vectensis_XP_001635452       SLAKRQQLRF KQ-------- ---------- ---------- ----------
Rattus_norvegicus_NP_001099537            DRAKSQKMRE QQALSR---- -EAKNSDVLQ SPLDS--TAR DEL-------
Gloeobacter_violaceus_BAC90139            ---------- ---------- ---------- ---------- ----------
Granulibacter_bethesdensis_ABI61222       ---------- ---------- ---------- ---------- ----------
Rhodothermus_marinus_WP_012843638         ---------- ---------- ---------- ---------- ----------
XpXylT_MH673349                           ---------- ---------- ---------- ---------- ----------
Paenibacillus_sp_JDR-2_WP_015843614       ---------- ---------- ---------- ---------- ----------
Methylorubrum_extorquens_WP_003600880     ---------- ---------- ---------- ---------- ----------

                                          .........1060.........1070.........1080.........1090.........1100
Jannaschia_sp_CCS1_ABD53137               ---------- ---------- ---------- ---------- ----------
Dinoroseobacter_shibae_WP_012180235       ---------- ---------- ---------- ---------- ----------
Ruegeria_pomeroyi_AAV93367                ---------- ---------- ---------- ---------- ----------
Ruegeria_pomeroyi_AAV93368                ---------- ---------- ---------- ---------- ----------
Ruegeria_sp_TM1040_ABF62750               ---------- ---------- ---------- ---------- ----------
Paracoccus_denitrificans_WP_011748578     ---------- ---------- ---------- ---------- ----------
Ruegeria_sp_TM1040_ABF62751               ---------- ---------- ---------- ---------- ----------
Rhizobium_etli_CFN42_ABC92155             ---------- ---------- ---------- ---------- ----------
Geodermatophilus_obscurus_WP_012946629    ---------- ---------- ---------- ---------- ----------
Nostoc_sp_PCC7120_BAB73679                ---------- ---------- ---------- ---------- ----------
Pseudovibrio_sp_FO-BEG1_WP_014283852      ---------- ---------- ---------- ---------- ----------
Ochrobactrum_anthropi_WP_012090710        ---------- ---------- ---------- ---------- ----------
Beijerinckia_indica_WP_012385681          ---------- ---------- ---------- ---------- ----------
Sinorhizobium_fredii_WP_012708451         ---------- ---------- ---------- ---------- ----------
Ruegeria_pomeroyi_AAV94731                ---------- ---------- ---------- ---------- ----------
Ruegeria_pomeroyi_AAV94670                ---------- ---------- ---------- ---------- ----------
Dinoroseobacter_shibae_WP_012179538       ---------- ---------- ---------- ---------- ----------
Jannaschia_sp_CCS1_ABD56186               ---------- ---------- ---------- ---------- ----------
Ruegeria_pomeroyi_AAV95896                ---------- ---------- ---------- ---------- ----------
Ruegeria_sp_TM1040_ABF64507               ---------- ---------- ---------- ---------- ----------
Jannaschia_sp_CCS1_ABD56187               ---------- ---------- ---------- ---------- ----------
Jannaschia_sp_CCS1_ABD53753               ---------- ---------- ---------- ---------- ----------
Synechococcus_sp_WH8102_CAE07032          ---------- ---------- ---------- ---------- ----------
Ruegeria_pomeroyi_AAV95846                ---------- ---------- ---------- ---------- ----------
Ruegeria_pomeroyi_AAV94320                ---------- ---------- ---------- ---------- ----------
Methylobacterium_radiotolerans_WP_012317373 ---------- ---------- ---------- ---------- ----------
Porphyromonas_asaccharolytica_WP_004330966 ---------- ---------- ---------- ---------- ----------
Jannaschia_sp_CCS1_ABD56179               -------ILA NQD-LIGLSR IIIP-VIS-- ----DAQADA YRADYAPVLT
Gluconobacter_oxydans_AAW60025            -------LLR GIDRLPAPDA SALGCAIA-- ------MLDP EDAERLAQ-T
Streptococcus_suis_CYU93668               -------LLG FVPNM--LNR IYL--YI--- ------QRNG IAKTIIKI-K
Acidiphilium_cryptum_ABQ29611             ---------- ---------- ---------- ---------- ----------
Selenomonas_sputigena_WP_006193730        --FVLLFQME KDFRAGVFDV PLL--SHEFH DEGTLVWHPA EWASLLLSCG
Thalassiosira_pseudonana_XP_002288249     AFQIQRVVFG NSGQFVFDPL PVAKRFMFRV ESKRELIHPA LILHVGAERM
Nematostella_vectensis_XP_001635452       ---------- ---------- ---------- ---------- ----------
Rattus_norvegicus_NP_001099537            ---------- ---------- ---------- ---------- ----------
Gloeobacter_violaceus_BAC90139            ---------- ---------- ---------- ---------- ----------
Granulibacter_bethesdensis_ABI61222       ---------- ---------- ---------- ---------- ----------
Rhodothermus_marinus_WP_012843638         ---------- ---------- ---------- ---------- ----------
XpXylT_MH673349                           ---------- ---------- ---------- ---------- ----------
Paenibacillus_sp_JDR-2_WP_015843614       ---------- ---------- ---------- ---------- ----------
Methylorubrum_extorquens_WP_003600880     ---------- ---------- ---------- ---------- ----------
```

```
                                            .........1110.........1120.........1130.........1140.........1150
Jannaschia_sp_CCS1_ABD53137                 ---------- ---------- ---------- ---------- ----------
Dinoroseobacter_shibae_WP_012180235         ---------- ---------- ---------- ---------- ----------
Ruegeria_pomeroyi_AAV93367                  ---------- ---------- ---------- ---------- ----------
Ruegeria_pomeroyi_AAV93368                  ---------- ---------- ---------- ---------- ----------
Ruegeria_sp_TM1040_ABF62750                 ---------- ---------- ---------- ---------- ----------
Paracoccus_denitrificans_WP_011748578       ---------- ---------- ---------- ---------- ----------
Ruegeria_sp_TM1040_ABF62751                 ---------- ---------- ---------- ---------- ----------
Rhizobium_etli_CFN42_ABC92155               ---------- ---------- ---------- ---------- ----------
Geodermatophilus_obscurus_WP_012946629      ---------- ---------- ---------- ---------- ----------
Nostoc_sp_PCC7120_BAB73679                  ---------- ---------- ---------- ---------- ----------
Pseudovibrio_sp_FO-BEG1_WP_014283852        ---------- ---------- ---------- ---------- ----------
Ochrobactrum_anthropi_WP_012090710          ---------- ---------- ---------- ---------- ----------
Beijerinckia_indica_WP_012385681            ---------- ---------- ---------- ---------- ----------
Sinorhizobium_fredii_WP_012708451           ---------- ---------- ---------- ---------- ----------
Ruegeria_pomeroyi_AAV94731                  ---------- ---------- ---------- ---------- ----------
Ruegeria_pomeroyi_AAV94670                  ---------- ---------- ---------- ---------- ----------
Dinoroseobacter_shibae_WP_012179538         ---------- ---------- ---------- ---------- ----------
Jannaschia_sp_CCS1_ABD56186                 ---------- ---------- ---------- ---------- ----------
Ruegeria_pomeroyi_AAV95896                  ---------- ---------- ---------- ---------- ----------
Ruegeria_sp_TM1040_ABF64507                 ---------- ---------- ---------- ---------- ----------
Jannaschia_sp_CCS1_ABD56187                 ---------- ---------- ---------- ---------- ----------
Jannaschia_sp_CCS1_ABD53753                 ---------- ---------- ---------- ---------- ----------
Synechococcus_sp_WH8102_CAE07032            ---------- ---------- ---------- ---------- ----------
Ruegeria_pomeroyi_AAV95846                  ---------- ---------- ---------- ---------- ----------
Ruegeria_pomeroyi_AAV94320                  ---------- ---------- ---------- ---------- ----------
Methylobacterium_radiotolerans_WP_012317373 ---------- ---------- ---------- ---------- ----------
Porphyromonas_asaccharolytica_WP_004330966  ---------- ---------- ---------- ---------- ----------
Jannaschia_sp_CCS1_ABD56179                 DK-------- ---------- ---------- ----GFQEDP DRASAGSLQF
Gluconobacter_oxydans_AAW60025              FP-------- ---------- ---------- ----GLIPRS LMPVRPPQP-
Streptococcus_suis_CYU93668                 -S-------- ---------- ---------- ----RL---- ----------
Acidiphilium_cryptum_ABQ29611               ---------- ---------- ---------- ---------- ----------
Selenomonas_sputigena_WP_006193730          QE----LAAH SFVEARQ--- ---------- ----TLAERA REVFAREELR
Thalassiosira_pseudonana_XP_002288249       GNDSLGMLEY EISEYLVSGA WKSEICTGHT SVPNNIAAYH YLRSVKECMK
Nematostella_vectensis_XP_001635452         ---------- ---------- ---------- ---------- ----------
Rattus_norvegicus_NP_001099537              ---------- ---------- ---------- ---------- ----------
Gloeobacter_violaceus_BAC90139              ---------- ---------- ---------- ---------- ----------
Granulibacter_bethesdensis_ABI61222         ---------- ---------- ---------- ---------- ----------
Rhodothermus_marinus_WP_012843638           ---------- ---------- ---------- ---------- ----------
XpXylT_MH673349                             ---------- ---------- ---------- ---------- ----------
Paenibacillus_sp_JDR-2_WP_015843614         ---------- ---------- ---------- ---------- ----------
Methylorubrum_extorquens_WP_003600880       ---------- ---------- ---------- ---------- ----------

                                            .........1160.........1170.........1180.......
Jannaschia_sp_CCS1_ABD53137                 ---------- ---------- ---------- -------
Dinoroseobacter_shibae_WP_012180235         ---------- ---------- ---------- -------
Ruegeria_pomeroyi_AAV93367                  ---------- ---------- ---------- -------
Ruegeria_pomeroyi_AAV93368                  ---------- ---------- ---------- -------
Ruegeria_sp_TM1040_ABF62750                 ---------- ---------- ---------- -------
Paracoccus_denitrificans_WP_011748578       ---------- ---------- ---------- -------
Ruegeria_sp_TM1040_ABF62751                 ---------- ---------- ---------- -------
Rhizobium_etli_CFN42_ABC92155               ---------- ---------- ---------- -------
Geodermatophilus_obscurus_WP_012946629      ---------- ---------- ---------- -------
Nostoc_sp_PCC7120_BAB73679                  ---------- ---------- ---------- -------
Pseudovibrio_sp_FO-BEG1_WP_014283852        ---------- ---------- ---------- -------
Ochrobactrum_anthropi_WP_012090710          ---------- ---------- ---------- -------
Beijerinckia_indica_WP_012385681            ---------- ---------- ---------- -------
Sinorhizobium_fredii_WP_012708451           ---------- ---------- ---------- -------
Ruegeria_pomeroyi_AAV94731                  ---------- ---------- ---------- -------
Ruegeria_pomeroyi_AAV94670                  ---------- ---------- ---------- -------
Dinoroseobacter_shibae_WP_012179538         ---------- ---------- ---------- -------
Jannaschia_sp_CCS1_ABD56186                 ---------- ---------- ---------- -------
Ruegeria_pomeroyi_AAV95896                  ---------- ---------- ---------- -------
Ruegeria_sp_TM1040_ABF64507                 ---------- ---------- ---------- -------
Jannaschia_sp_CCS1_ABD56187                 ---------- ---------- ---------- -------
Jannaschia_sp_CCS1_ABD53753                 ---------- ---------- ---------- -------
Synechococcus_sp_WH8102_CAE07032            ---------- ---------- ---------- -------
Ruegeria_pomeroyi_AAV95846                  ---------- ---------- ---------- -------
Ruegeria_pomeroyi_AAV94320                  ---------- ---------- ---------- -------
Methylobacterium_radiotolerans_WP_012317373 ---------- ---------- ---------- -------
Porphyromonas_asaccharolytica_WP_004330966  ---------- ---------- ---------- -------
Jannaschia_sp_CCS1_ABD56179                 NRAS------ ---------- ---------- -------
Gluconobacter_oxydans_AAW60025              ---------- ---------- ---------- -------
Streptococcus_suis_CYU93668                 ---------- ---------- ---------- -------
Acidiphilium_cryptum_ABQ29611               ---------- ---------- ---------- -------
Selenomonas_sputigena_WP_006193730          AHADFS---- ---RARSIFE ELFAAL-PE- -------
Thalassiosira_pseudonana_XP_002288249       DRGDEQLCNL KGTVEDQFAW MQMQRLMPDY SNFNGFL
Nematostella_vectensis_XP_001635452         ---------- ---------- ---------- -------
Rattus_norvegicus_NP_001099537              ---------- ---------- ---------- -------
Gloeobacter_violaceus_BAC90139              ---------- ---------- ---------- -------
Granulibacter_bethesdensis_ABI61222         ---------- ---------- ---------- -------
Rhodothermus_marinus_WP_012843638           ---------- ---------- ---------- -------
XpXylT_MH673349                             ---------- ---------- ---------- -------
Paenibacillus_sp_JDR-2_WP_015843614         ---------- ---------- ---------- -------
Methylorubrum_extorquens_WP_003600880       ---------- ---------- ---------- -------
```
