# Supplementary material for: Identification of an apiosyltransferase in the plant pathogen Xanthomonas pisi
Source: PLoS One. 2018 Oct 18;13(10):e0206187. doi: 10.1371/journal.pone.0206187 (PMC6193724; doi:10.1371/journal.pone.0206187)
Supplement: S1 Table — Obtained from IDT. (DOCX) [file pone.0206187.s003.docx]

**S1 Table. Primers used in plasmid generation. Obtained from IDT.**

| Primer | Sequence (5’-3’) |
| --- | --- |
| XpXylT_F | GTATTTTCAGGGCGCCATGACAGGCATGCGTCTGATAC |
| XpXylT_R | AGCCGGATCGAATTCACTACATGTCATTGCTGCAGGC |
| XpApiT _F | GTATTTTCAGGGCGCCATGACATCGAGCTCCGCCAAAG |
| XpApiT _R | AGCCGGATCGAATTCACCGAGGCCCCGCATGCG |
| BtbGlyT046_F | GTATTTTCAGGGCGCCATGTGAGTACTCCTATTATTGCTTATG |
| BtbGlyT04_R | AGCCGGATCGAATTCACTAGCATTCATCTGTATTCTTTTCTTCCTTTC |
| pET28b_TEV_F | TGAATTCGATCCGGCTGCTAACAAAGCCCG |
| pET28b_TEV_R | CATGGCGCCCTGAAAATACAGGTTTTC |
| XpSig70_F | GTTGTAGCGATTACCGCCCG |
| XpSig70_R | GCTCAGGCGCAATTTGGC |
|  |  |
